# Supplementary figures and images for: CTHRC1 promotes anaplastic thyroid cancer progression by upregulating the proliferation, migration, and invasion of tumor cells (part 1 of 2)
Source: PeerJ. 2023 May 29;11:e15458. doi: 10.7717/peerj.15458 (PMC10234271; doi:10.7717/peerj.15458)

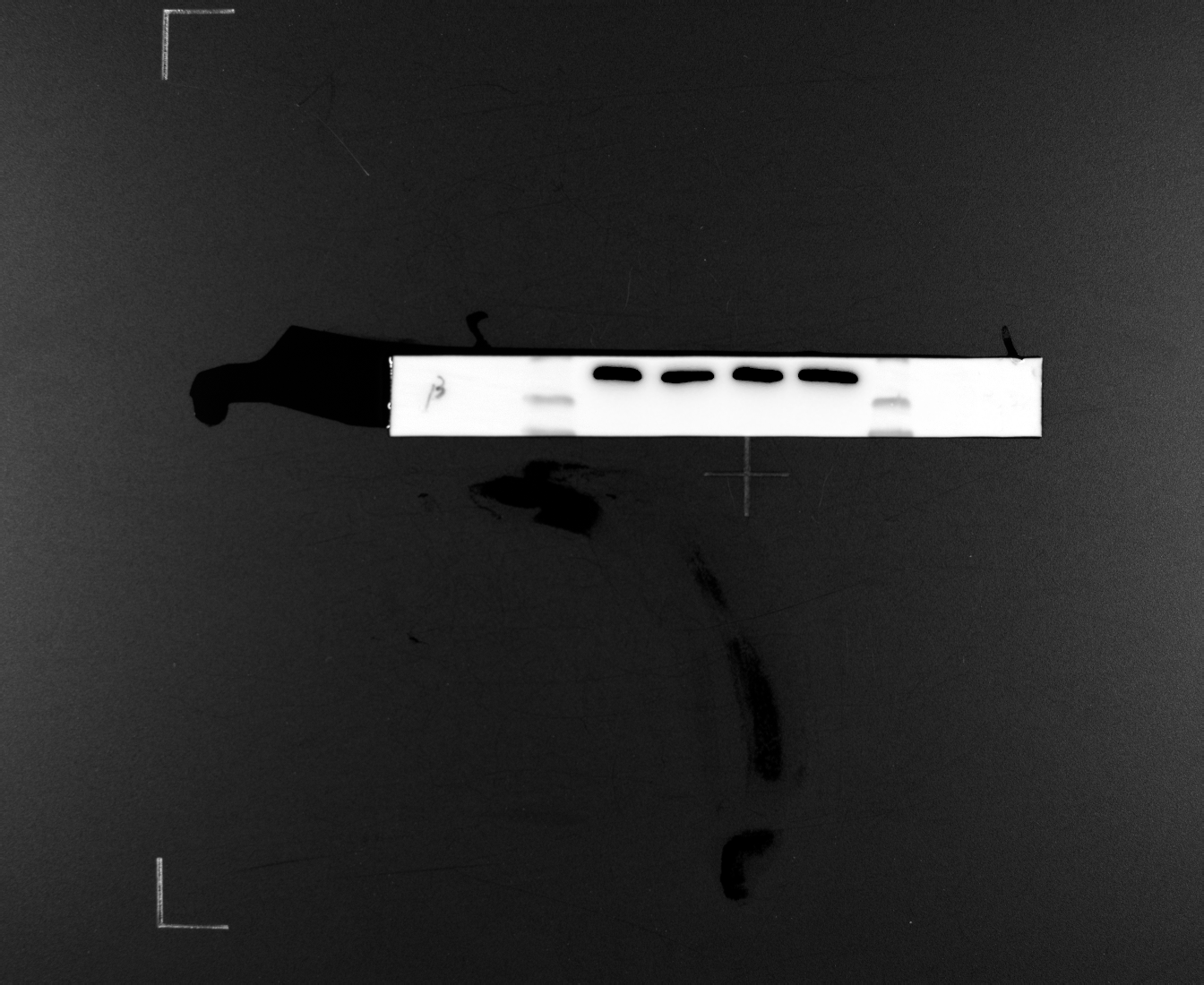

Supplement: Supplemental Information 1 [file peerj-11-15458-s001.zip › RawDataFig1/Fig1/Fig1F/actin 01.png]

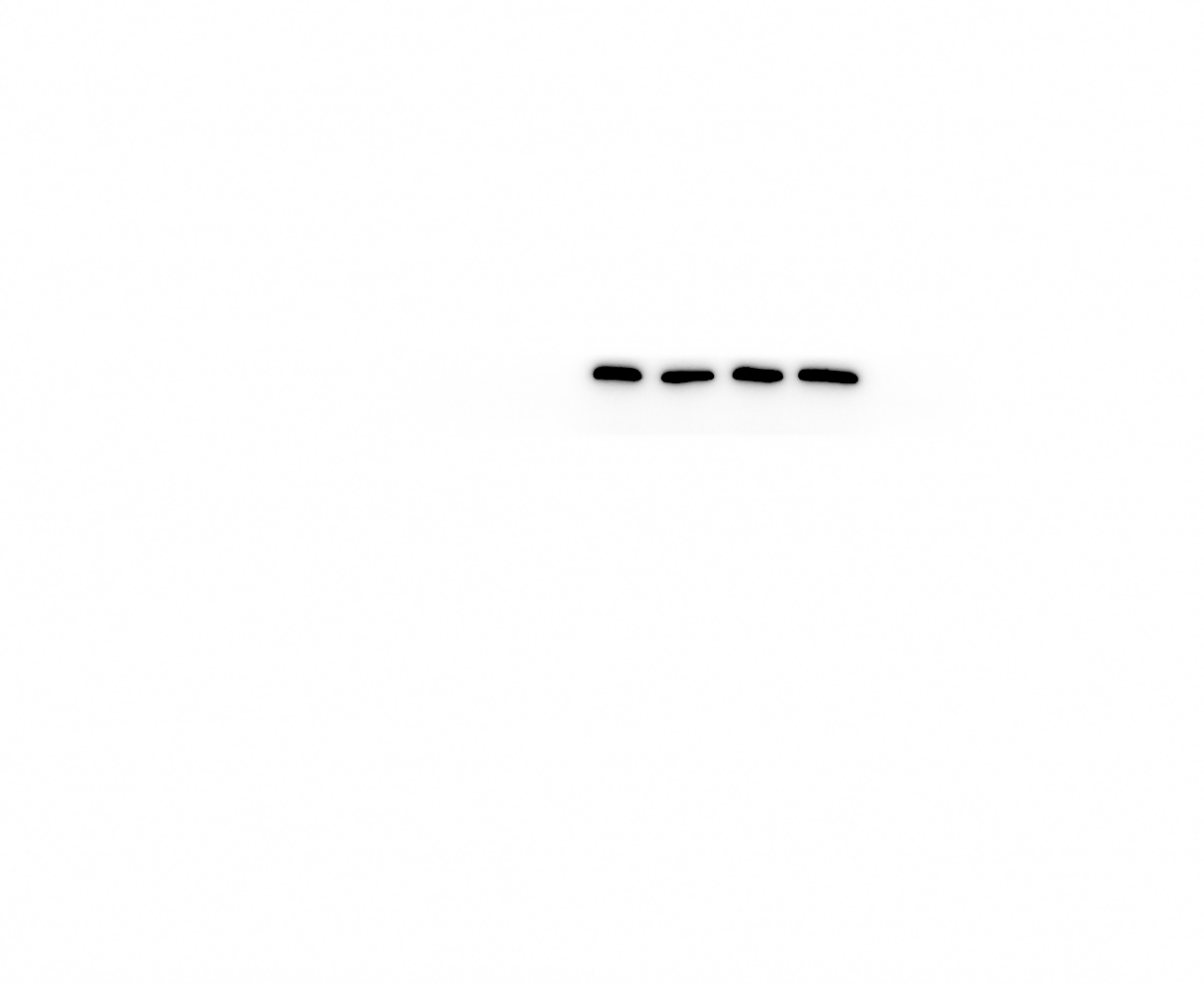

Supplement: Supplemental Information 1 [file peerj-11-15458-s001.zip › RawDataFig1/Fig1/Fig1F/actin 02.png]

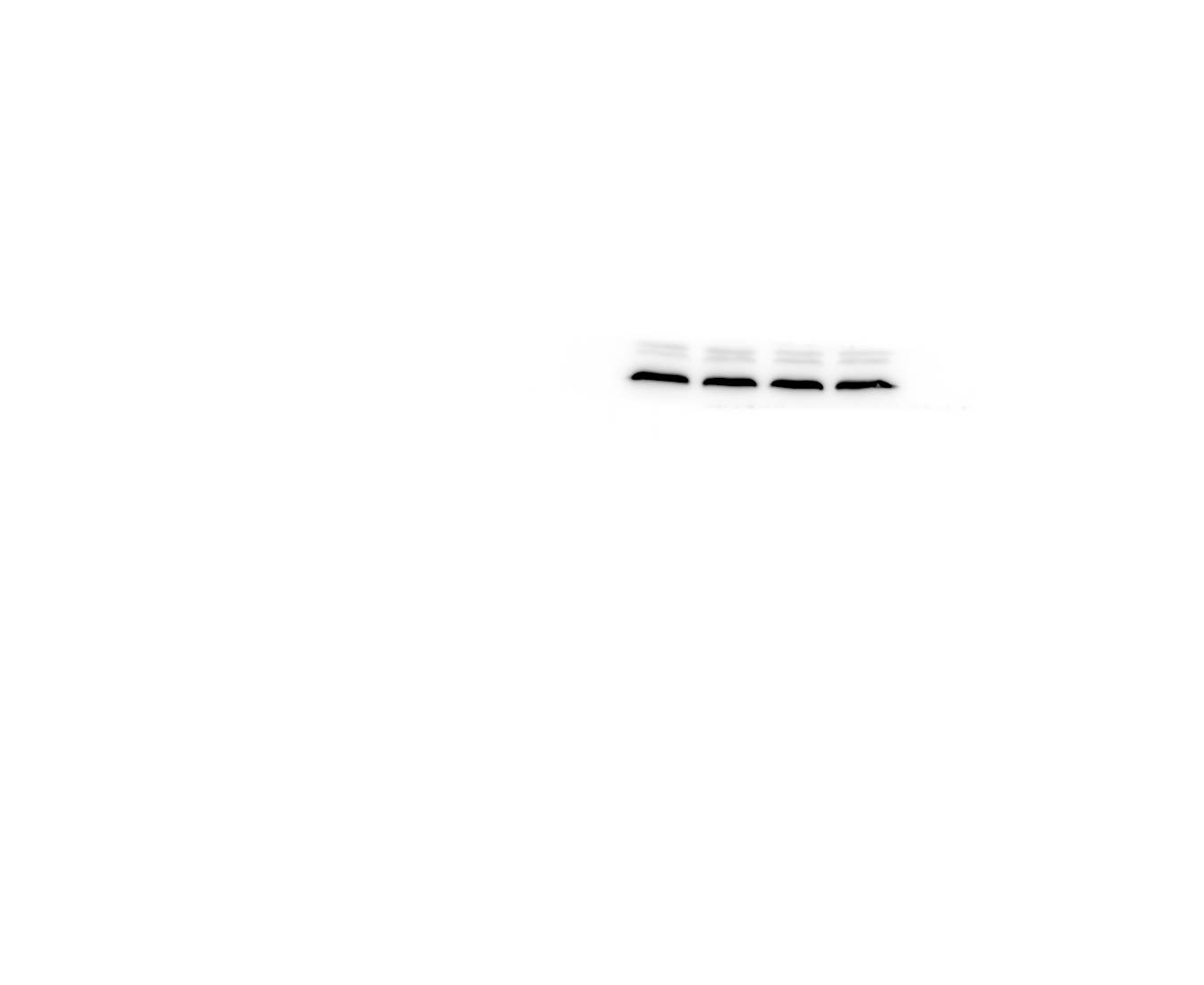

Supplement: Supplemental Information 1 [file peerj-11-15458-s001.zip › RawDataFig1/Fig1/Fig1F/actin 03.png]

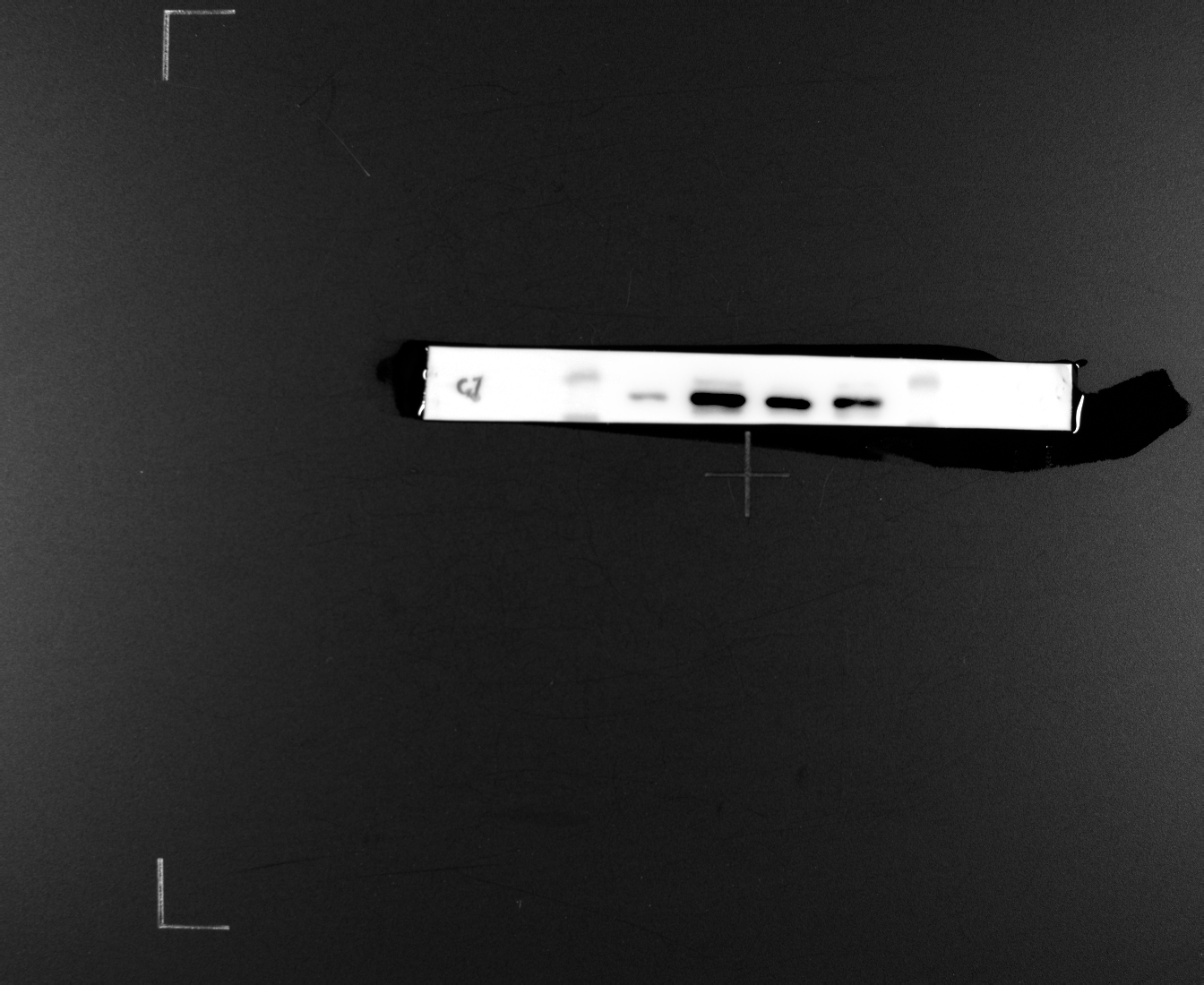

Supplement: Supplemental Information 1 [file peerj-11-15458-s001.zip › RawDataFig1/Fig1/Fig1F/CTHRC1 01.png]

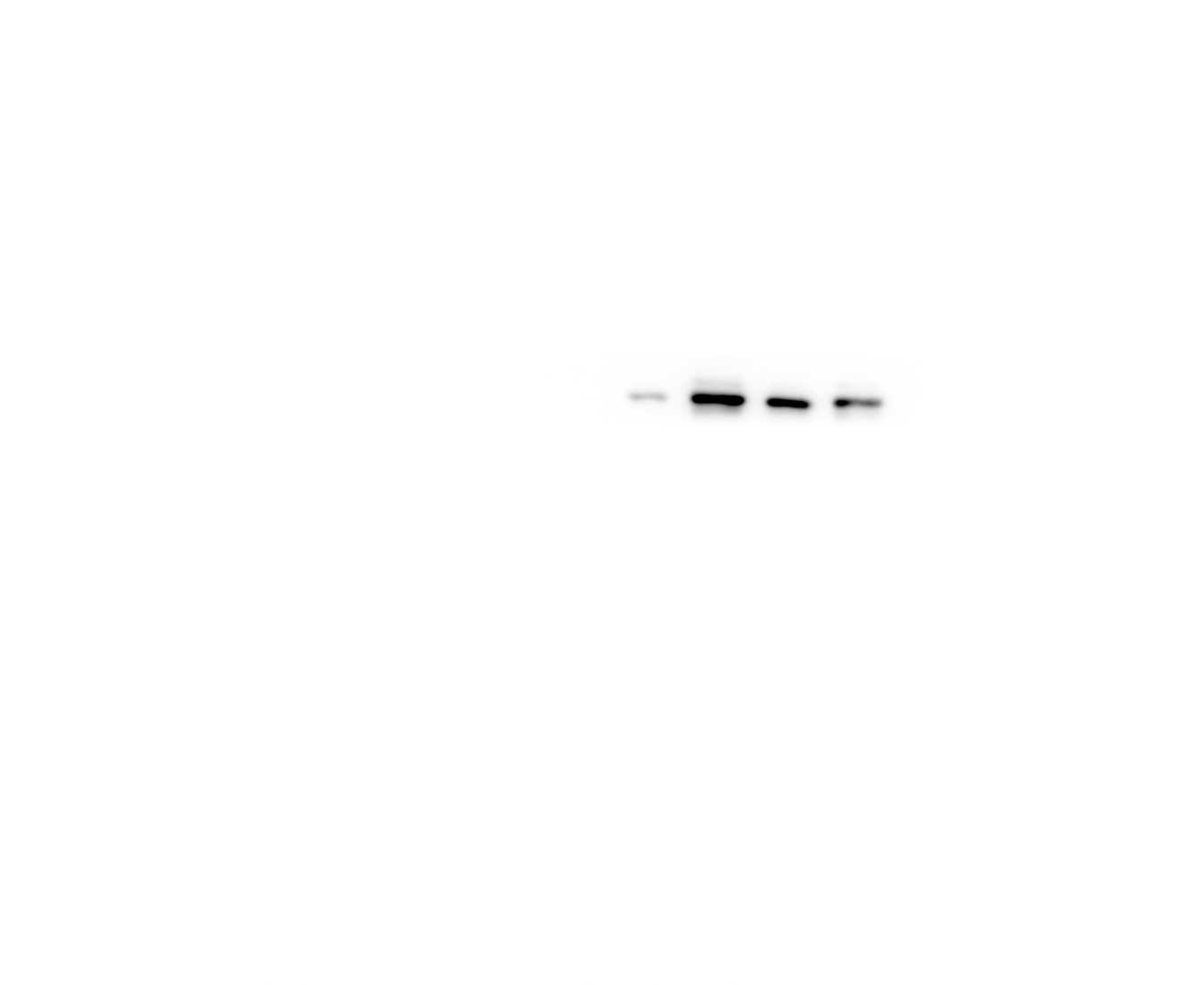

Supplement: Supplemental Information 1 [file peerj-11-15458-s001.zip › RawDataFig1/Fig1/Fig1F/CTHRC1 02.png]

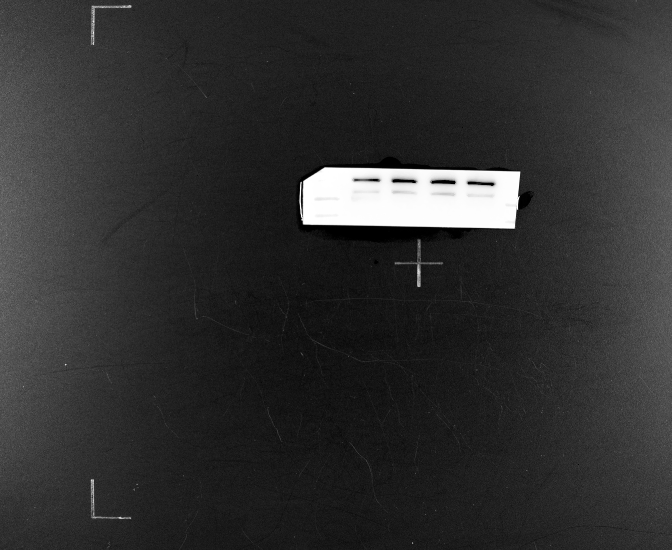

Supplement: Supplemental Information 1 [file peerj-11-15458-s001.zip › RawDataFig1/Fig1/Fig1F/CTHRC1 03.png]

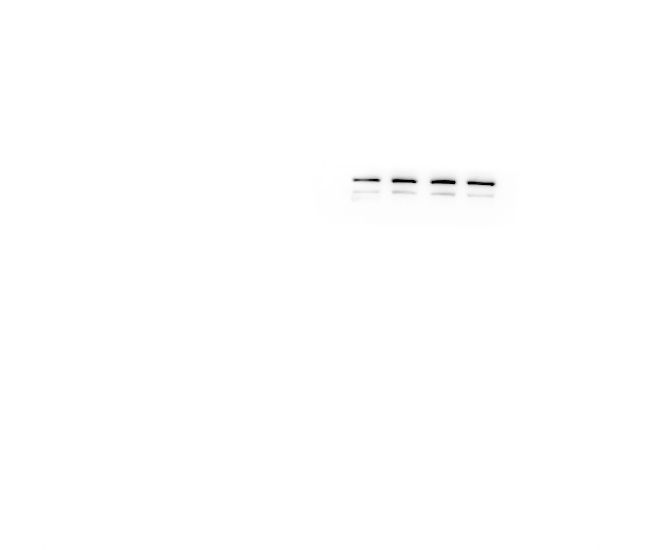

Supplement: Supplemental Information 1 [file peerj-11-15458-s001.zip › RawDataFig1/Fig1/Fig1F/CTHRC1 04.png]

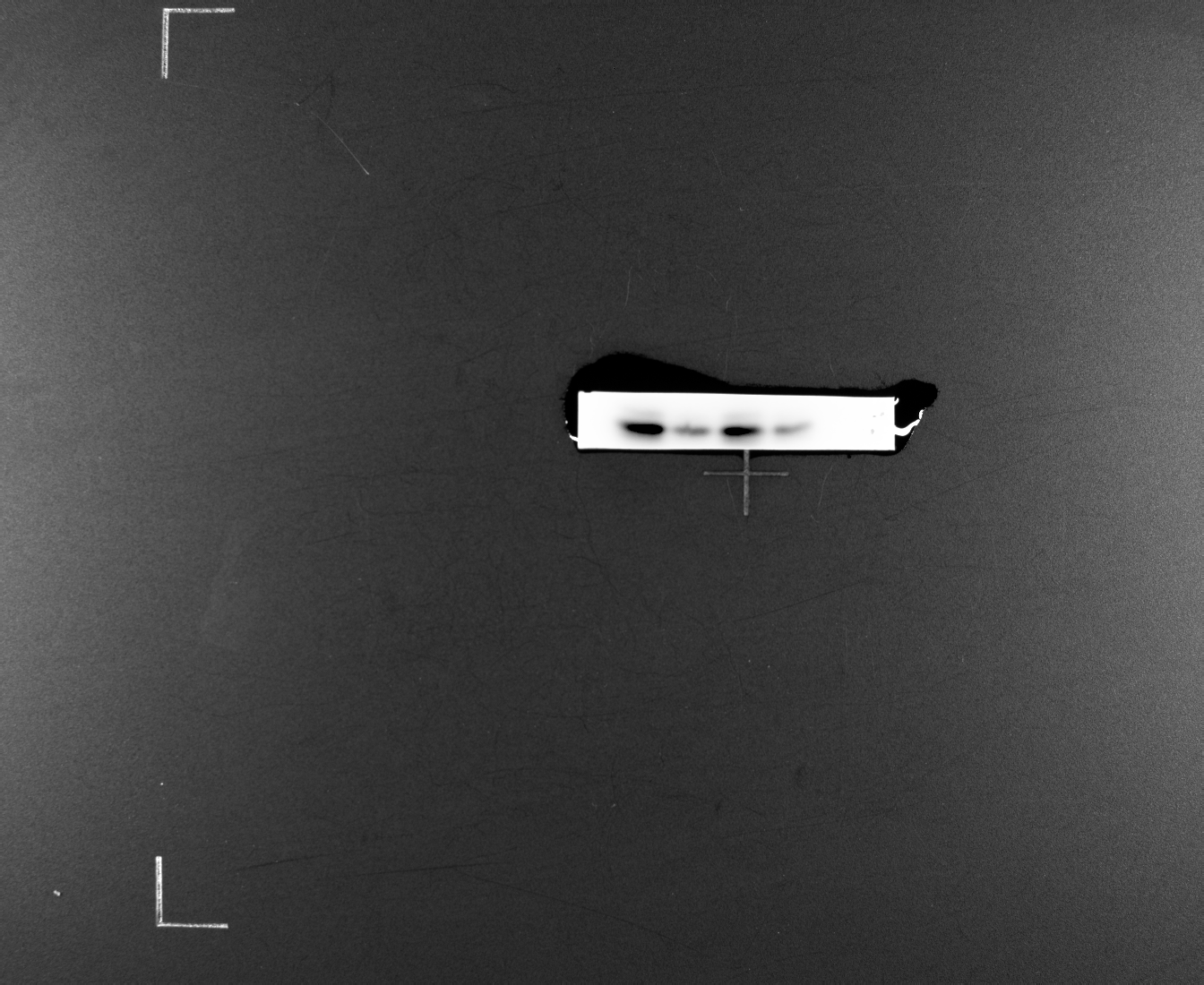

Supplement: Supplemental Information 2 [file peerj-11-15458-s002.zip › RawDataFig2/Fig2/Fig2B/CTHRC1 01.png]

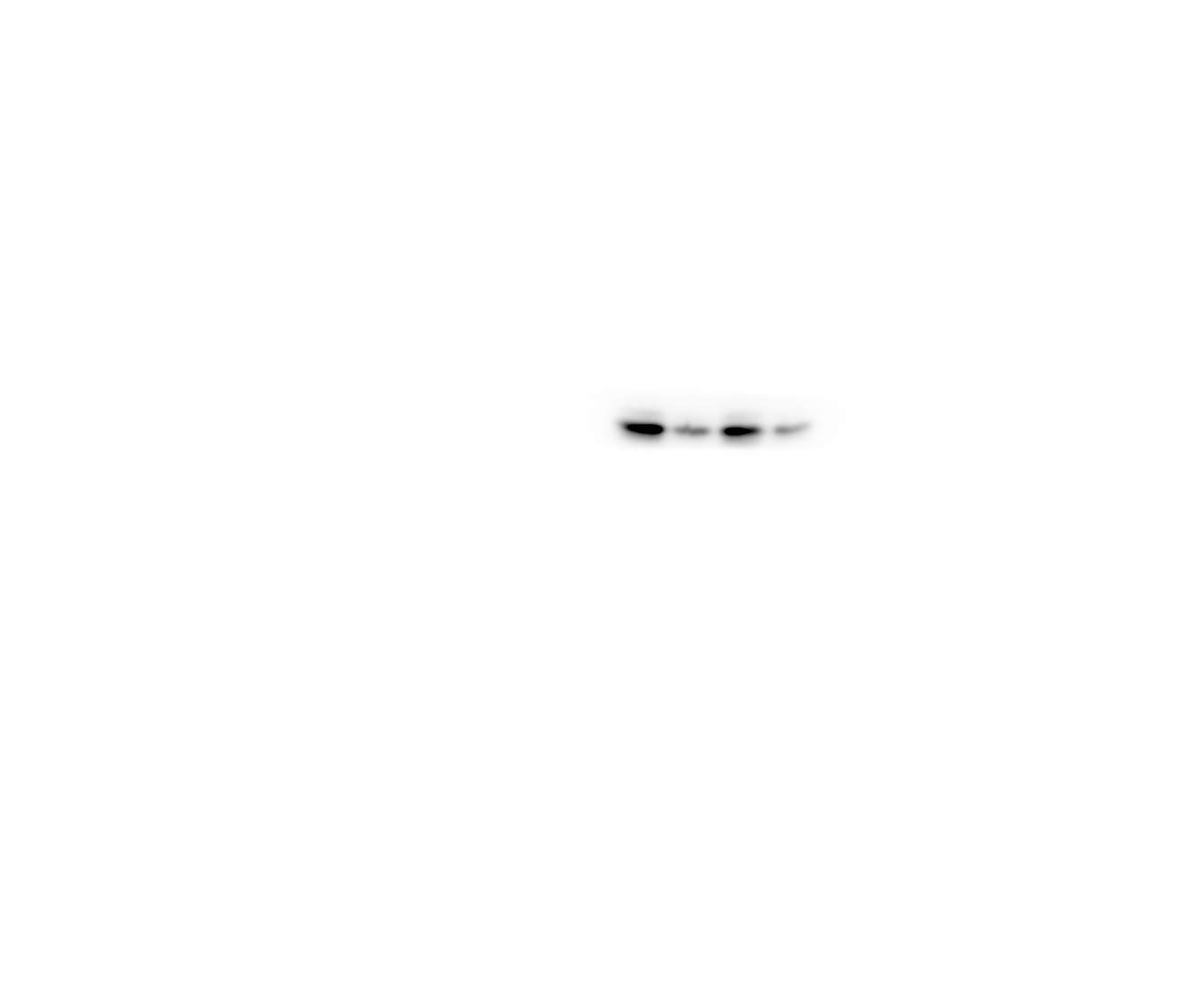

Supplement: Supplemental Information 2 [file peerj-11-15458-s002.zip › RawDataFig2/Fig2/Fig2B/CTHRC1 02.png]

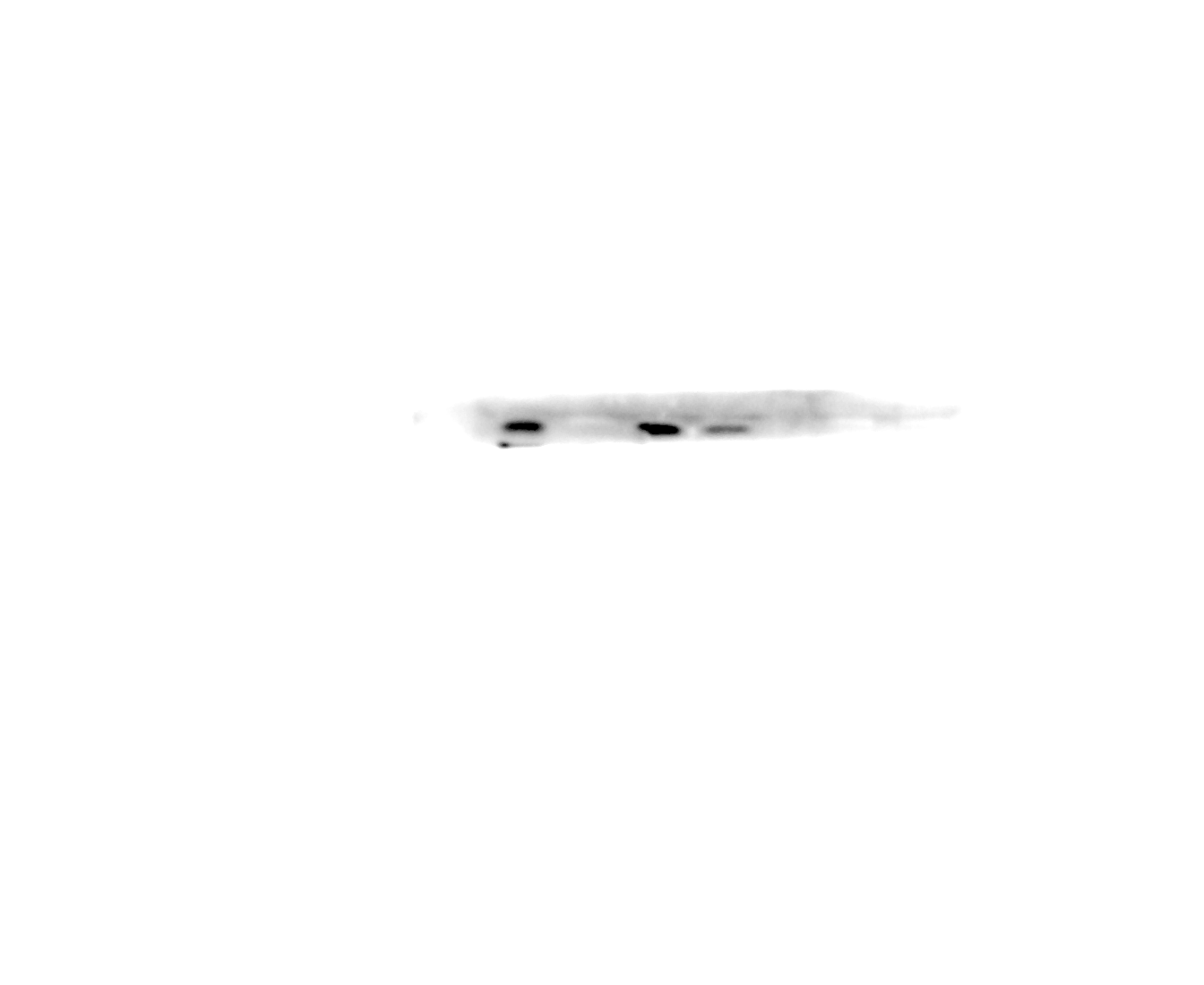

Supplement: Supplemental Information 2 [file peerj-11-15458-s002.zip › RawDataFig2/Fig2/Fig2B/CTHRC1 03.png]

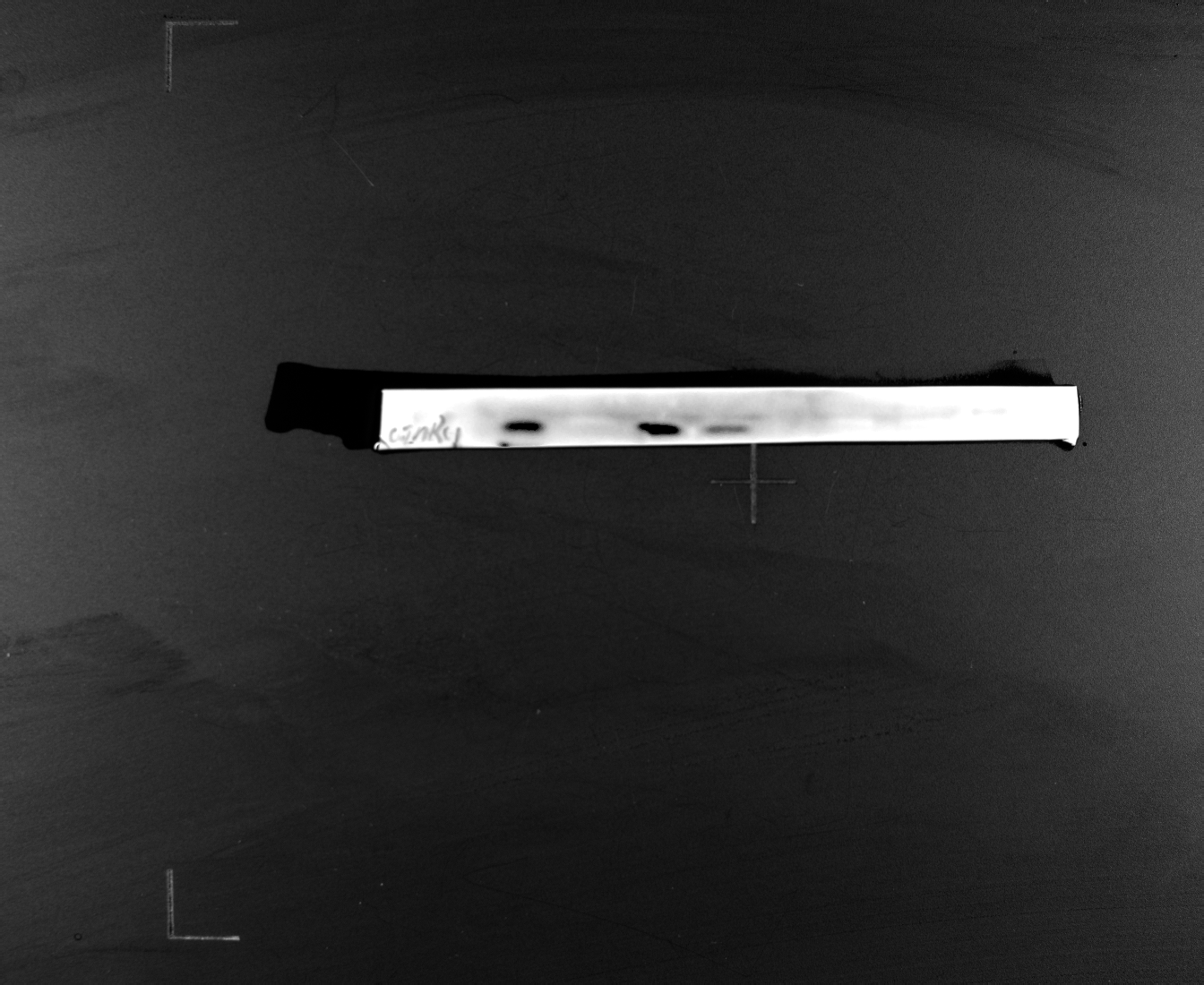

Supplement: Supplemental Information 2 [file peerj-11-15458-s002.zip › RawDataFig2/Fig2/Fig2B/CTHRC1 04.png]

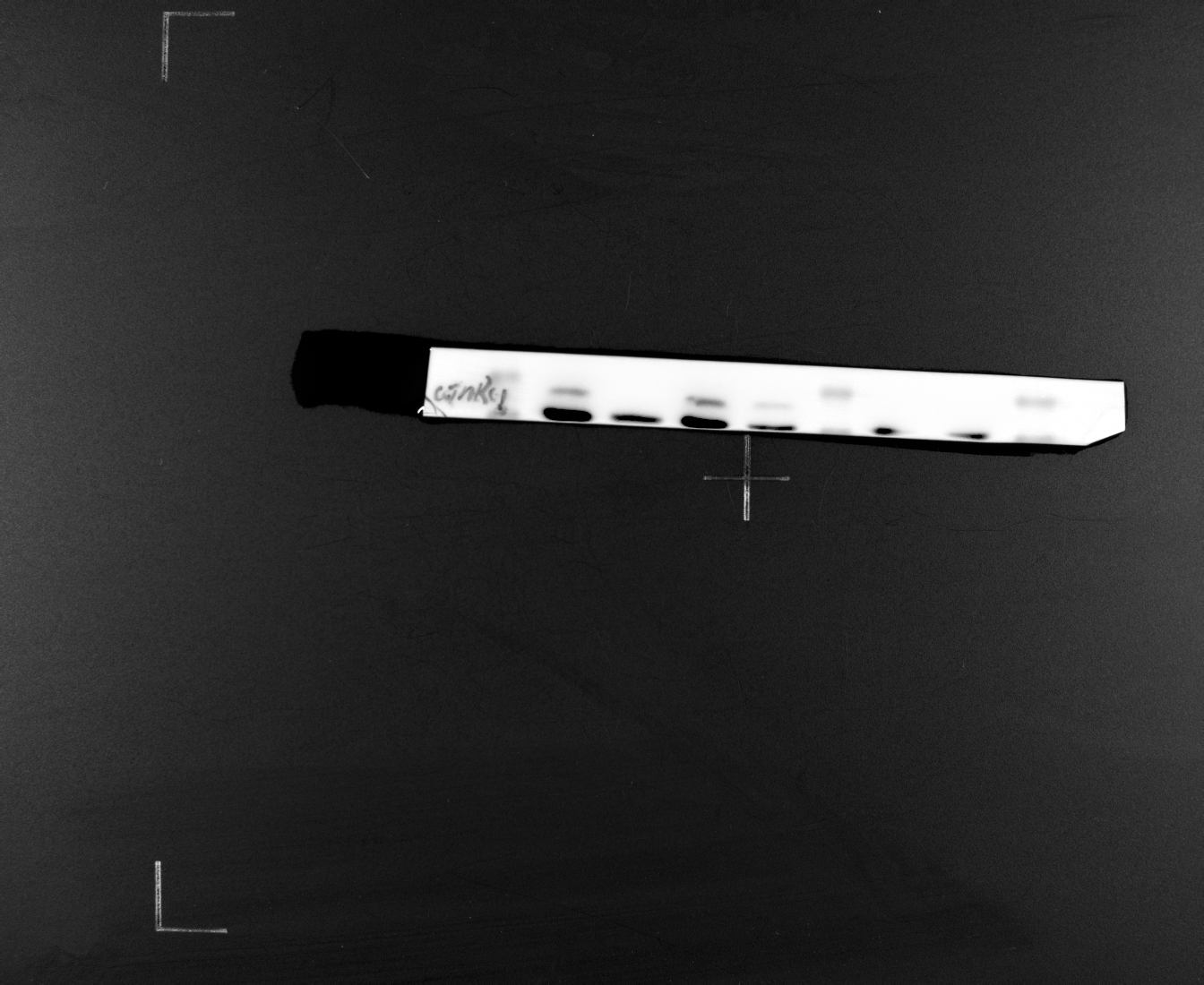

Supplement: Supplemental Information 2 [file peerj-11-15458-s002.zip › RawDataFig2/Fig2/Fig2B/CTHRC1 05.png]

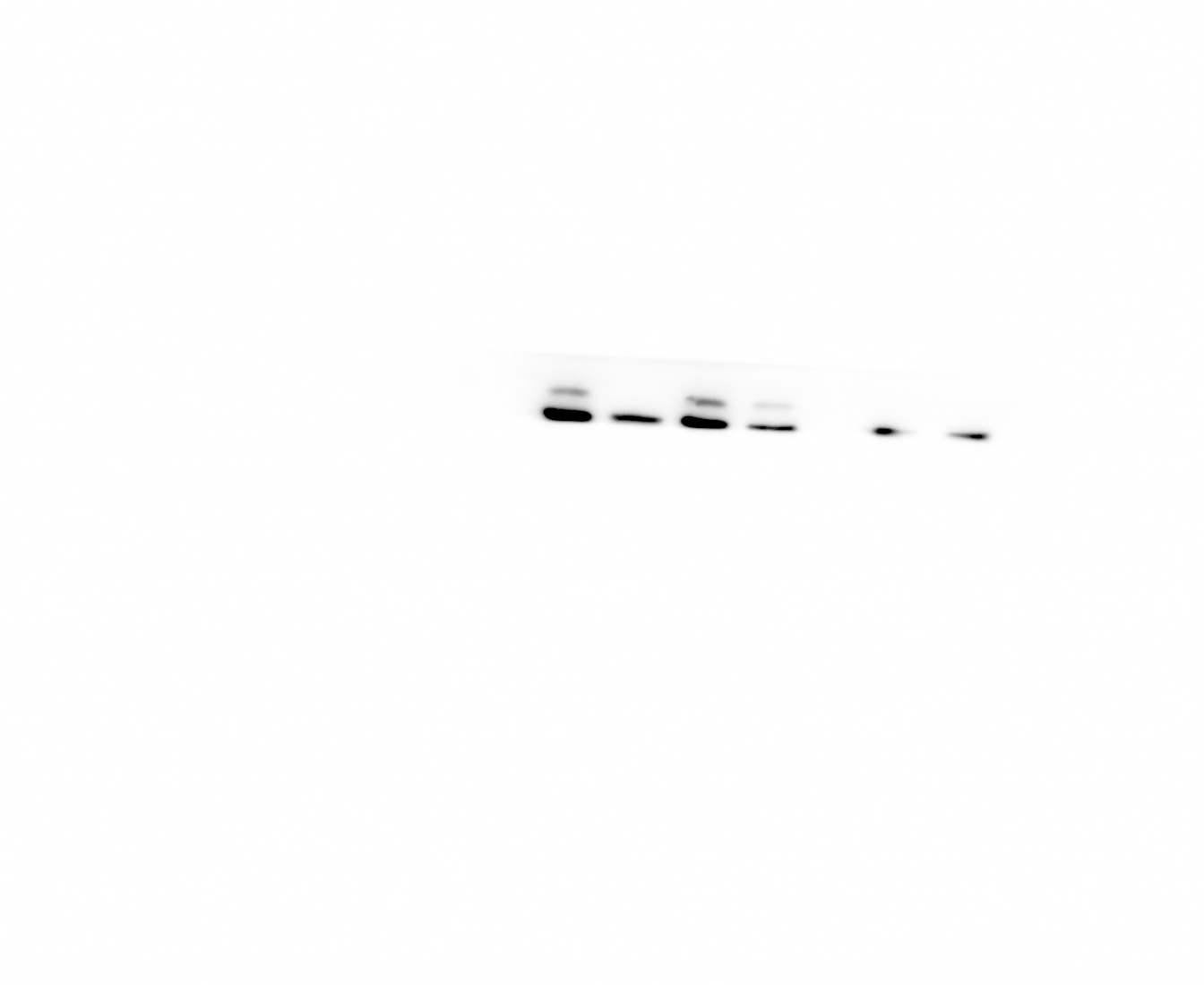

Supplement: Supplemental Information 2 [file peerj-11-15458-s002.zip › RawDataFig2/Fig2/Fig2B/CTHRC1 06.png]

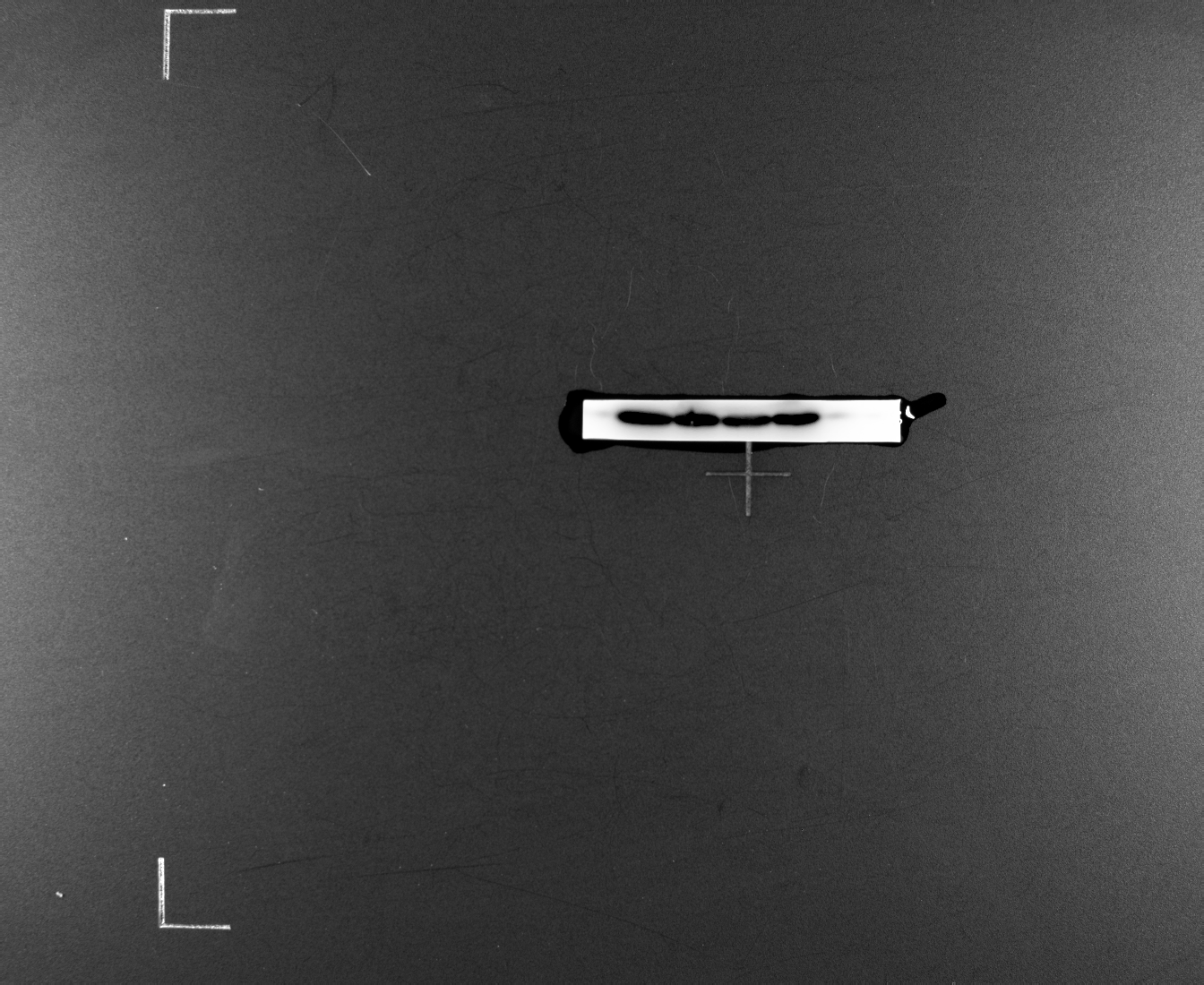

Supplement: Supplemental Information 2 [file peerj-11-15458-s002.zip › RawDataFig2/Fig2/Fig2B/GAPDH 01.png]

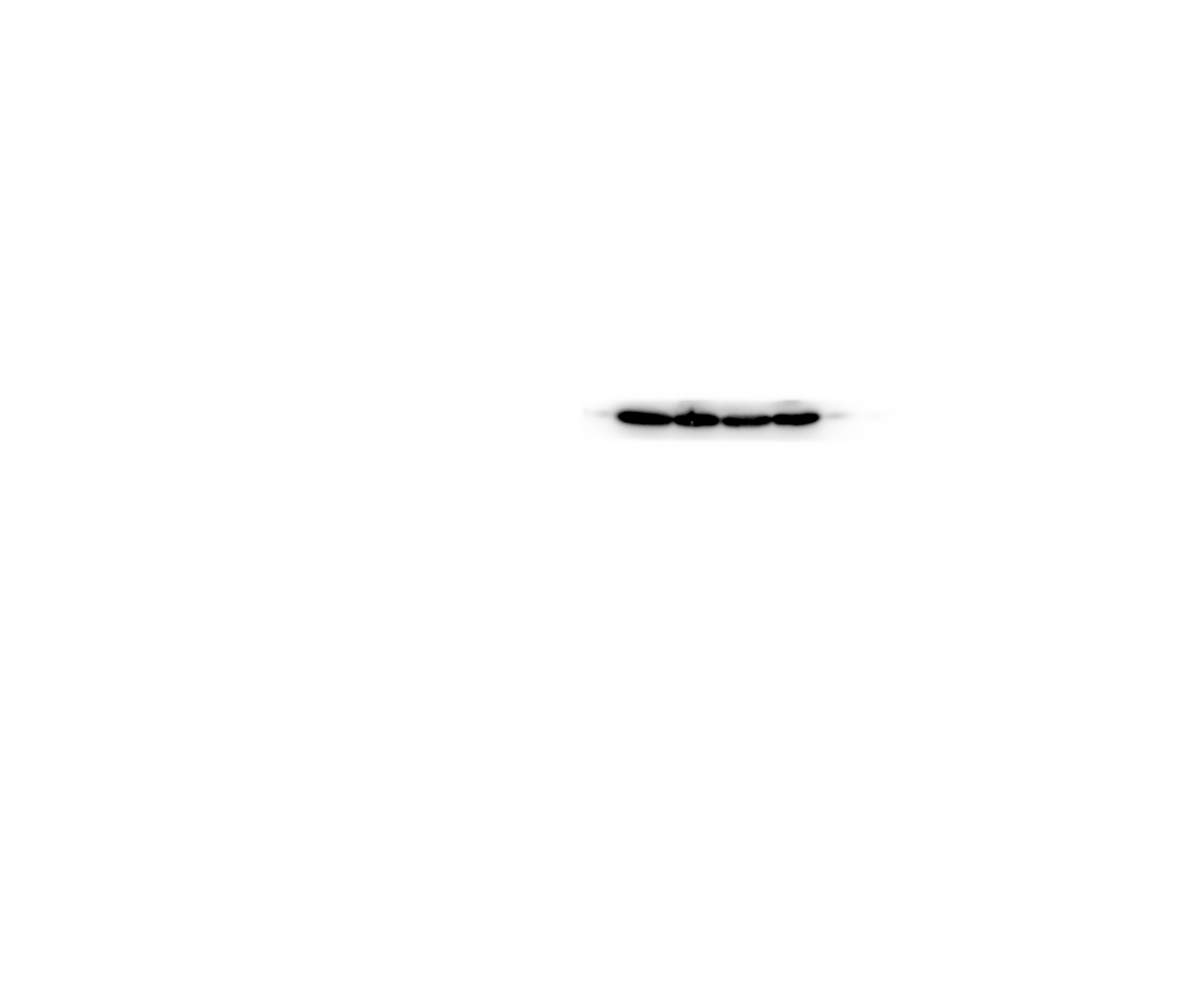

Supplement: Supplemental Information 2 [file peerj-11-15458-s002.zip › RawDataFig2/Fig2/Fig2B/GAPDH 02.png]

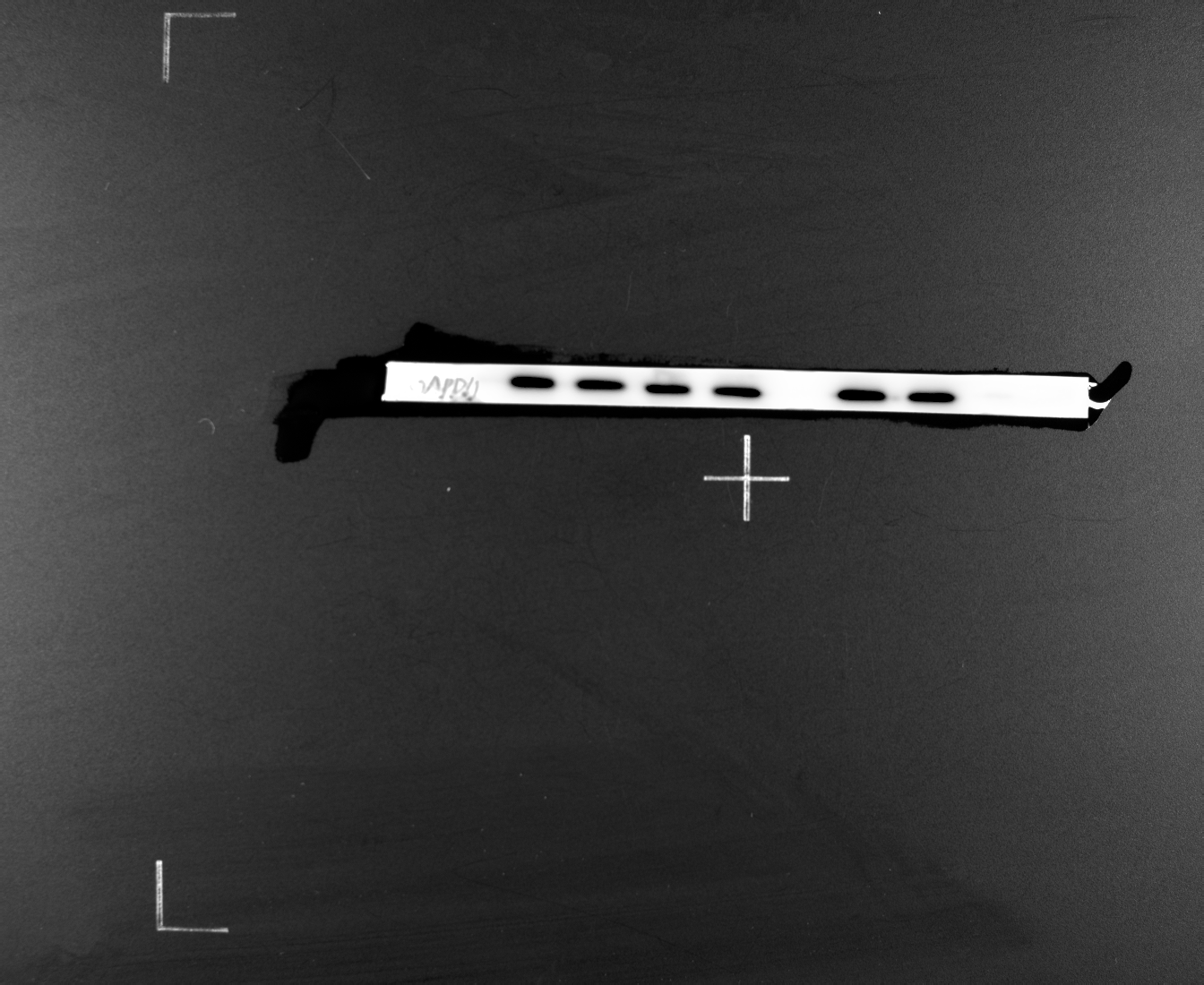

Supplement: Supplemental Information 2 [file peerj-11-15458-s002.zip › RawDataFig2/Fig2/Fig2B/GAPDH 03.png]

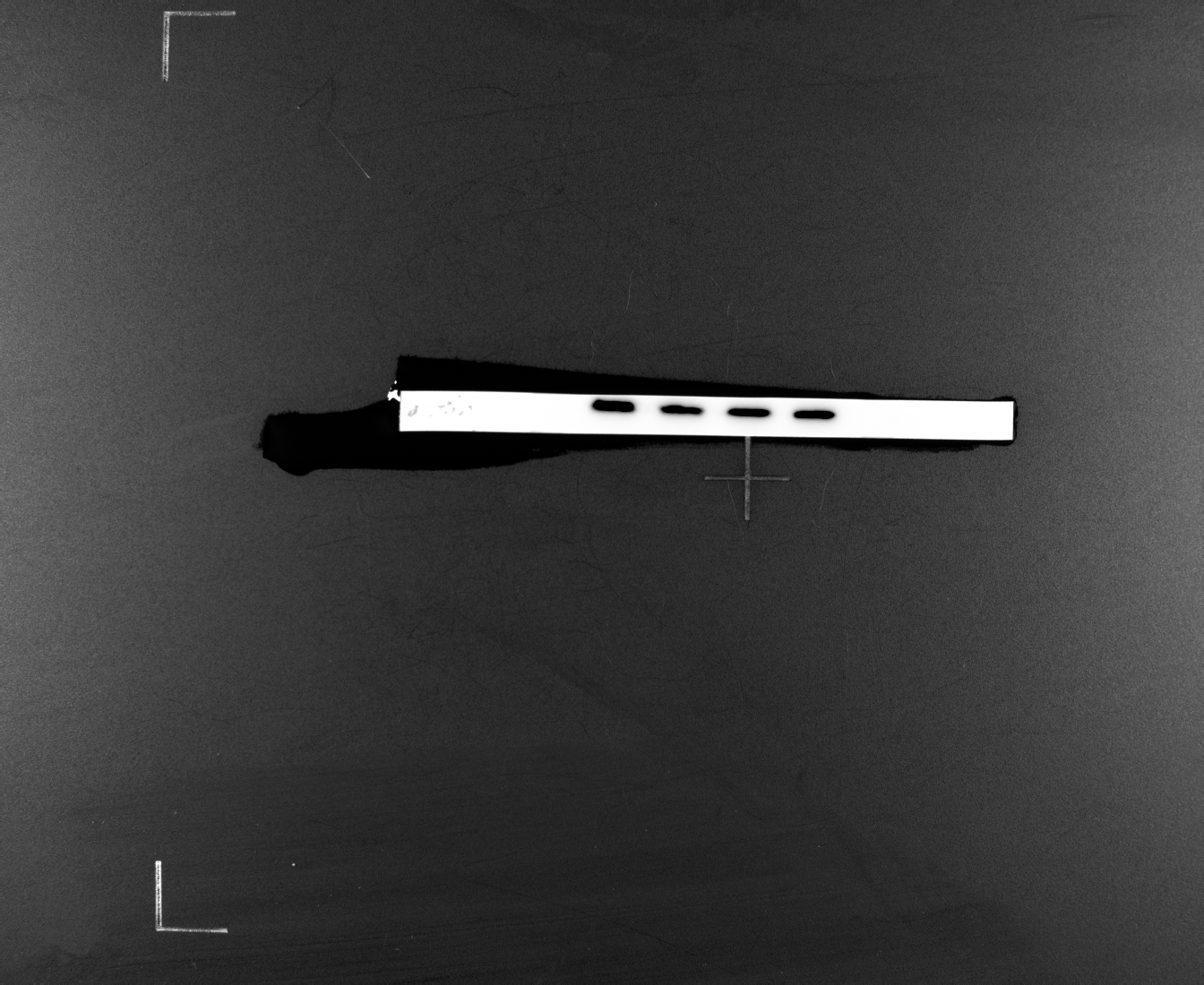

Supplement: Supplemental Information 2 [file peerj-11-15458-s002.zip › RawDataFig2/Fig2/Fig2B/GAPDH 05.png]

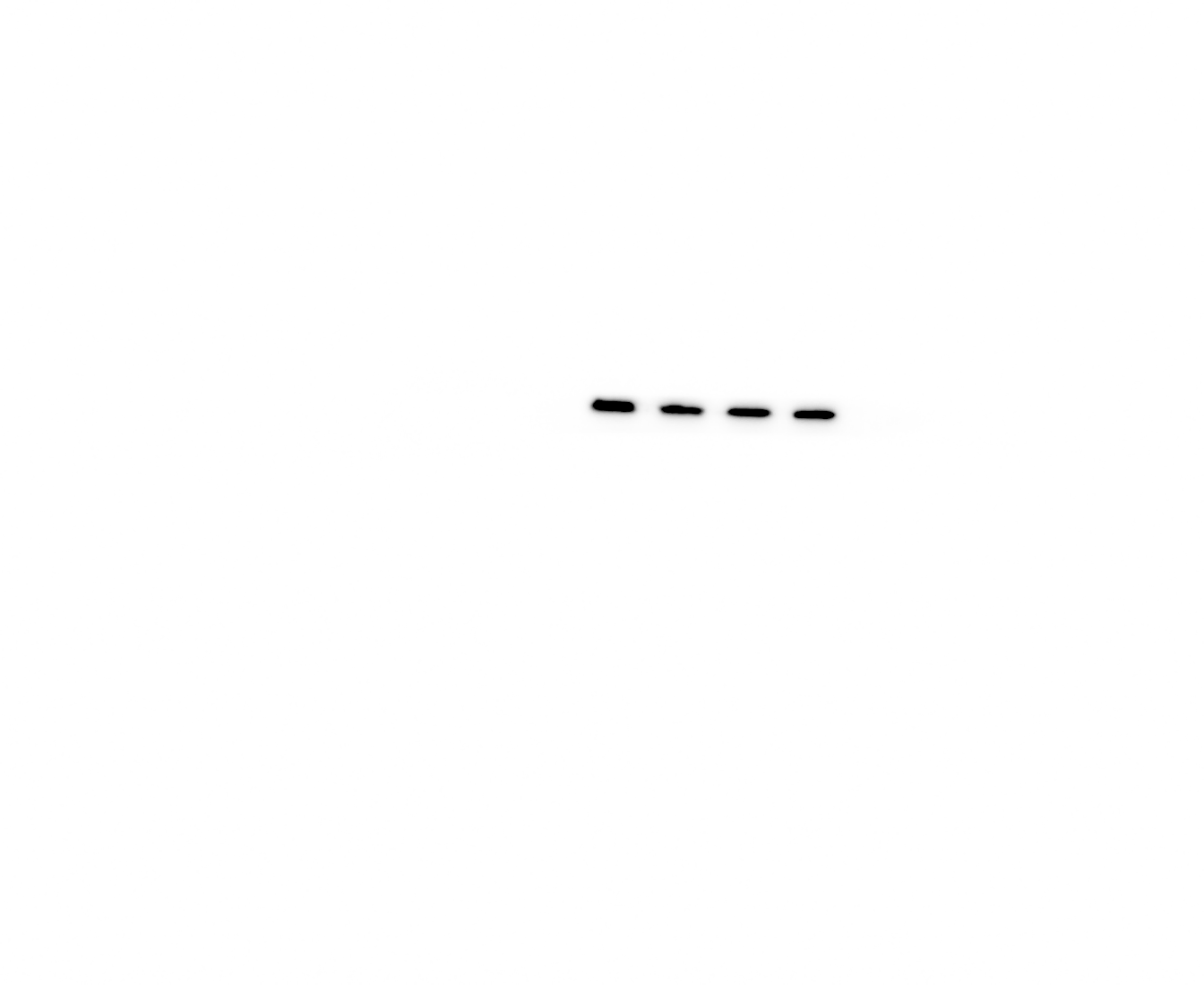

Supplement: Supplemental Information 2 [file peerj-11-15458-s002.zip › RawDataFig2/Fig2/Fig2B/GAPDH 06.png]

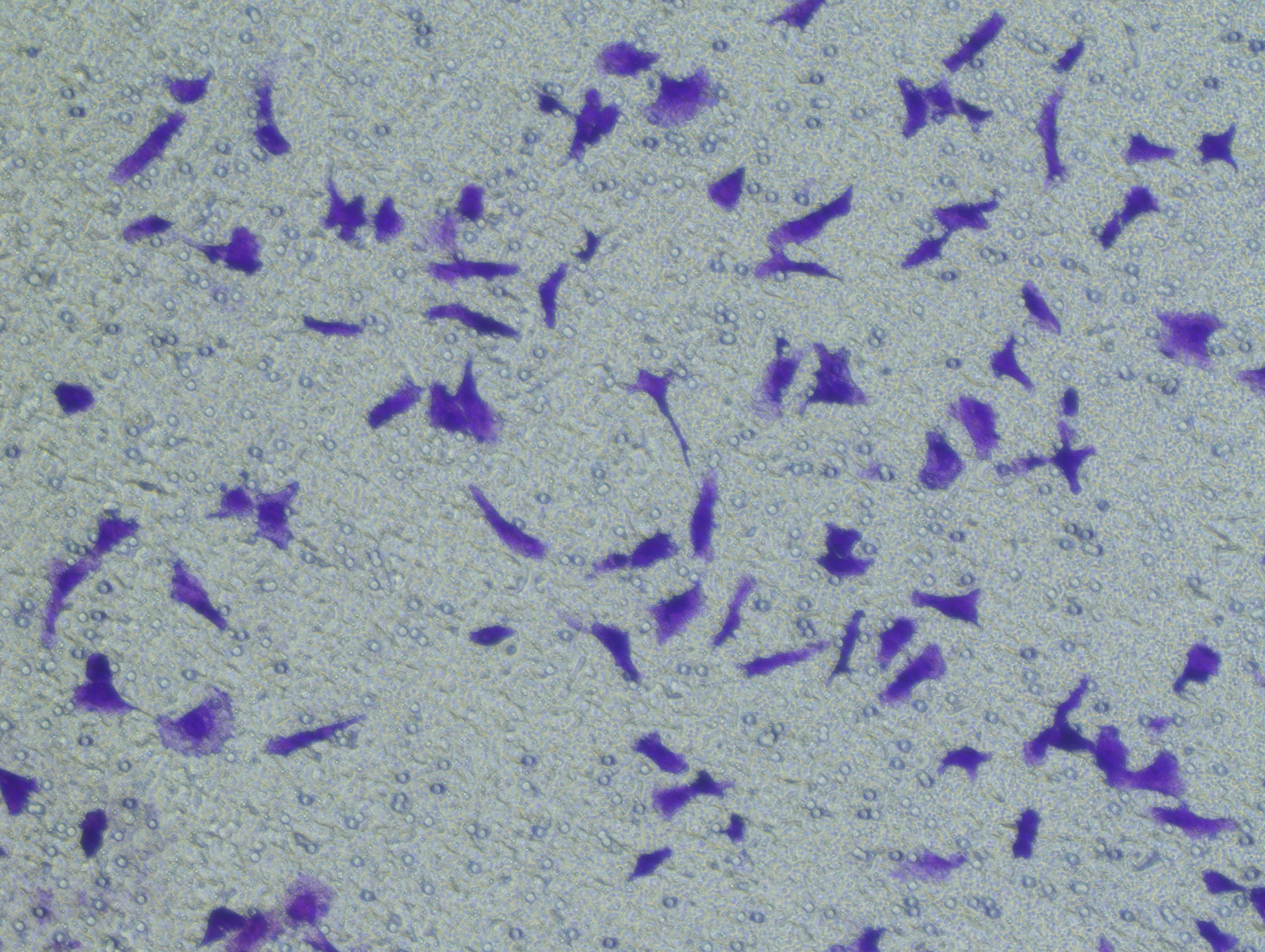

Supplement: Supplemental Information 2 [file peerj-11-15458-s002.zip › RawDataFig2/Fig2/Fig2C/invasion/T238 NC 01.jpg]

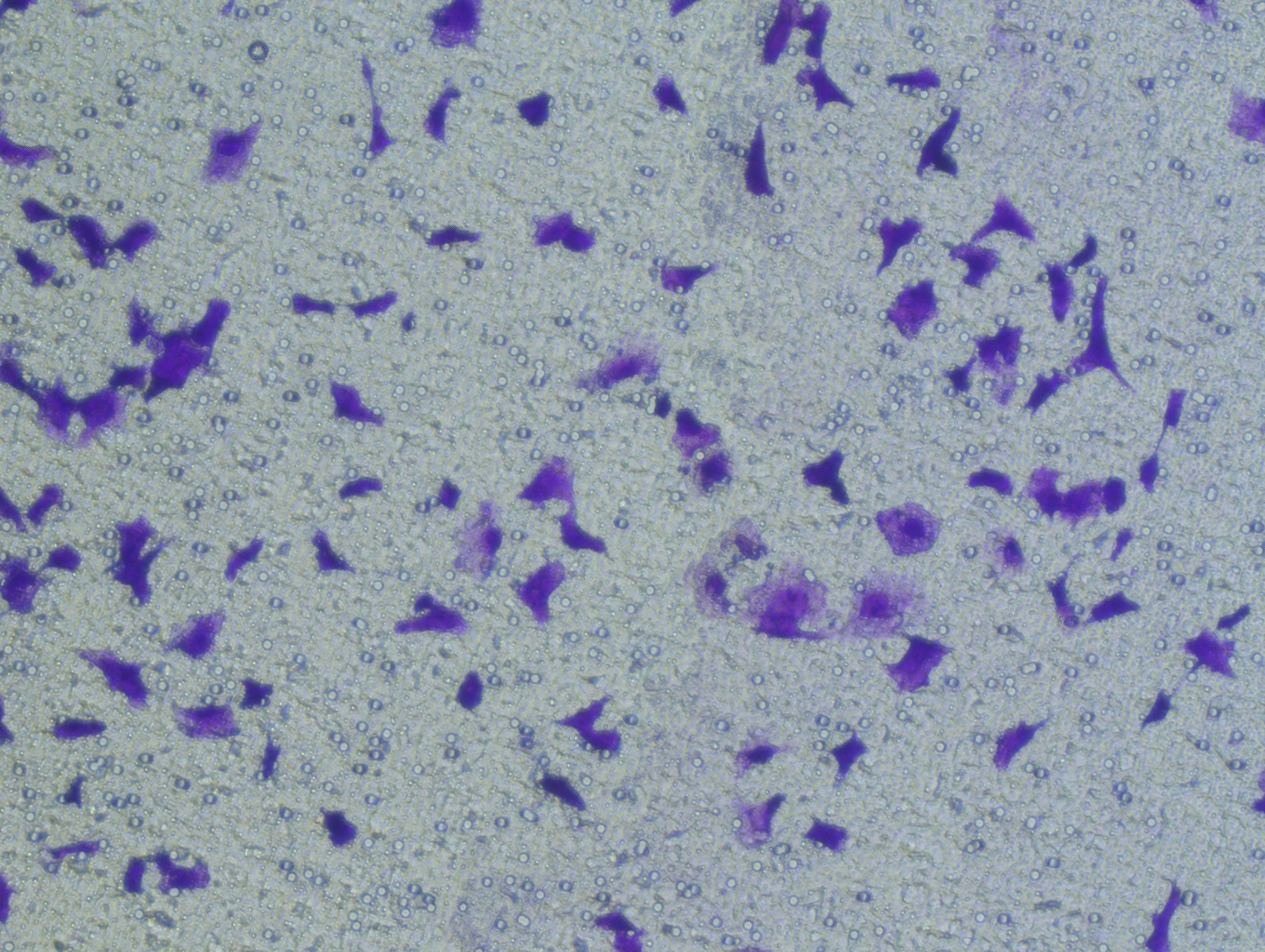

Supplement: Supplemental Information 2 [file peerj-11-15458-s002.zip › RawDataFig2/Fig2/Fig2C/invasion/T238 NC 02.jpg]

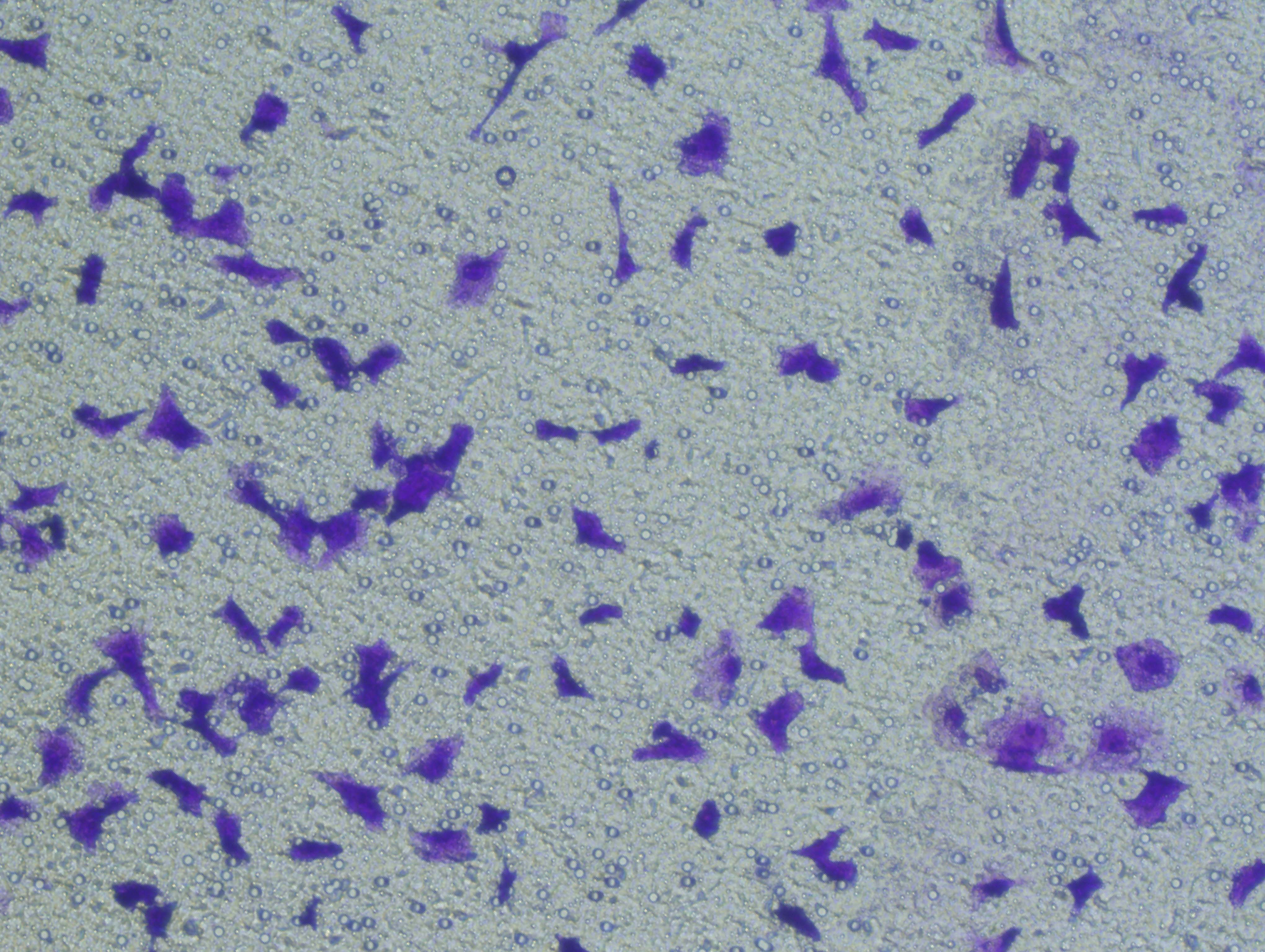

Supplement: Supplemental Information 2 [file peerj-11-15458-s002.zip › RawDataFig2/Fig2/Fig2C/invasion/T238 NC 03.jpg]

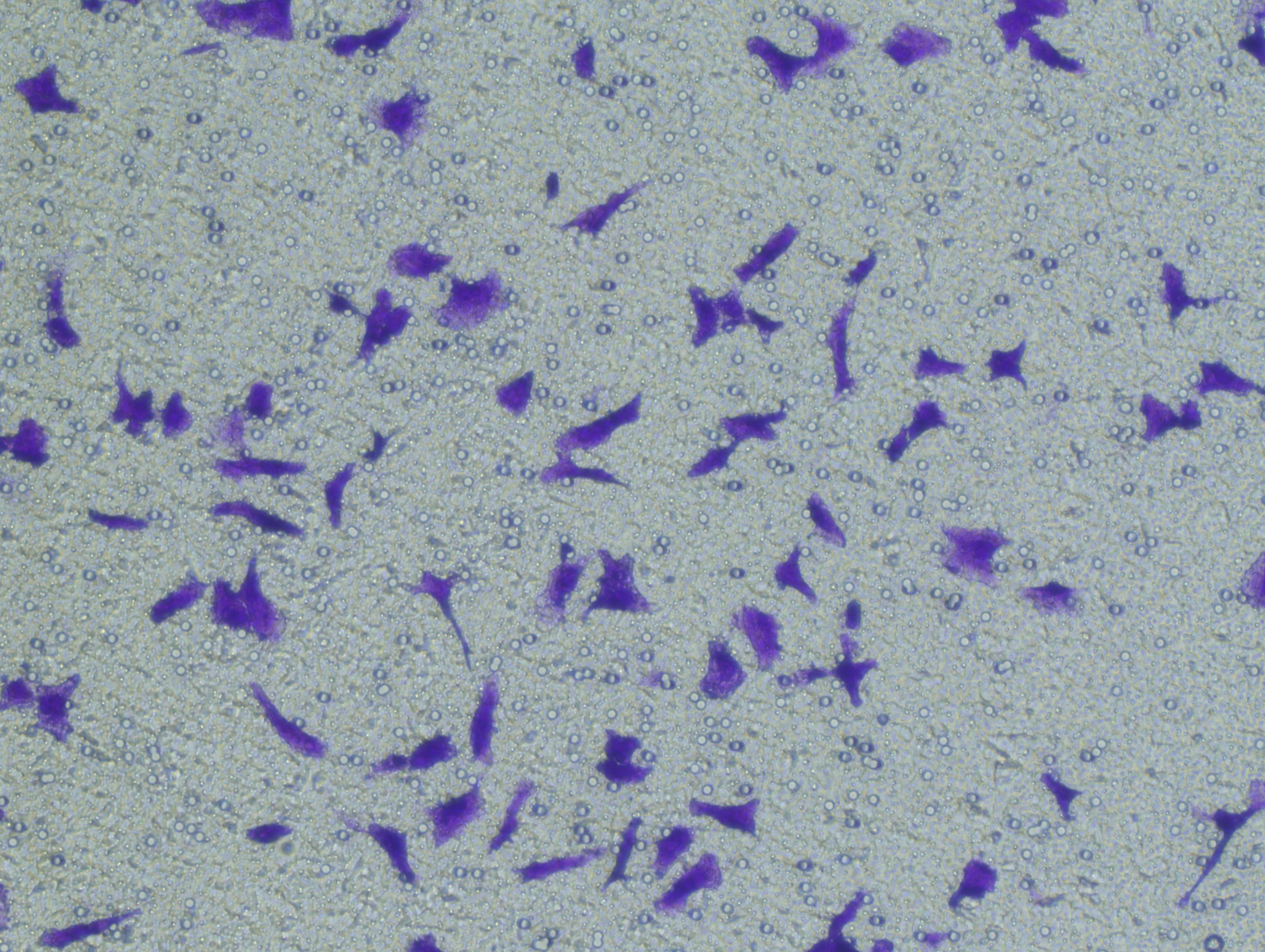

Supplement: Supplemental Information 2 [file peerj-11-15458-s002.zip › RawDataFig2/Fig2/Fig2C/invasion/T238 NC 04.jpg]

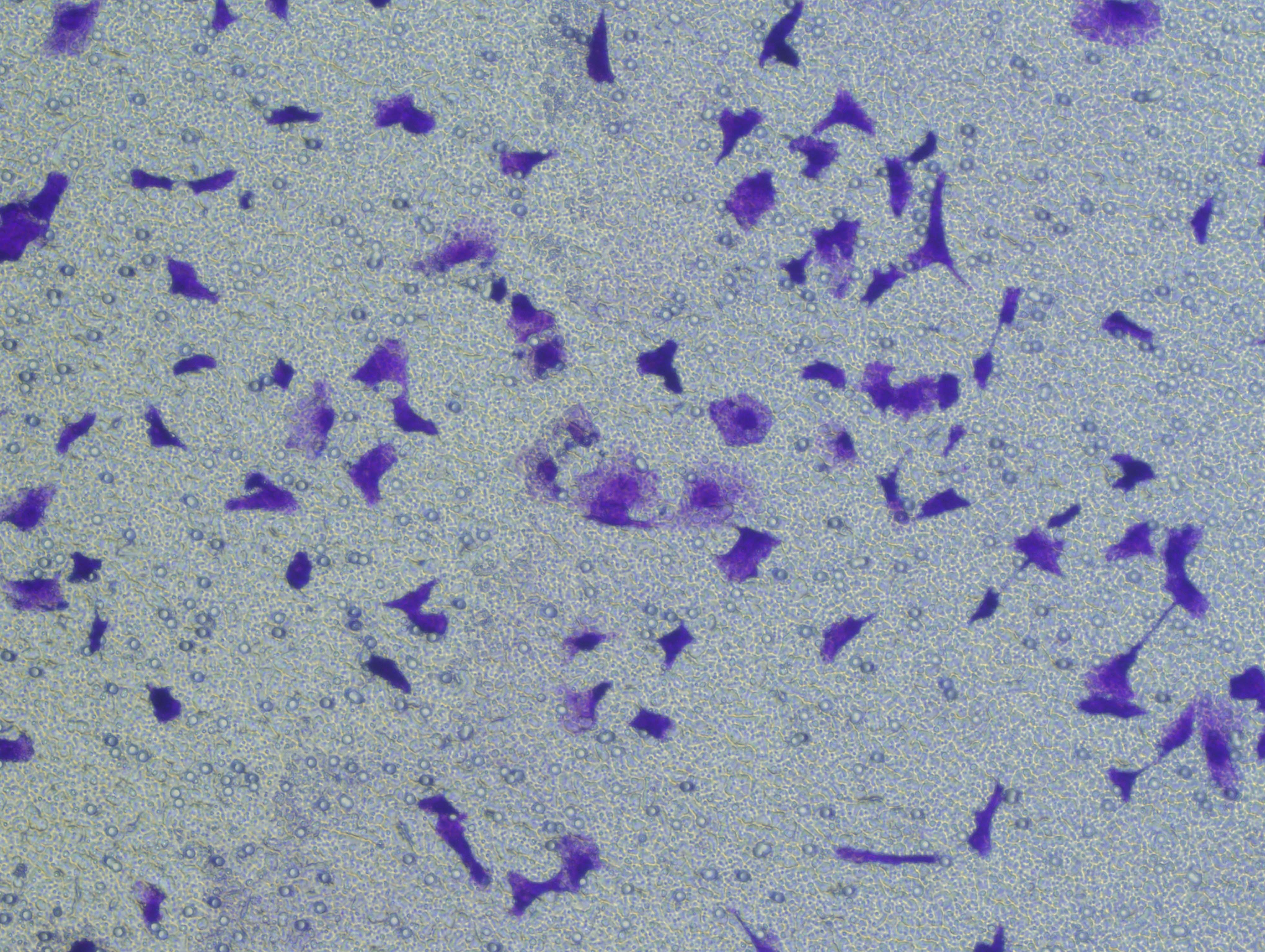

Supplement: Supplemental Information 2 [file peerj-11-15458-s002.zip › RawDataFig2/Fig2/Fig2C/invasion/T238 NC 05.jpg]

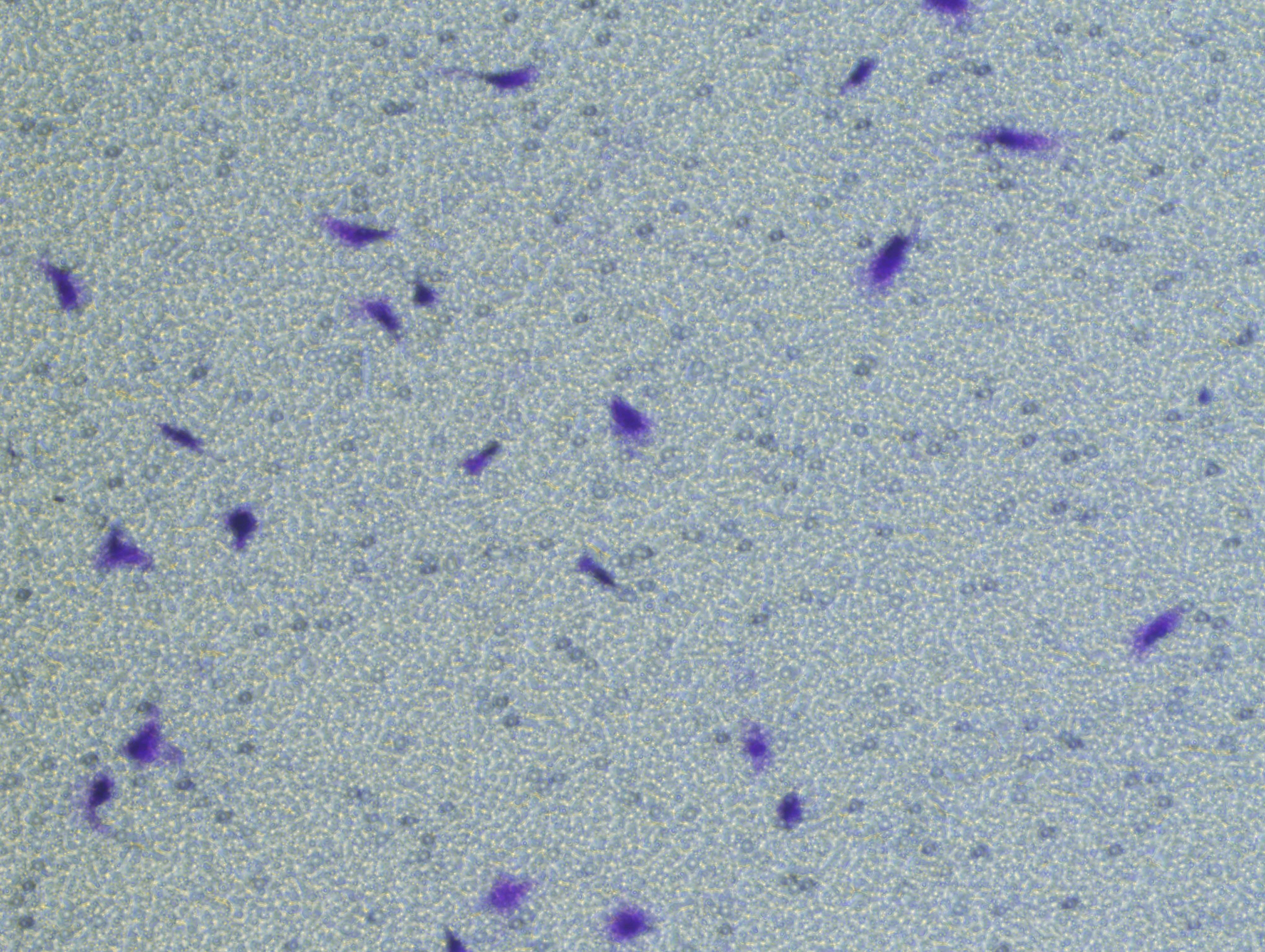

Supplement: Supplemental Information 2 [file peerj-11-15458-s002.zip › RawDataFig2/Fig2/Fig2C/invasion/T238 sh-CTHRC1 01.jpg]

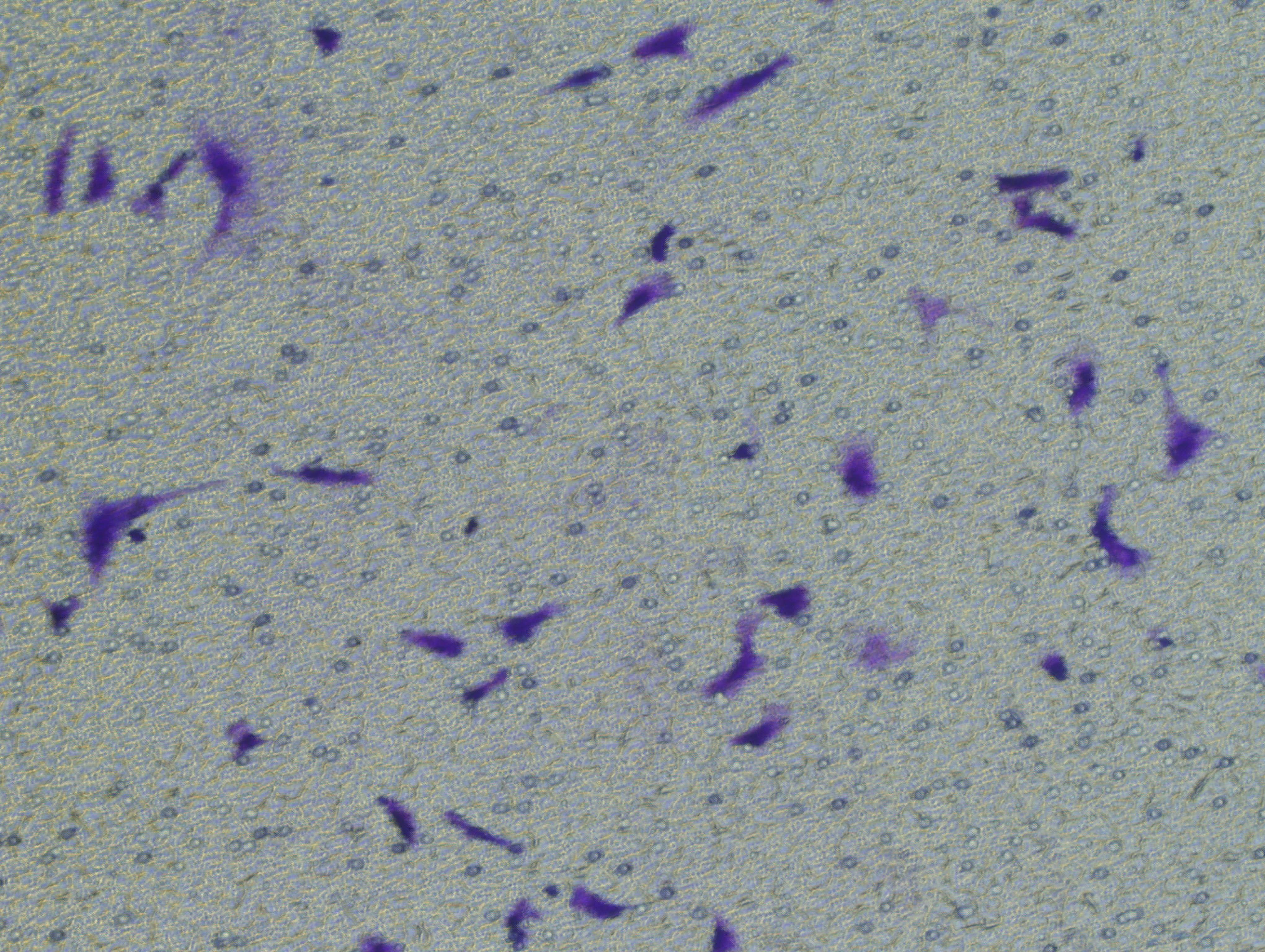

Supplement: Supplemental Information 2 [file peerj-11-15458-s002.zip › RawDataFig2/Fig2/Fig2C/invasion/T238 sh-CTHRC1 02.jpg]

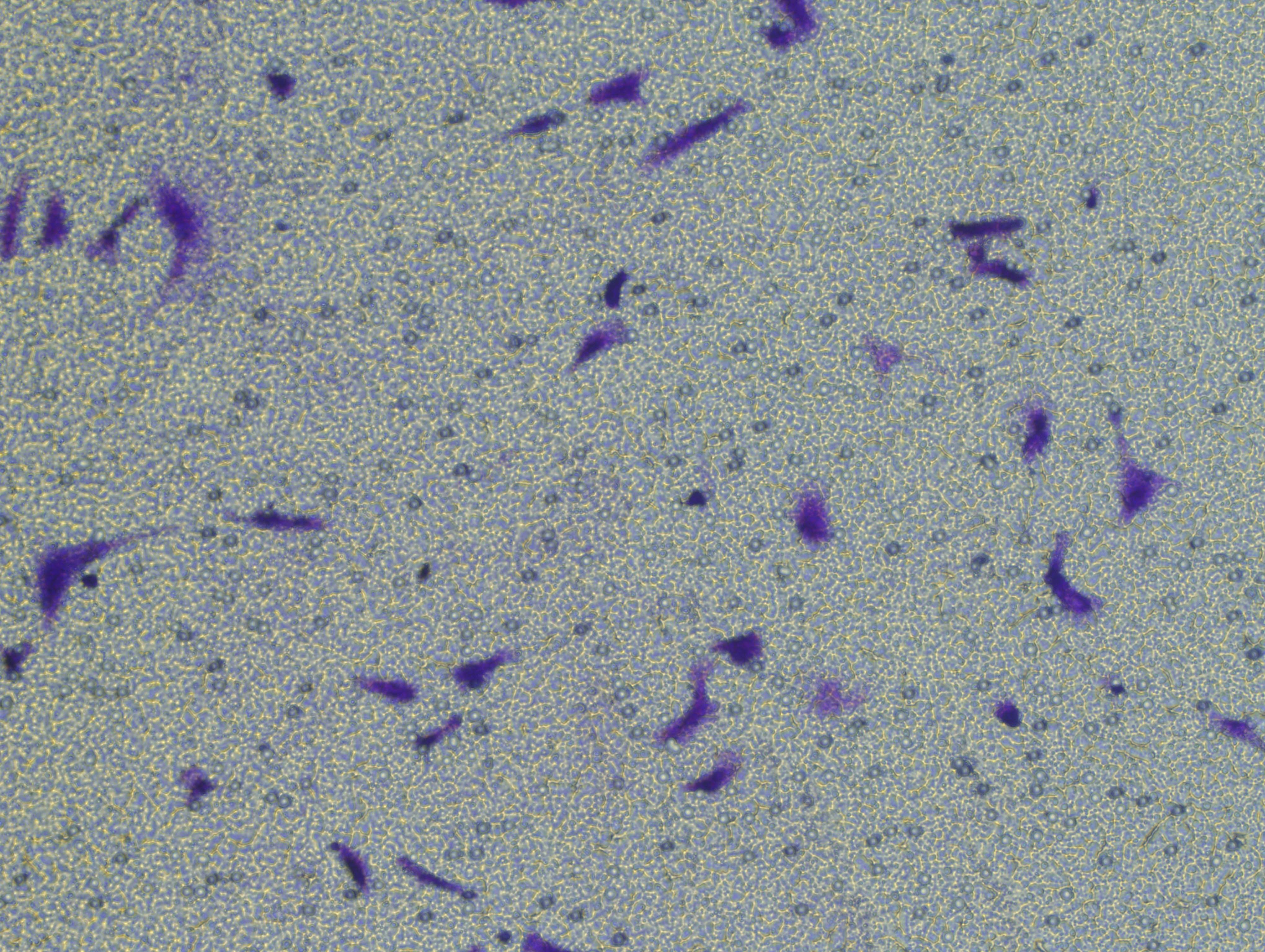

Supplement: Supplemental Information 2 [file peerj-11-15458-s002.zip › RawDataFig2/Fig2/Fig2C/invasion/T238 sh-CTHRC1 03.jpg]

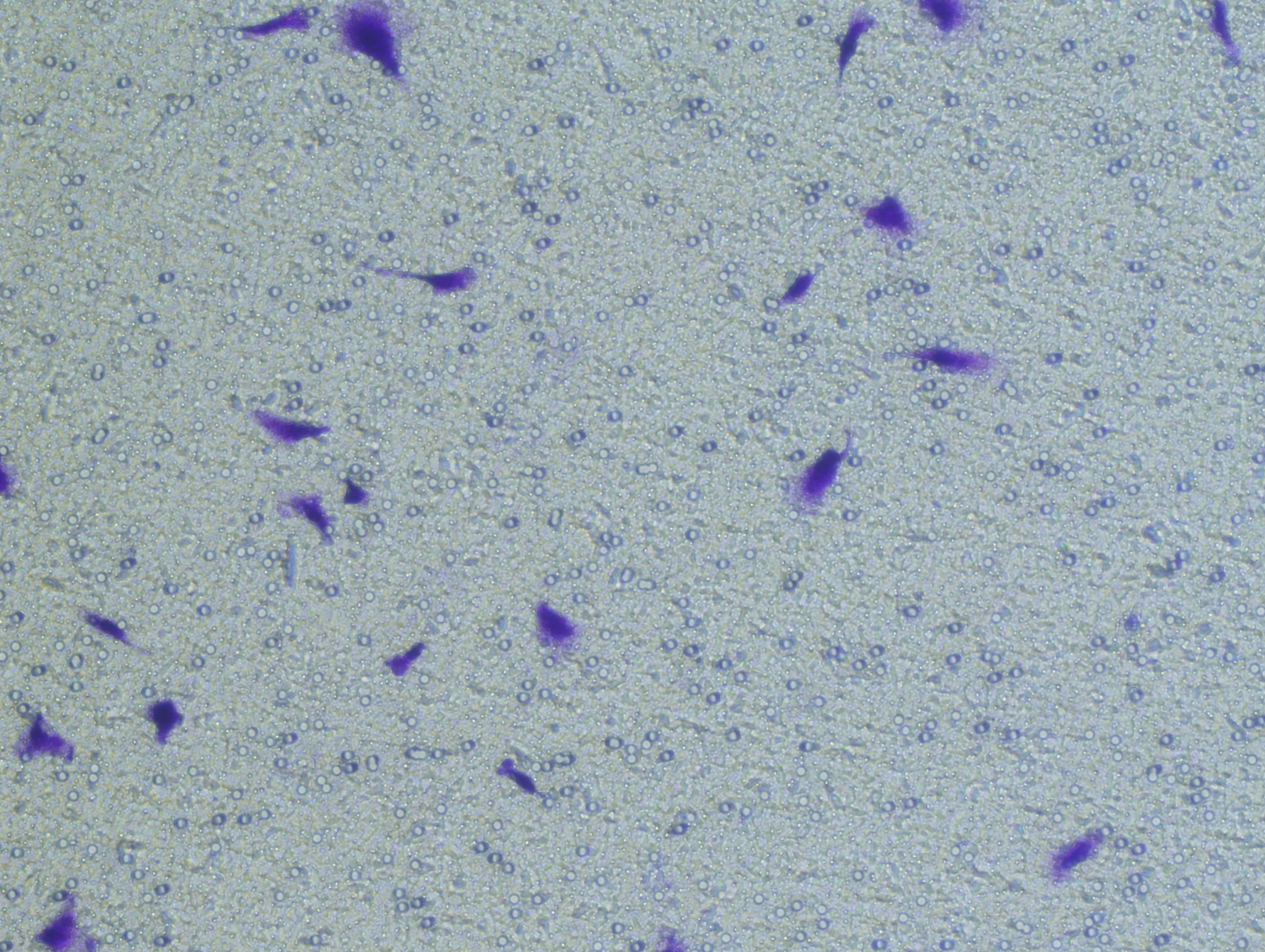

Supplement: Supplemental Information 2 [file peerj-11-15458-s002.zip › RawDataFig2/Fig2/Fig2C/invasion/T238 sh-CTHRC1 04.jpg]

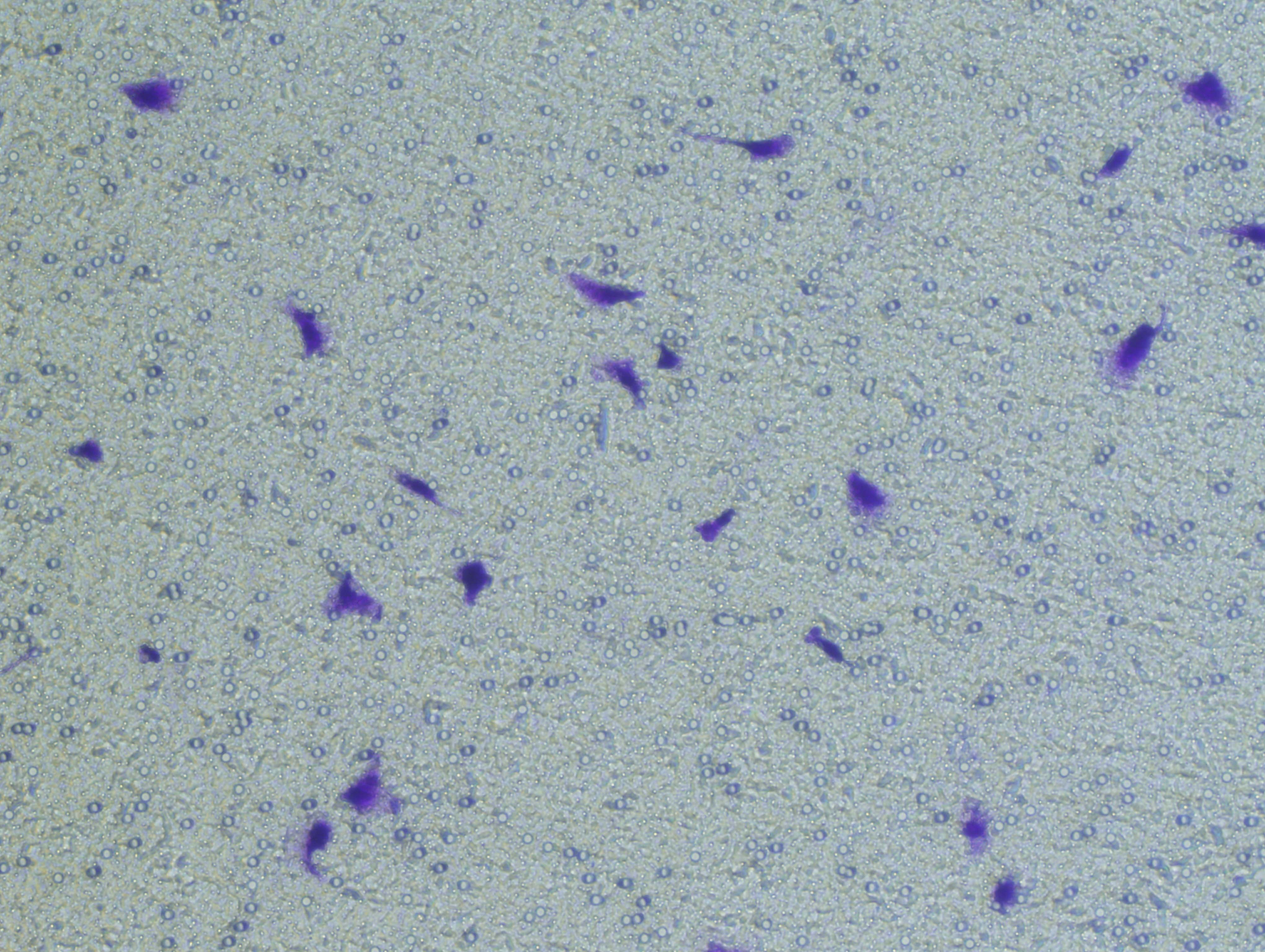

Supplement: Supplemental Information 2 [file peerj-11-15458-s002.zip › RawDataFig2/Fig2/Fig2C/invasion/T238 sh-CTHRC1 05.jpg]

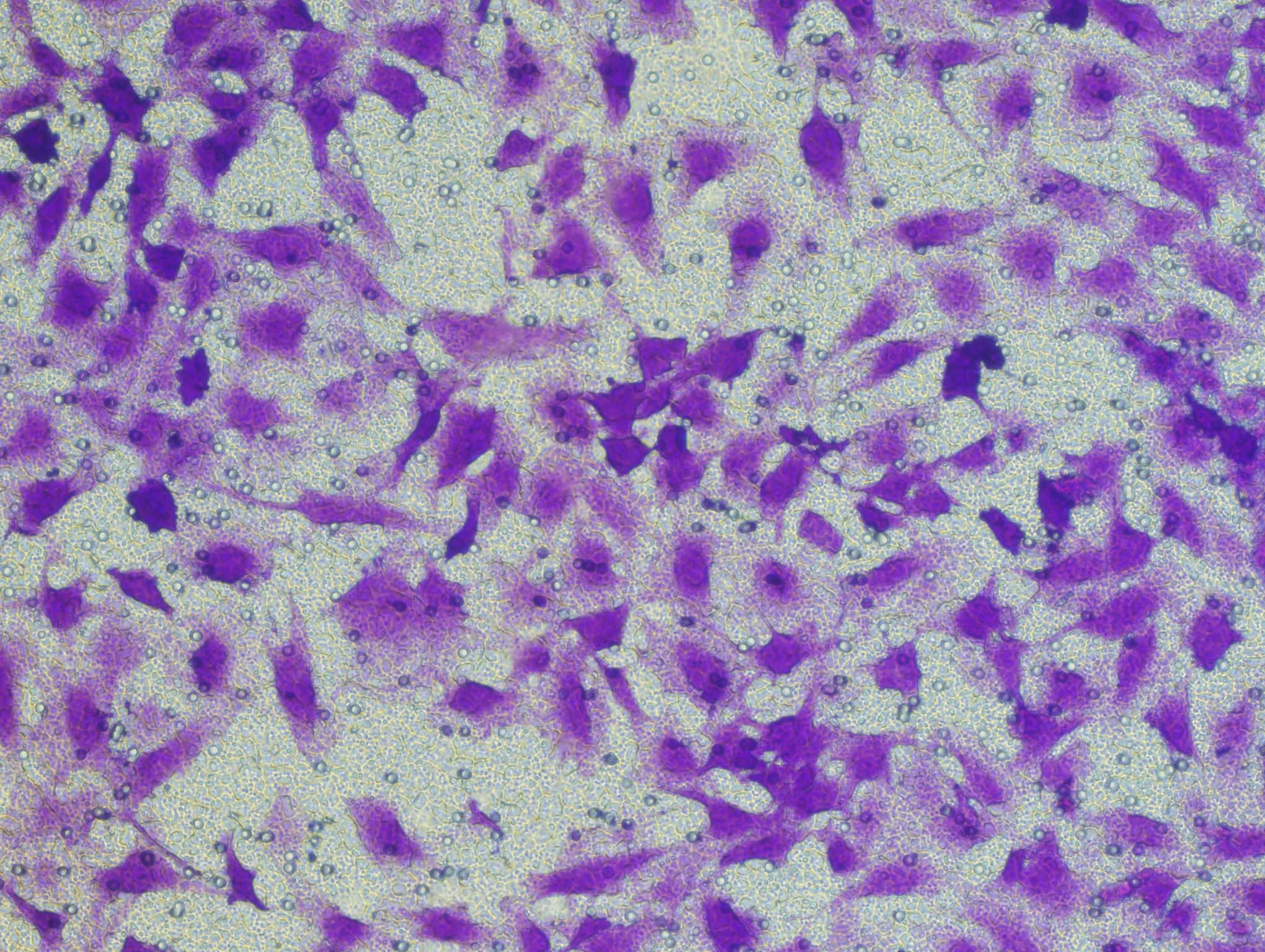

Supplement: Supplemental Information 2 [file peerj-11-15458-s002.zip › RawDataFig2/Fig2/Fig2C/invasion/THJ-16T NC 01.jpg]

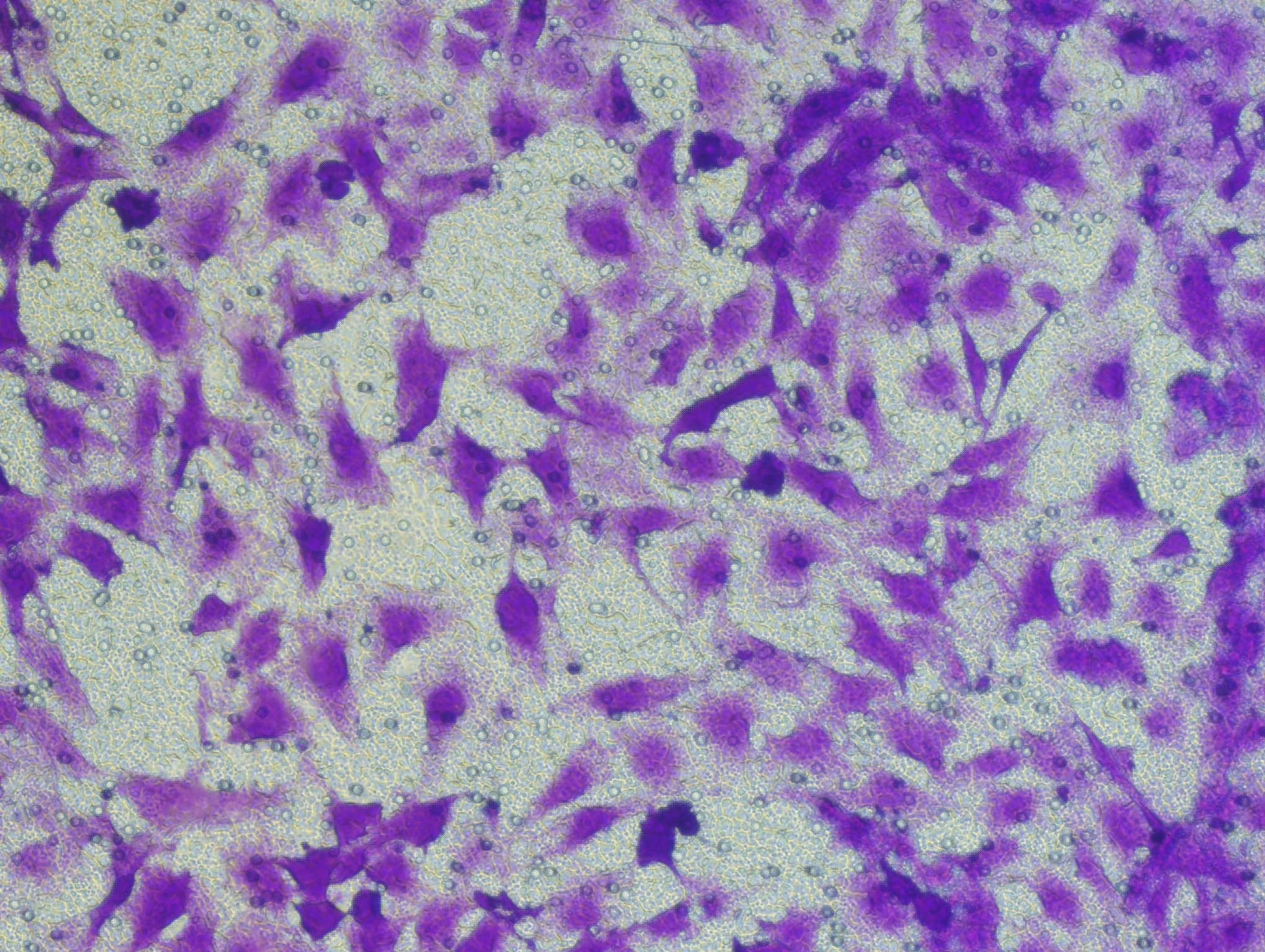

Supplement: Supplemental Information 2 [file peerj-11-15458-s002.zip › RawDataFig2/Fig2/Fig2C/invasion/THJ-16T NC 02.jpg]

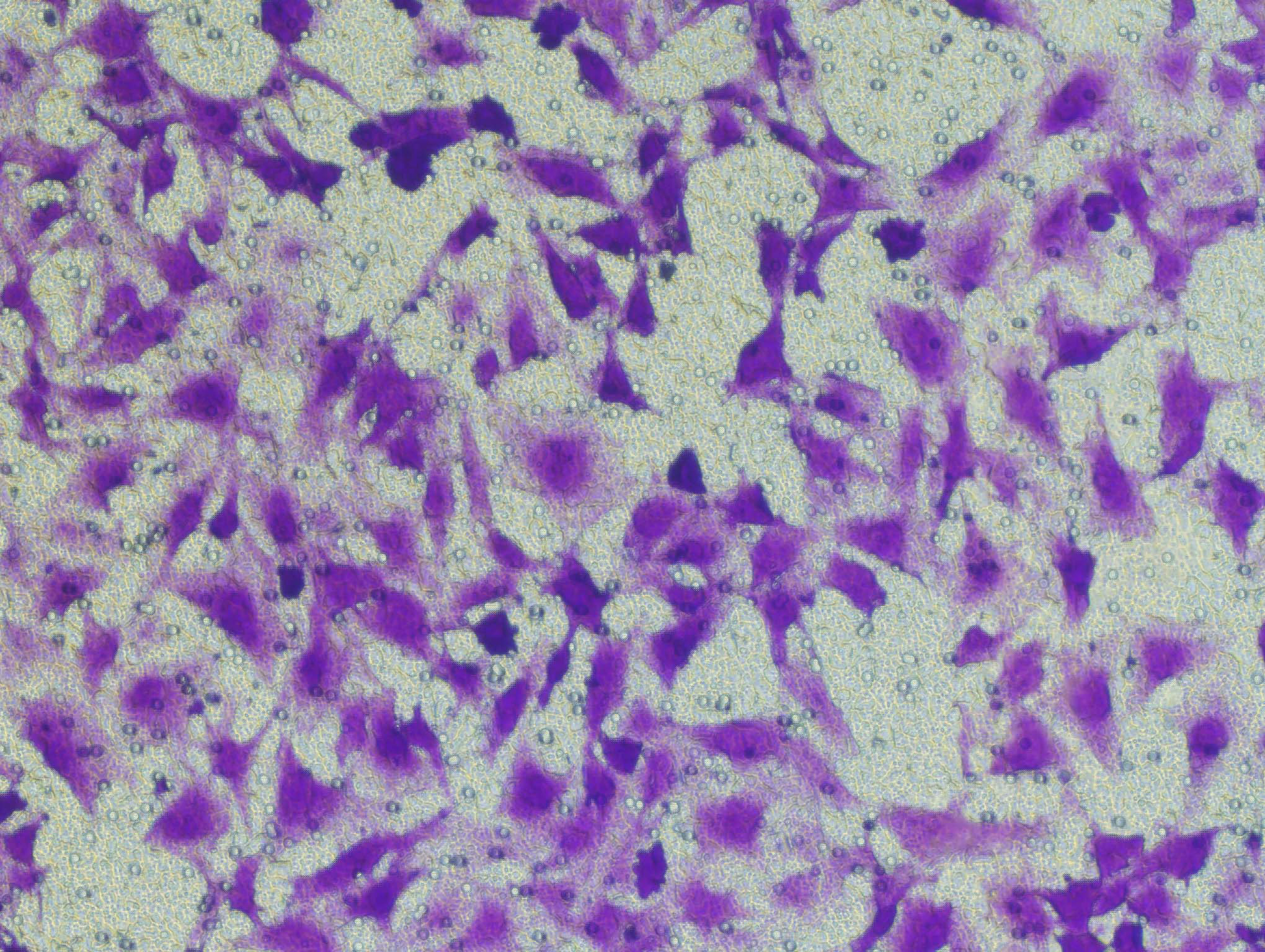

Supplement: Supplemental Information 2 [file peerj-11-15458-s002.zip › RawDataFig2/Fig2/Fig2C/invasion/THJ-16T NC 03.jpg]

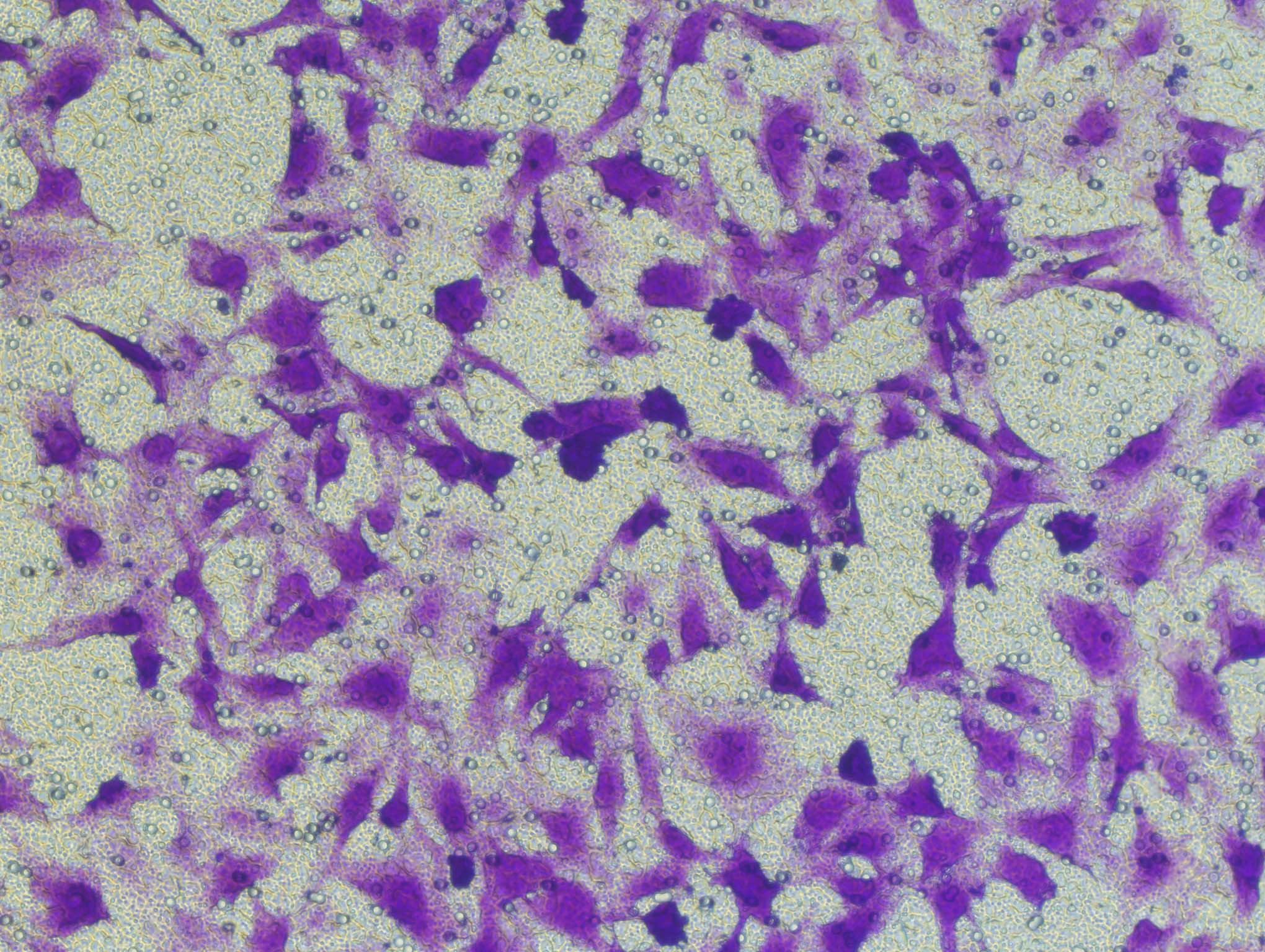

Supplement: Supplemental Information 2 [file peerj-11-15458-s002.zip › RawDataFig2/Fig2/Fig2C/invasion/THJ-16T NC 04.jpg]

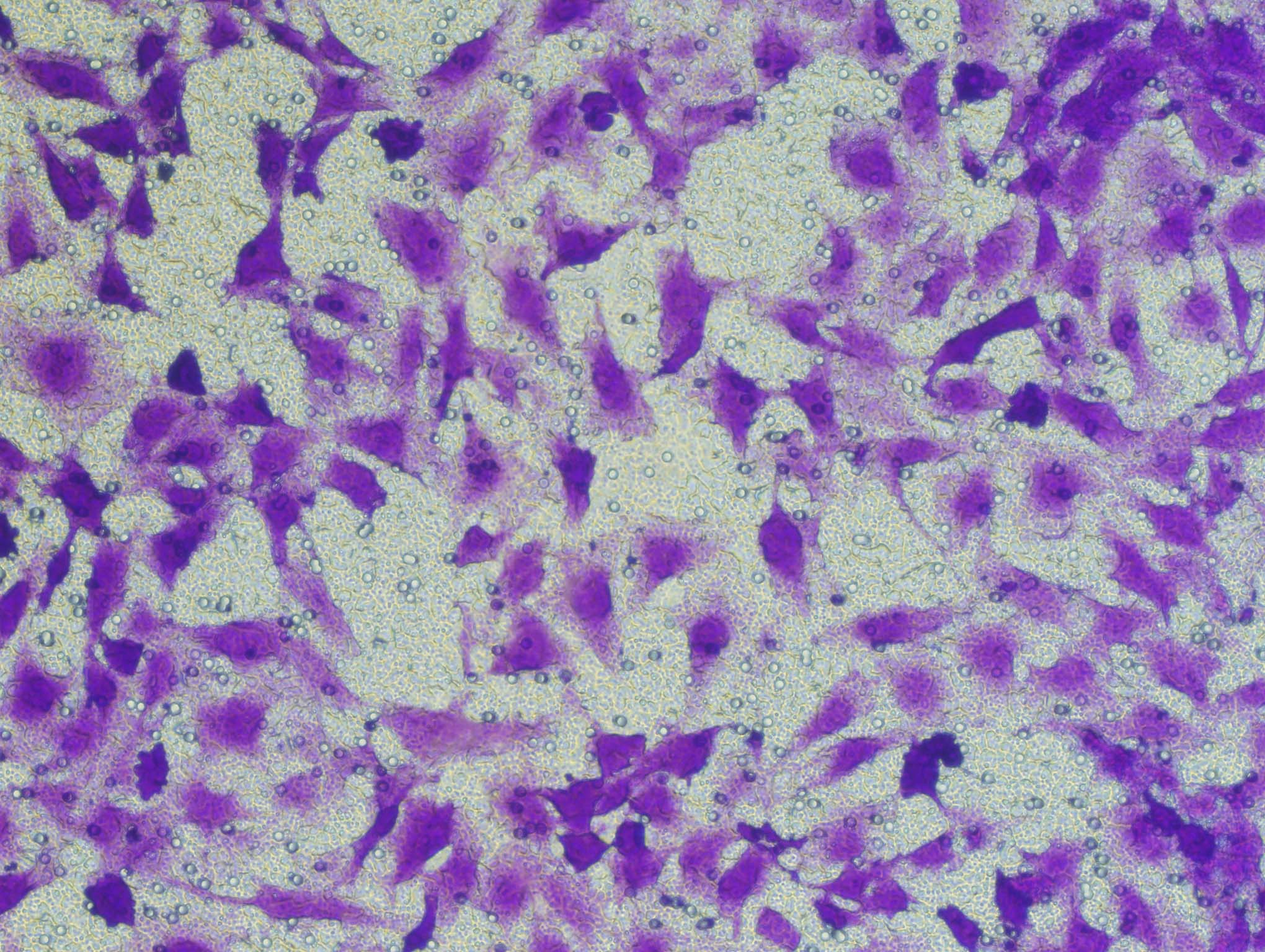

Supplement: Supplemental Information 2 [file peerj-11-15458-s002.zip › RawDataFig2/Fig2/Fig2C/invasion/THJ-16T NC 05.jpg]

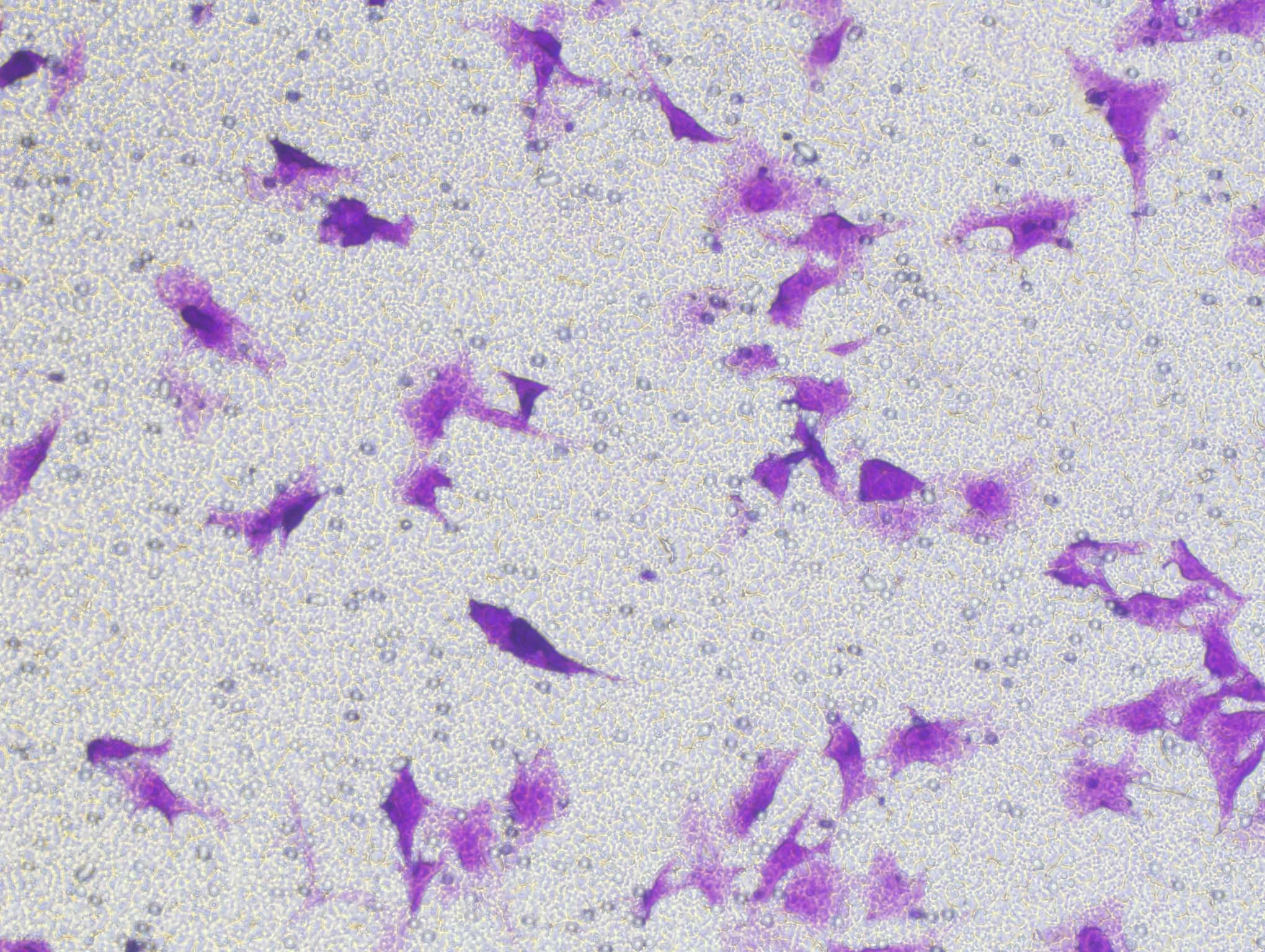

Supplement: Supplemental Information 2 [file peerj-11-15458-s002.zip › RawDataFig2/Fig2/Fig2C/invasion/THJ-16T sh-CTHRC1 01.jpg]

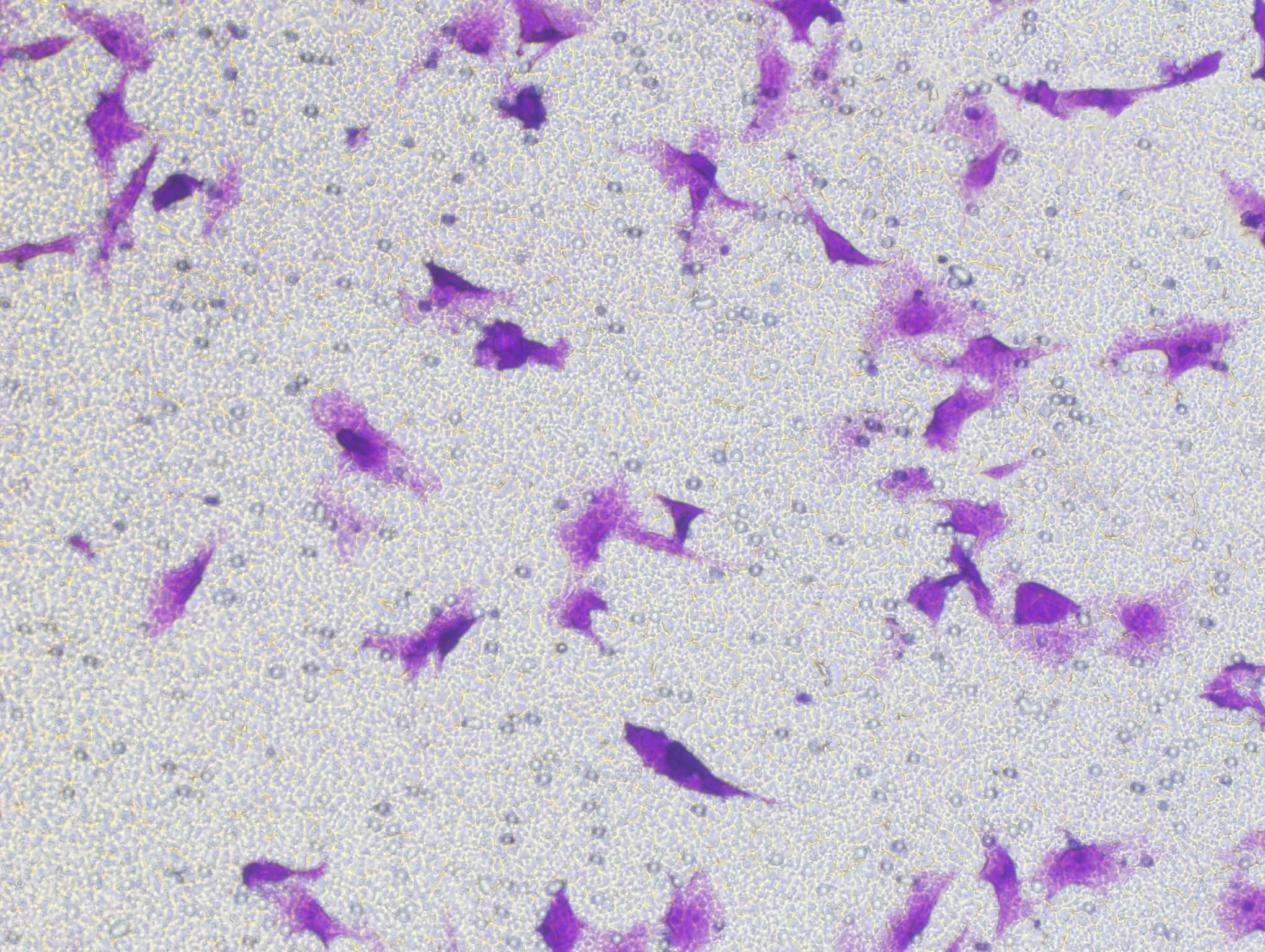

Supplement: Supplemental Information 2 [file peerj-11-15458-s002.zip › RawDataFig2/Fig2/Fig2C/invasion/THJ-16T sh-CTHRC1 02.jpg]

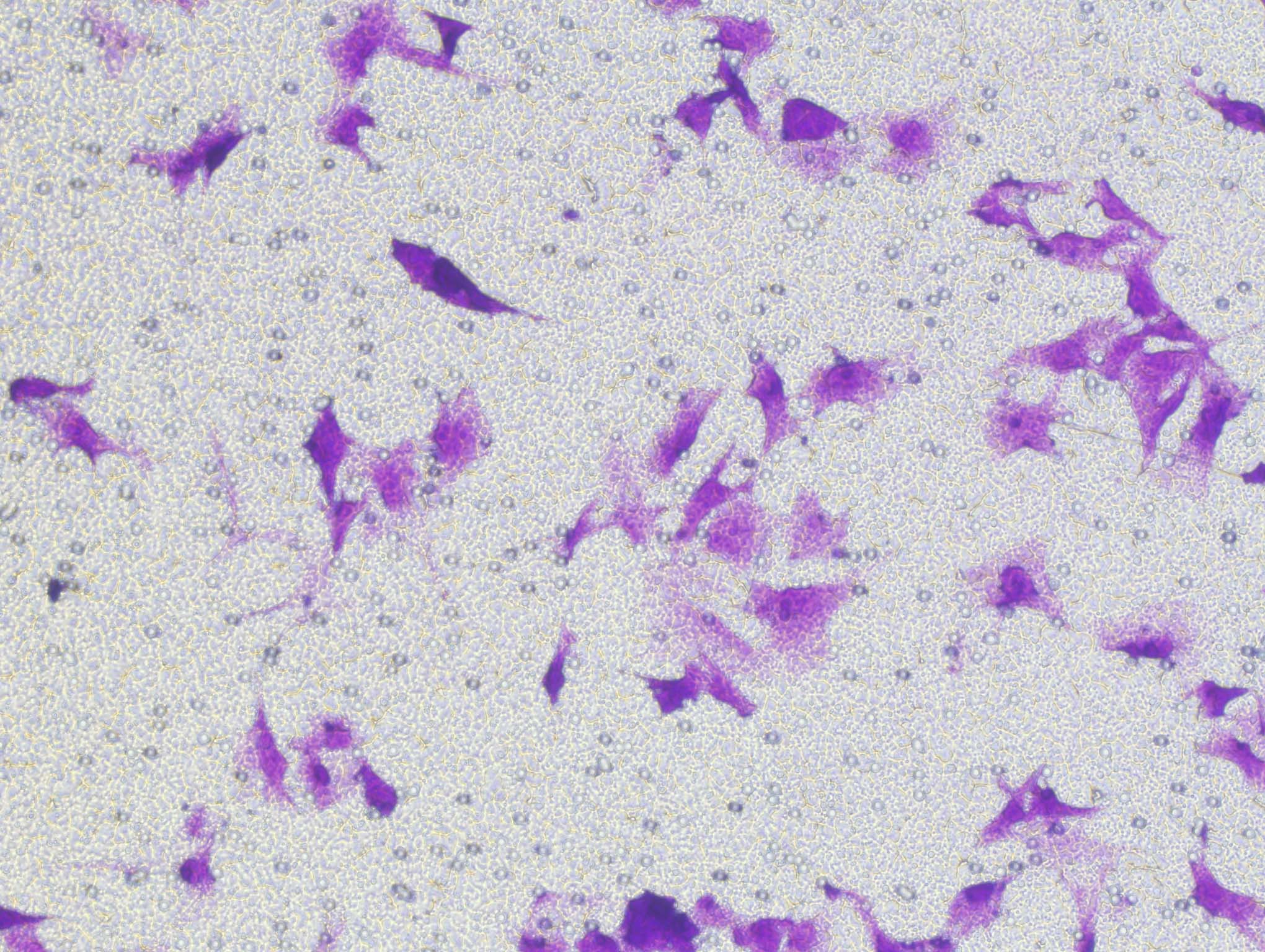

Supplement: Supplemental Information 2 [file peerj-11-15458-s002.zip › RawDataFig2/Fig2/Fig2C/invasion/THJ-16T sh-CTHRC1 03.jpg]

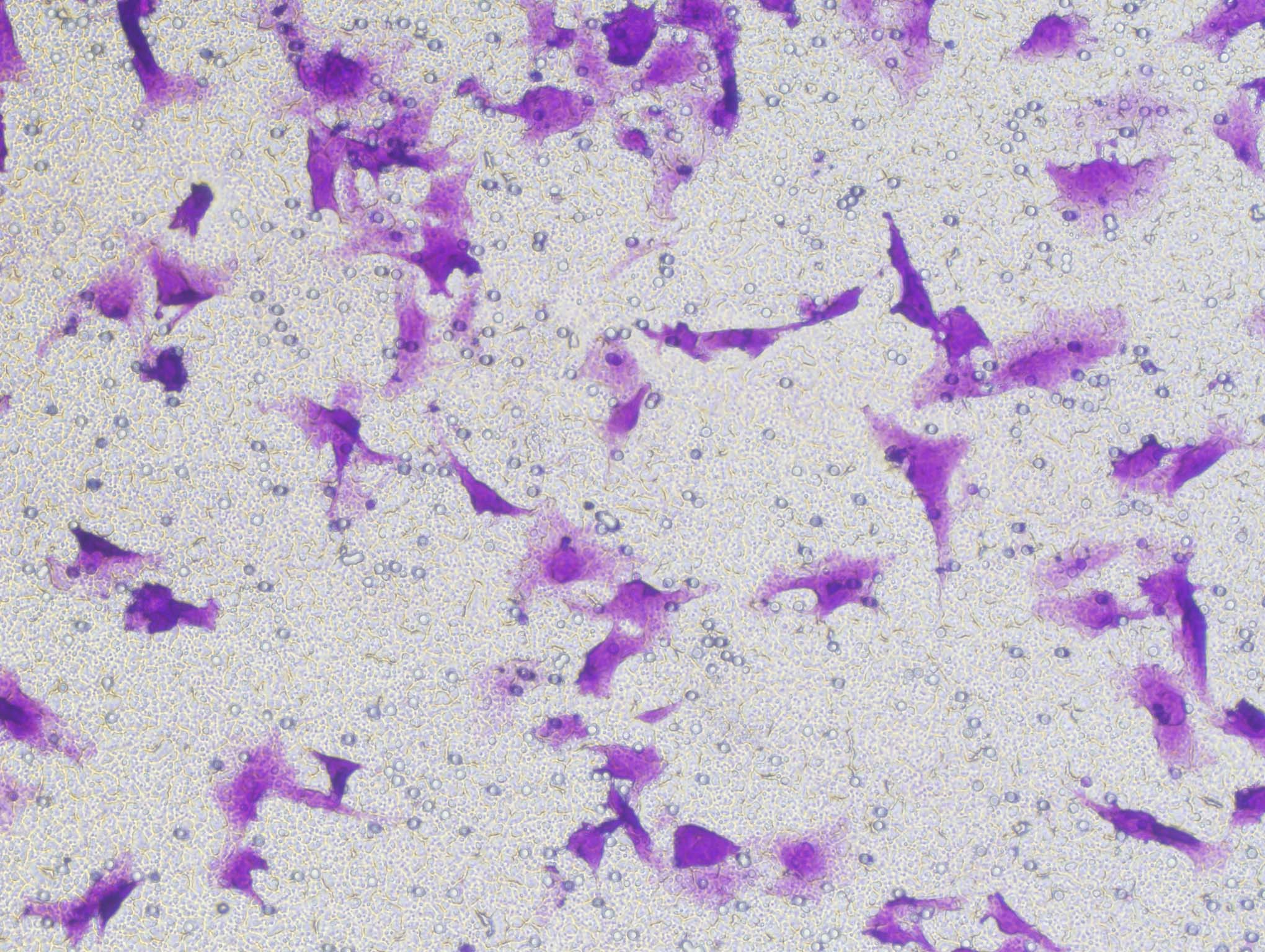

Supplement: Supplemental Information 2 [file peerj-11-15458-s002.zip › RawDataFig2/Fig2/Fig2C/invasion/THJ-16T sh-CTHRC1 04.jpg]

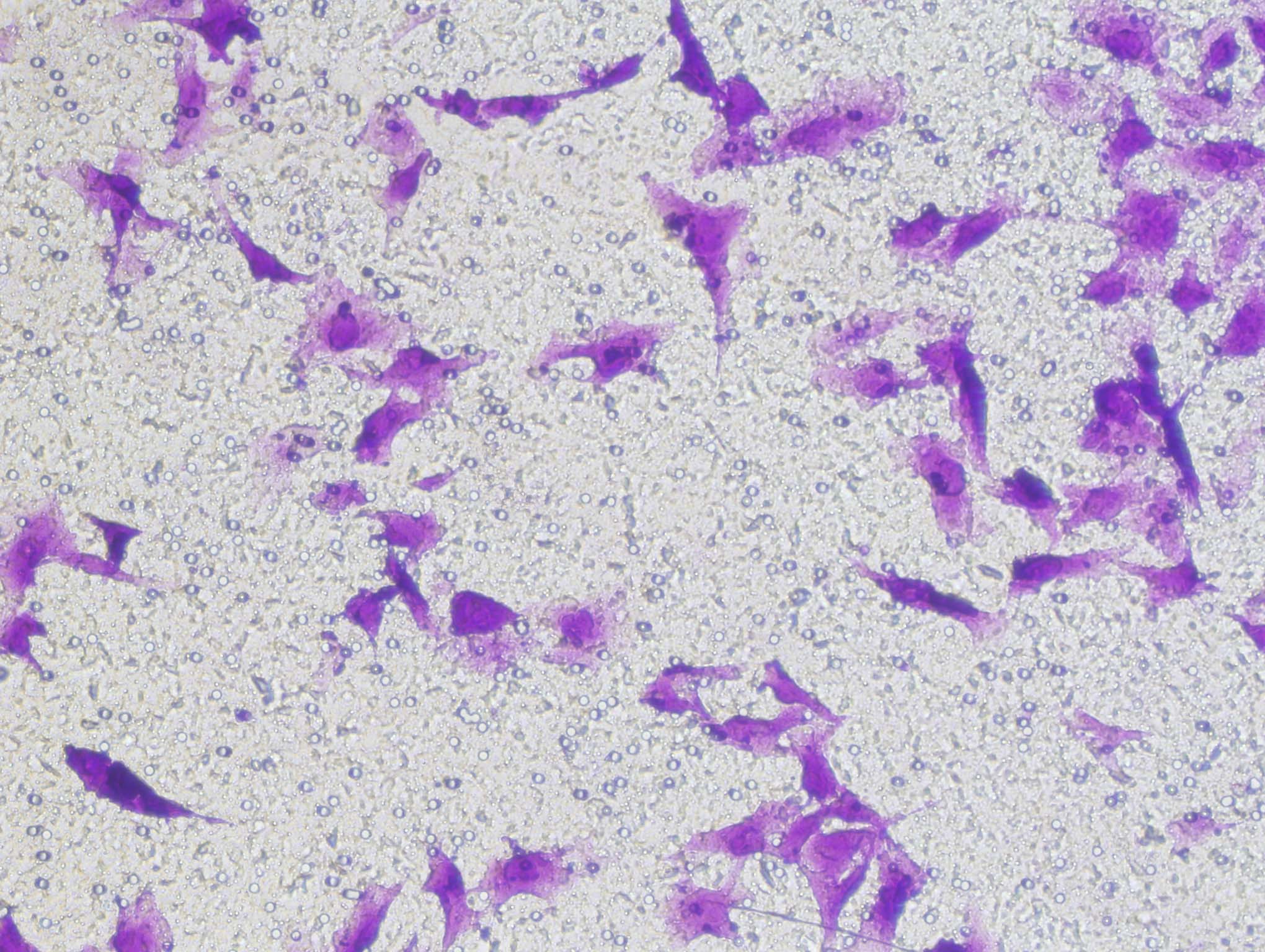

Supplement: Supplemental Information 2 [file peerj-11-15458-s002.zip › RawDataFig2/Fig2/Fig2C/invasion/THJ-16T sh-CTHRC1 05.jpg]

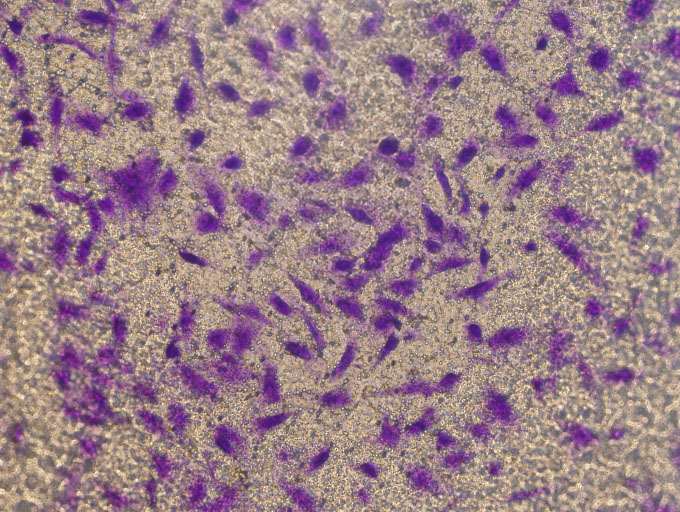

Supplement: Supplemental Information 2 [file peerj-11-15458-s002.zip › RawDataFig2/Fig2/Fig2D/Migration/T238 NC 01.jpg]

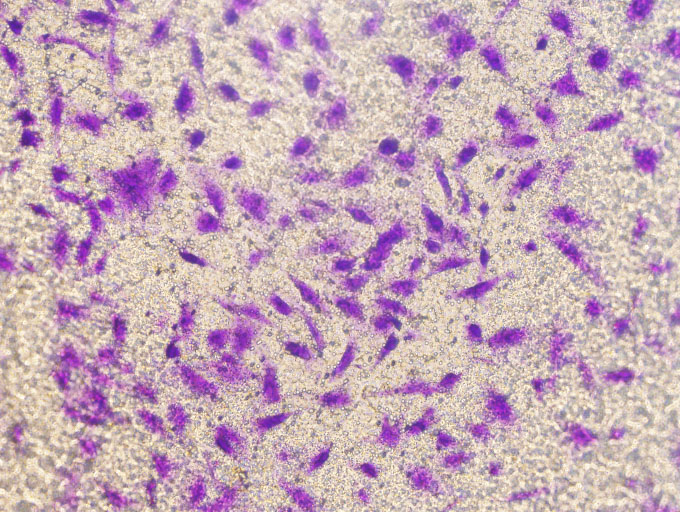

Supplement: Supplemental Information 2 [file peerj-11-15458-s002.zip › RawDataFig2/Fig2/Fig2D/Migration/T238 NC 02.jpg]

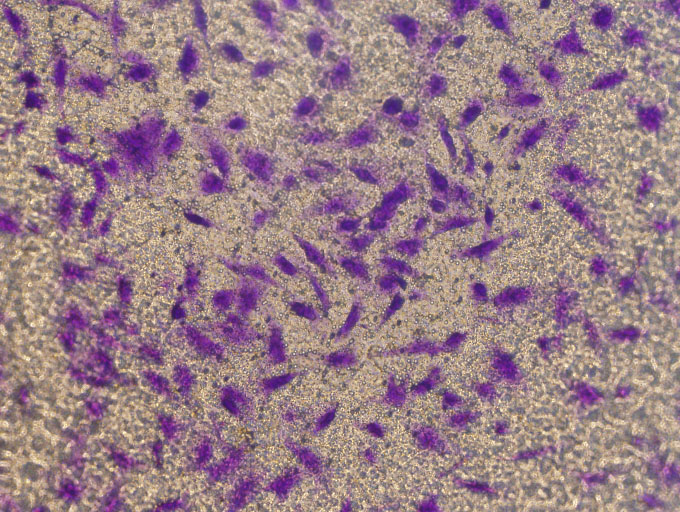

Supplement: Supplemental Information 2 [file peerj-11-15458-s002.zip › RawDataFig2/Fig2/Fig2D/Migration/T238 NC 03.jpg]

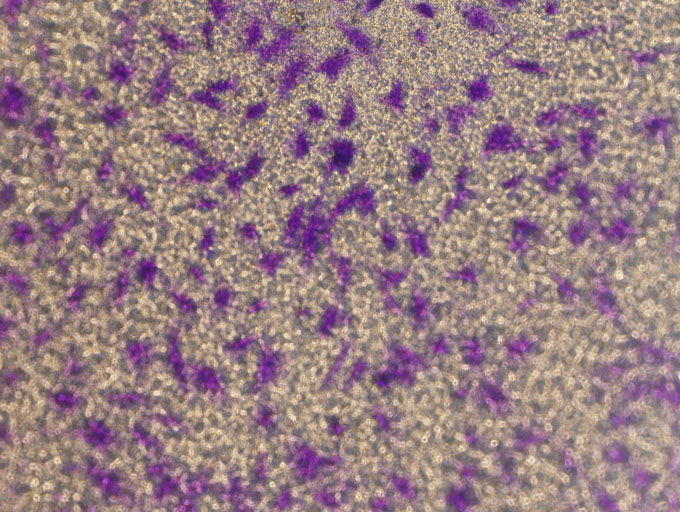

Supplement: Supplemental Information 2 [file peerj-11-15458-s002.zip › RawDataFig2/Fig2/Fig2D/Migration/T238 NC 04.jpg]

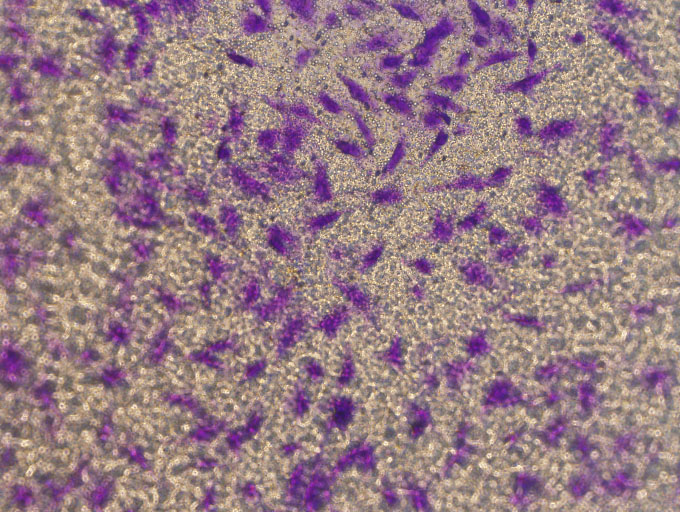

Supplement: Supplemental Information 2 [file peerj-11-15458-s002.zip › RawDataFig2/Fig2/Fig2D/Migration/T238 NC 05.jpg]

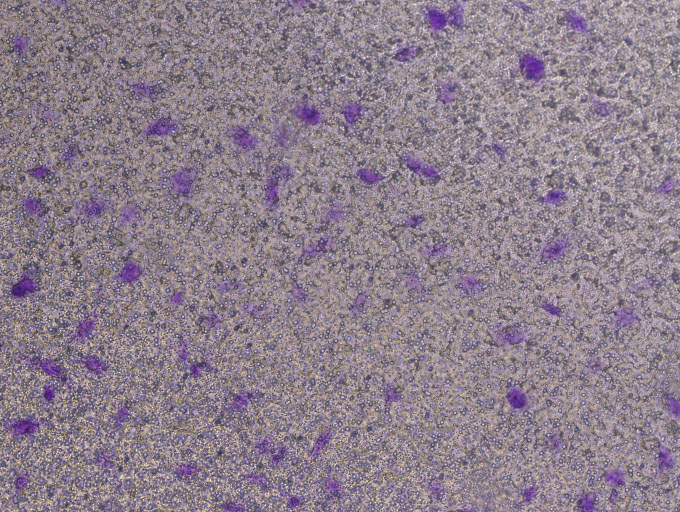

Supplement: Supplemental Information 2 [file peerj-11-15458-s002.zip › RawDataFig2/Fig2/Fig2D/Migration/T238 sh-CTHRC1 01.jpg]

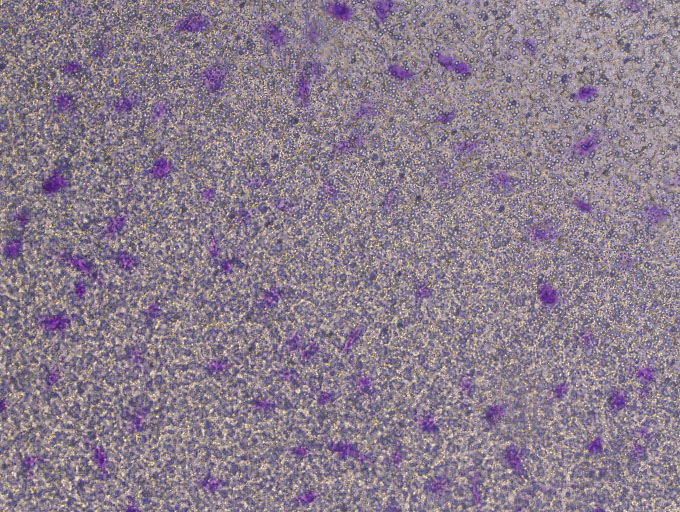

Supplement: Supplemental Information 2 [file peerj-11-15458-s002.zip › RawDataFig2/Fig2/Fig2D/Migration/T238 sh-CTHRC1 02.jpg]

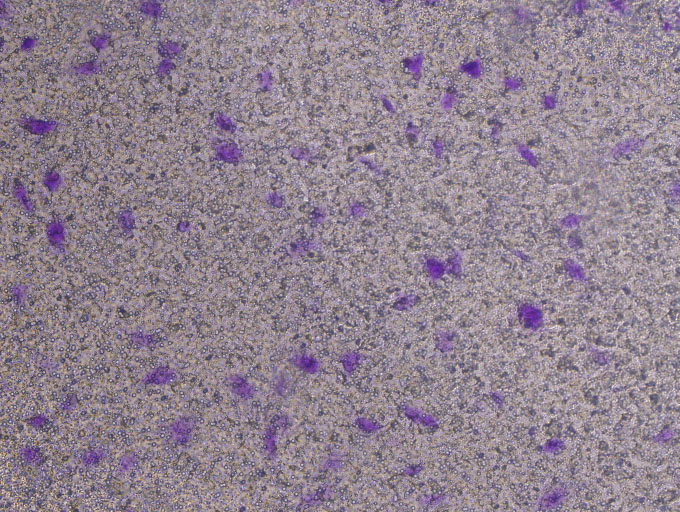

Supplement: Supplemental Information 2 [file peerj-11-15458-s002.zip › RawDataFig2/Fig2/Fig2D/Migration/T238 sh-CTHRC1 03.jpg]

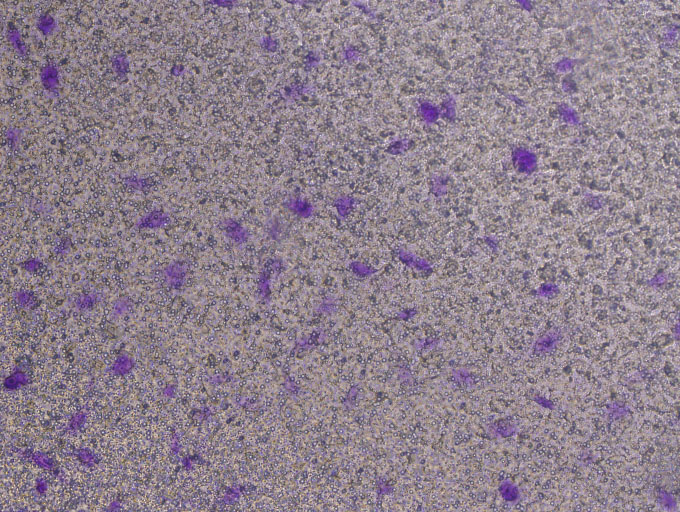

Supplement: Supplemental Information 2 [file peerj-11-15458-s002.zip › RawDataFig2/Fig2/Fig2D/Migration/T238 sh-CTHRC1 04.jpg]

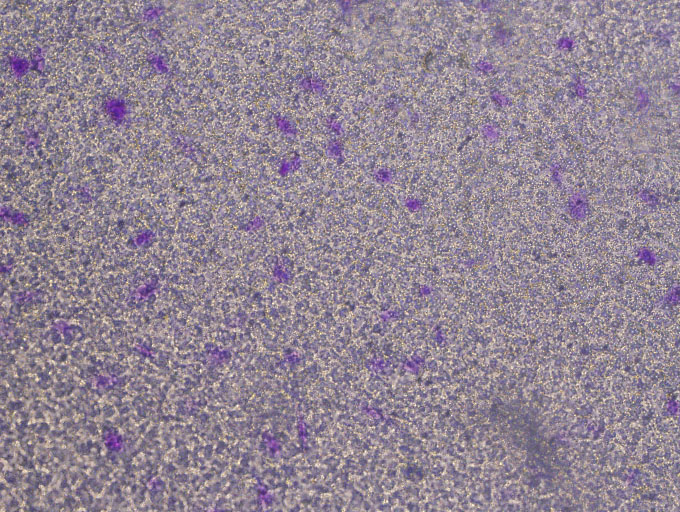

Supplement: Supplemental Information 2 [file peerj-11-15458-s002.zip › RawDataFig2/Fig2/Fig2D/Migration/T238 sh-CTHRC1 05.jpg]

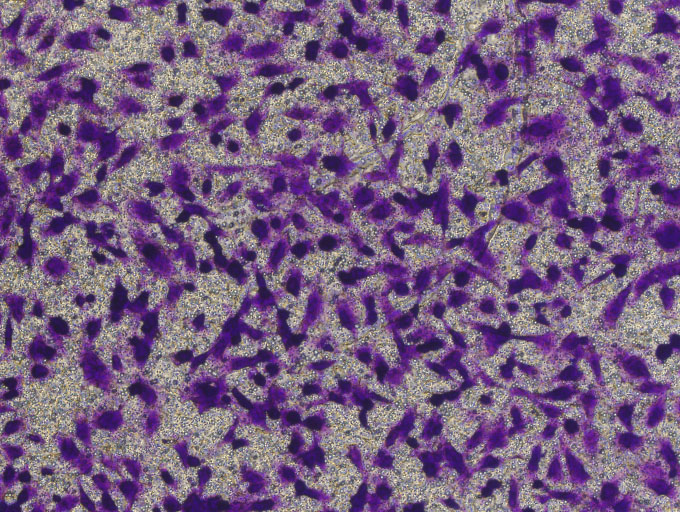

Supplement: Supplemental Information 2 [file peerj-11-15458-s002.zip › RawDataFig2/Fig2/Fig2D/Migration/THJ-16T NC 01.jpg]

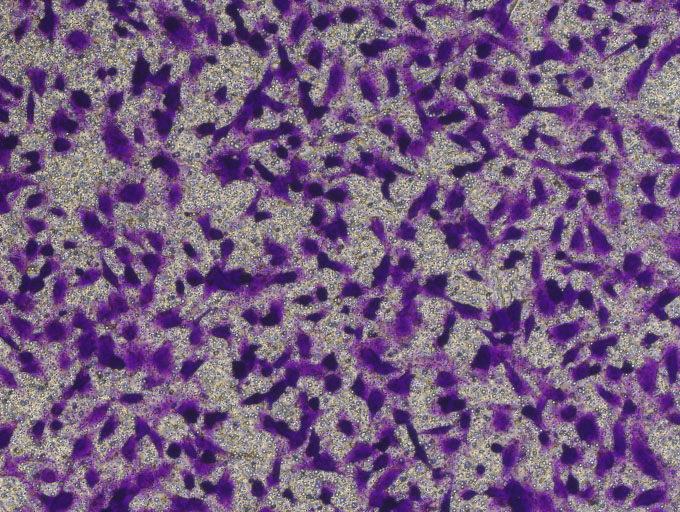

Supplement: Supplemental Information 2 [file peerj-11-15458-s002.zip › RawDataFig2/Fig2/Fig2D/Migration/THJ-16T NC 02.jpg]

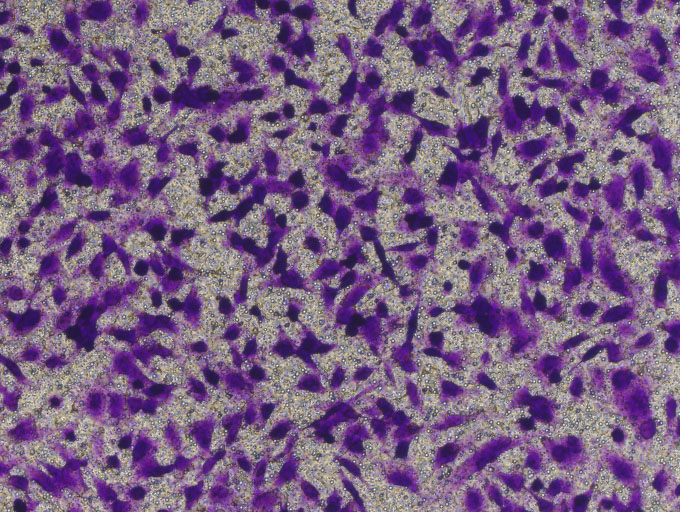

Supplement: Supplemental Information 2 [file peerj-11-15458-s002.zip › RawDataFig2/Fig2/Fig2D/Migration/THJ-16T NC 03.jpg]

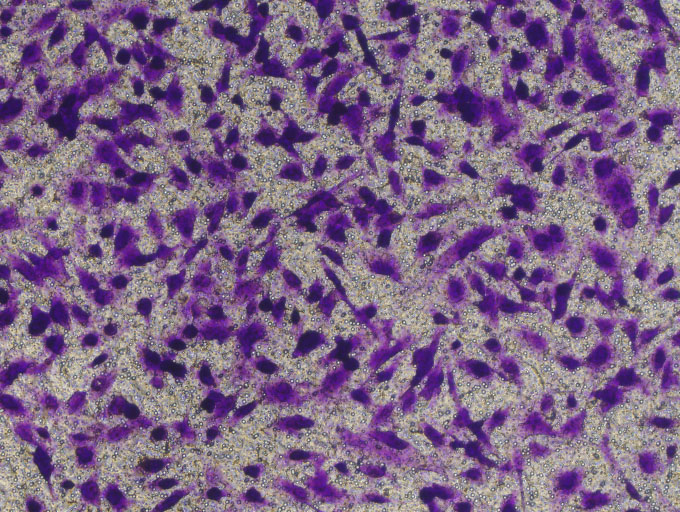

Supplement: Supplemental Information 2 [file peerj-11-15458-s002.zip › RawDataFig2/Fig2/Fig2D/Migration/THJ-16T NC 04.jpg]

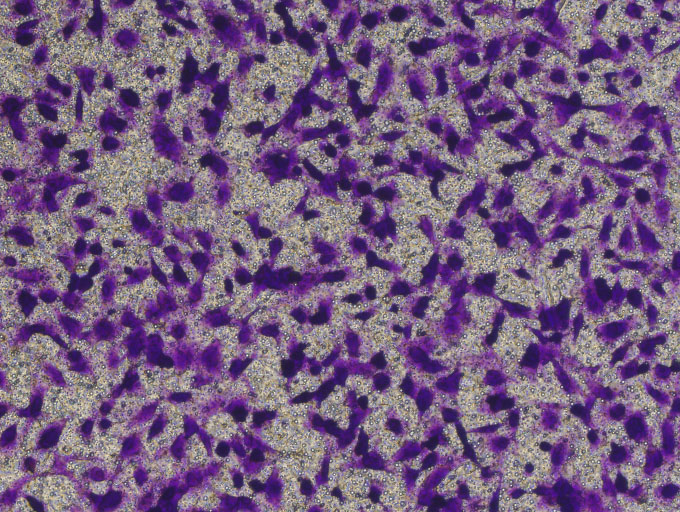

Supplement: Supplemental Information 2 [file peerj-11-15458-s002.zip › RawDataFig2/Fig2/Fig2D/Migration/THJ-16T NC 05.jpg]

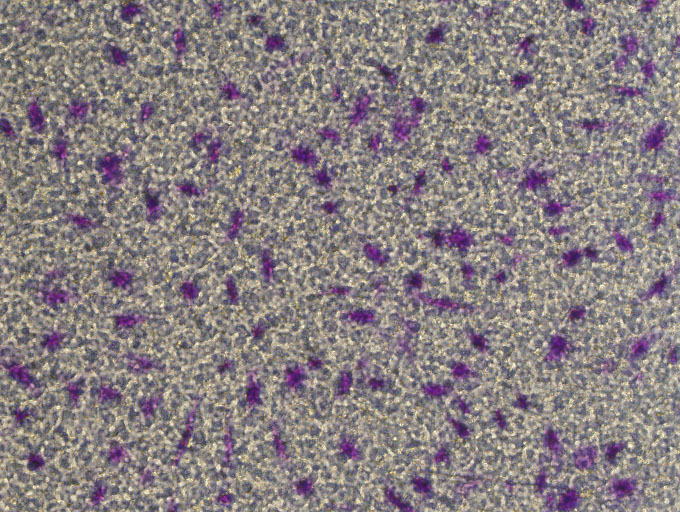

Supplement: Supplemental Information 2 [file peerj-11-15458-s002.zip › RawDataFig2/Fig2/Fig2D/Migration/THJ-16T sh-CTHRC1 01.jpg]

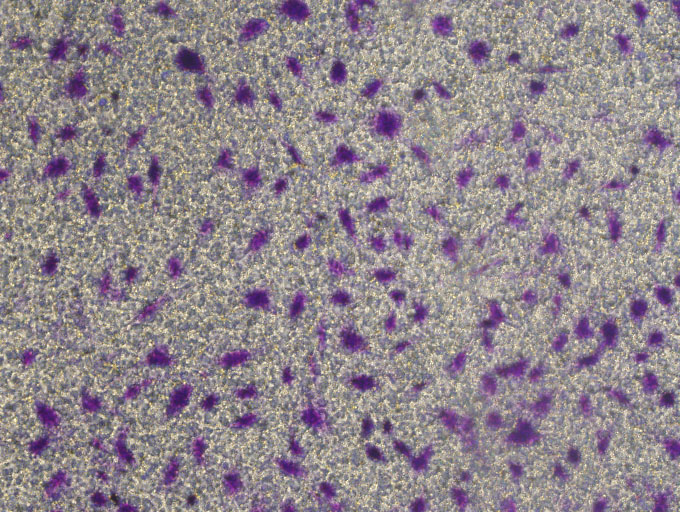

Supplement: Supplemental Information 2 [file peerj-11-15458-s002.zip › RawDataFig2/Fig2/Fig2D/Migration/THJ-16T sh-CTHRC1 02.jpg]

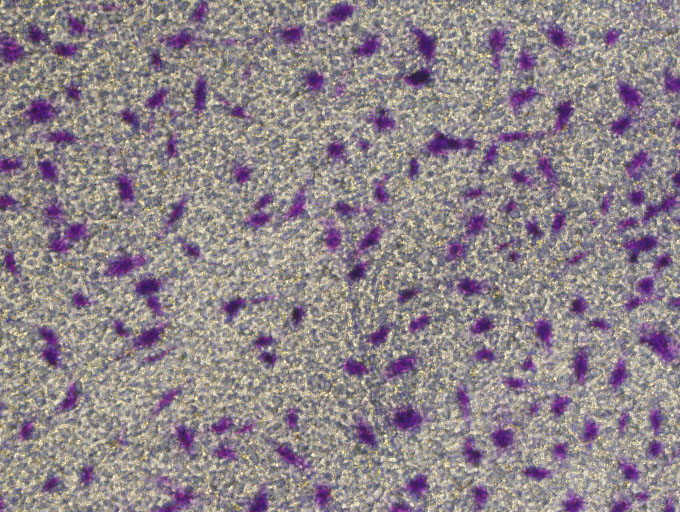

Supplement: Supplemental Information 2 [file peerj-11-15458-s002.zip › RawDataFig2/Fig2/Fig2D/Migration/THJ-16T sh-CTHRC1 03.jpg]

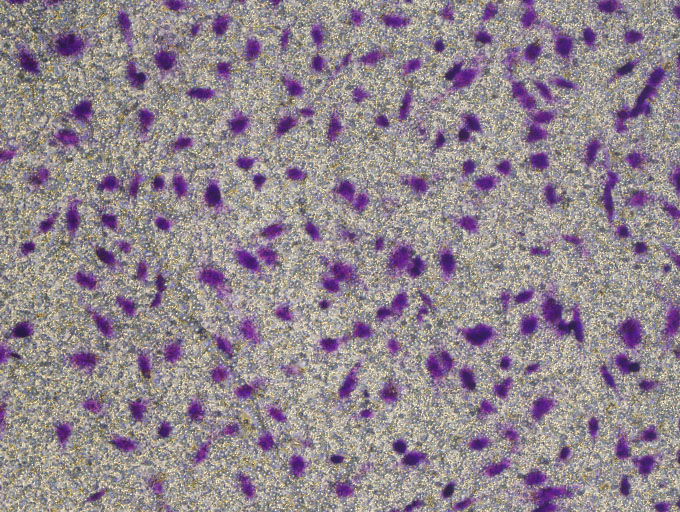

Supplement: Supplemental Information 2 [file peerj-11-15458-s002.zip › RawDataFig2/Fig2/Fig2D/Migration/THJ-16T sh-CTHRC1 04.jpg]

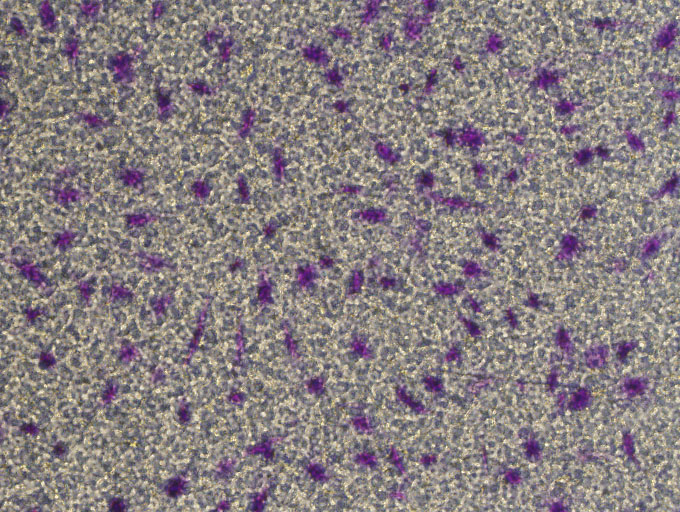

Supplement: Supplemental Information 2 [file peerj-11-15458-s002.zip › RawDataFig2/Fig2/Fig2D/Migration/THJ-16T sh-CTHRC1 05.jpg]

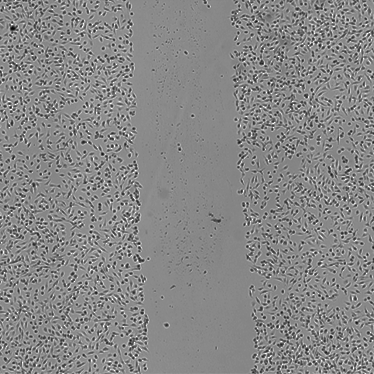

Supplement: Supplemental Information 3 [file peerj-11-15458-s003.zip › Fig.3/T238 wound healing assays/T238 NC 0h.png]

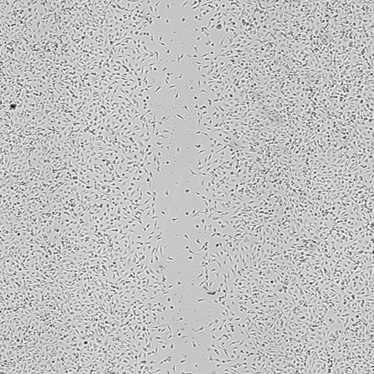

Supplement: Supplemental Information 3 [file peerj-11-15458-s003.zip › Fig.3/T238 wound healing assays/T238 NC 24h.png]

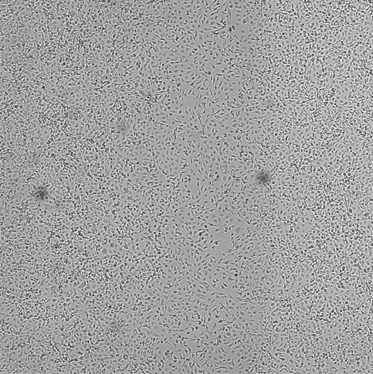

Supplement: Supplemental Information 3 [file peerj-11-15458-s003.zip › Fig.3/T238 wound healing assays/T238 NC 48h.png]

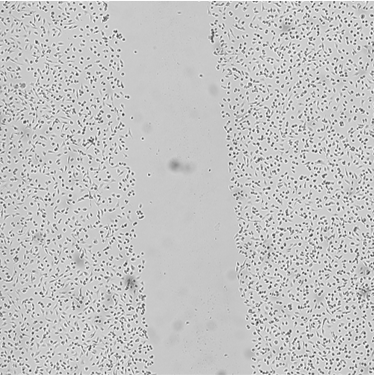

Supplement: Supplemental Information 3 [file peerj-11-15458-s003.zip › Fig.3/T238 wound healing assays/T238 shCTHRC1 0h.png]

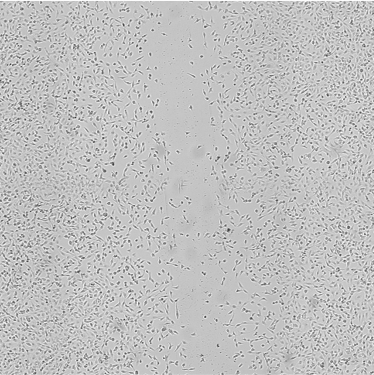

Supplement: Supplemental Information 3 [file peerj-11-15458-s003.zip › Fig.3/T238 wound healing assays/T238 shCTHRC1 24h.png]

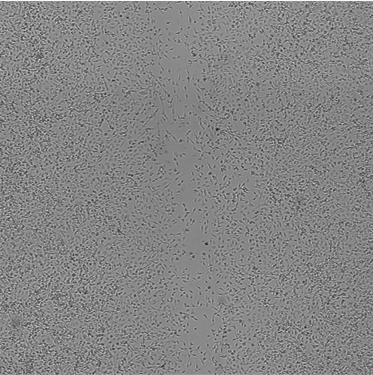

Supplement: Supplemental Information 3 [file peerj-11-15458-s003.zip › Fig.3/T238 wound healing assays/T238 shCTHRC1 48h.png]

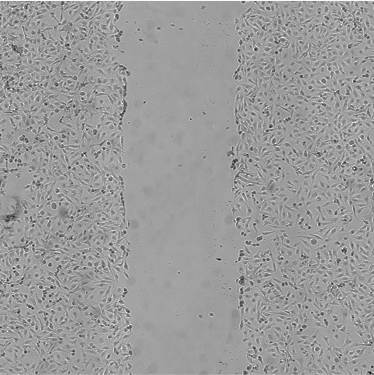

Supplement: Supplemental Information 3 [file peerj-11-15458-s003.zip › Fig.3/THJ-16T wound healing assays/THJ-16 sh-CTHRC1 0h.png]

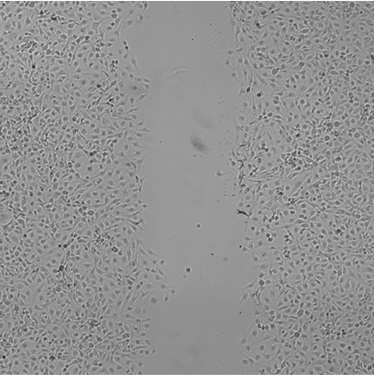

Supplement: Supplemental Information 3 [file peerj-11-15458-s003.zip › Fig.3/THJ-16T wound healing assays/THJ-16 sh-CTHRC1 24h.png]

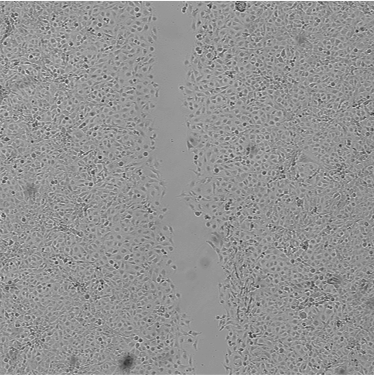

Supplement: Supplemental Information 3 [file peerj-11-15458-s003.zip › Fig.3/THJ-16T wound healing assays/THJ-16 sh-CTHRC1 48h.png]

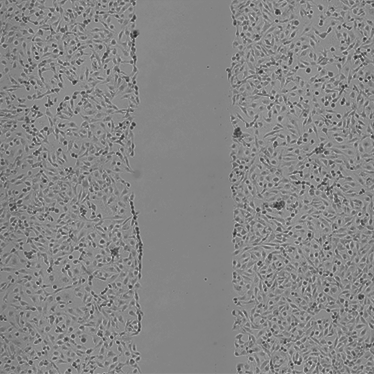

Supplement: Supplemental Information 3 [file peerj-11-15458-s003.zip › Fig.3/THJ-16T wound healing assays/THJ-16T NC 0h.png]

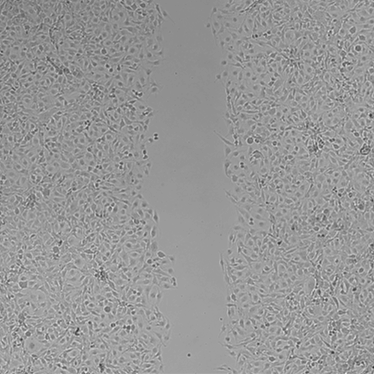

Supplement: Supplemental Information 3 [file peerj-11-15458-s003.zip › Fig.3/THJ-16T wound healing assays/THJ-16T NC 24h.png]

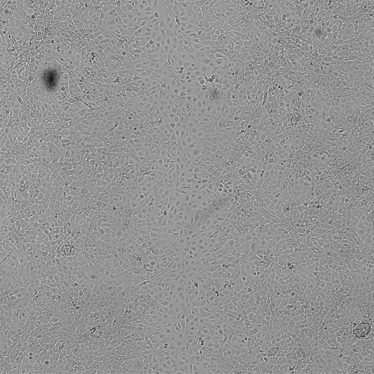

Supplement: Supplemental Information 3 [file peerj-11-15458-s003.zip › Fig.3/THJ-16T wound healing assays/THJ-16T NC 48h.png]

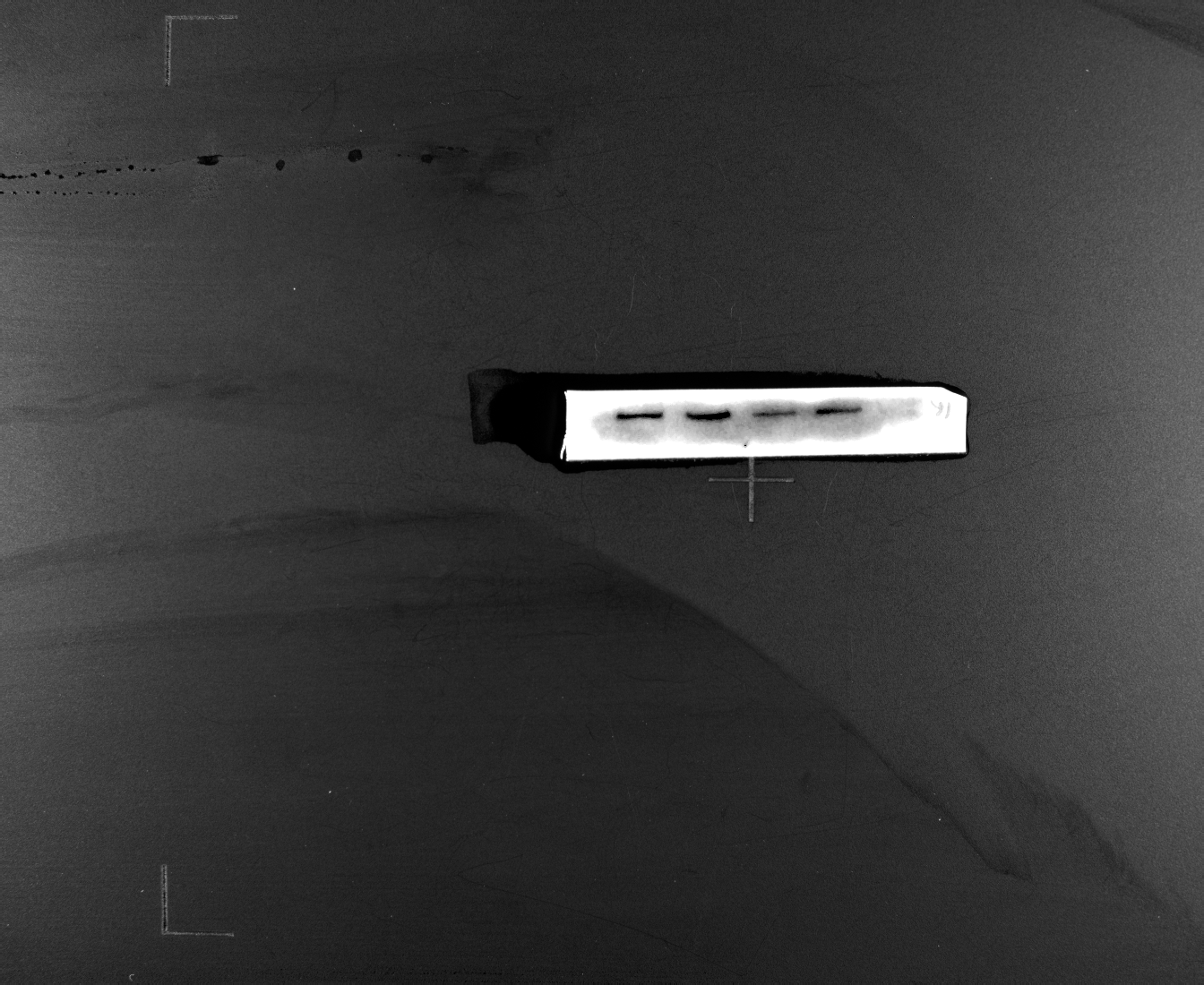

Supplement: Supplemental Information 5 [file peerj-11-15458-s005.zip › Fig.5/E-cadherin 01.png]

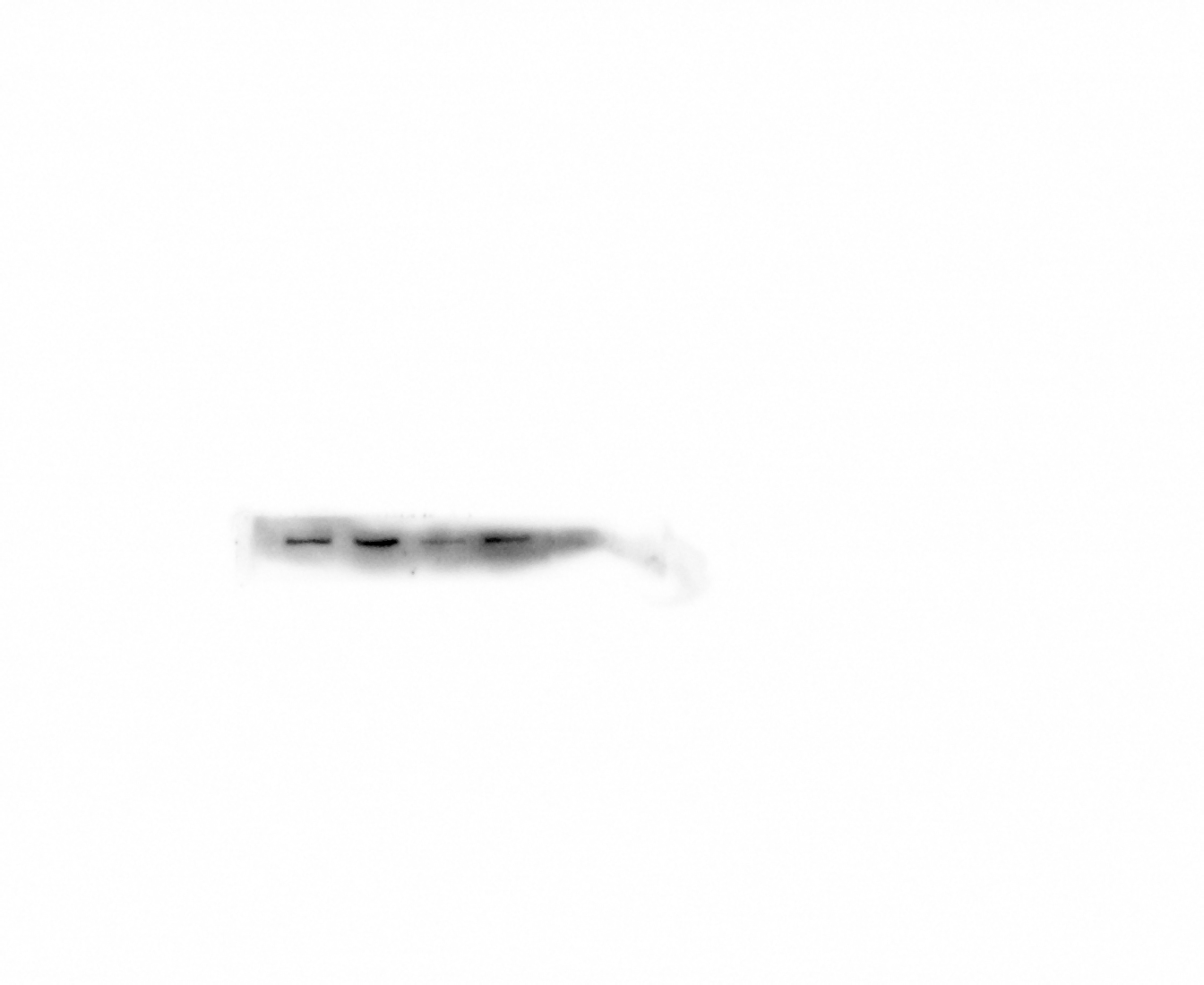

Supplement: Supplemental Information 5 [file peerj-11-15458-s005.zip › Fig.5/E-cadherin 02.png]

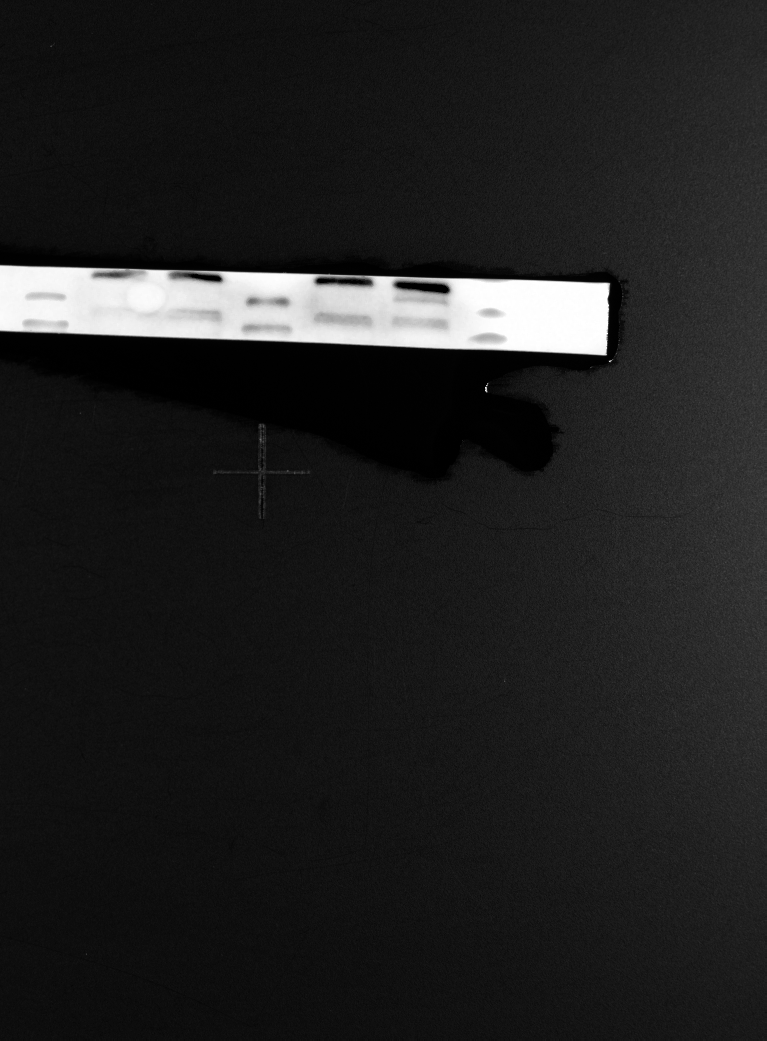

Supplement: Supplemental Information 5 [file peerj-11-15458-s005.zip › Fig.5/E-cadherin 03.png]

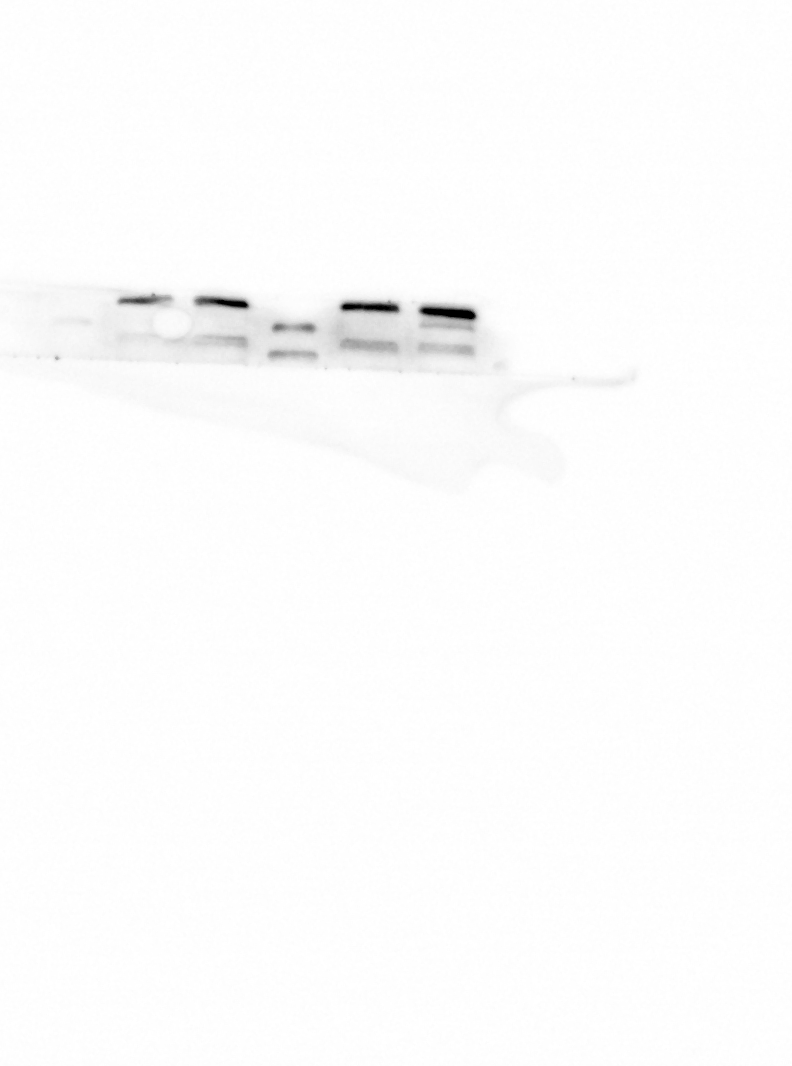

Supplement: Supplemental Information 5 [file peerj-11-15458-s005.zip › Fig.5/E-cadherin 04.png]

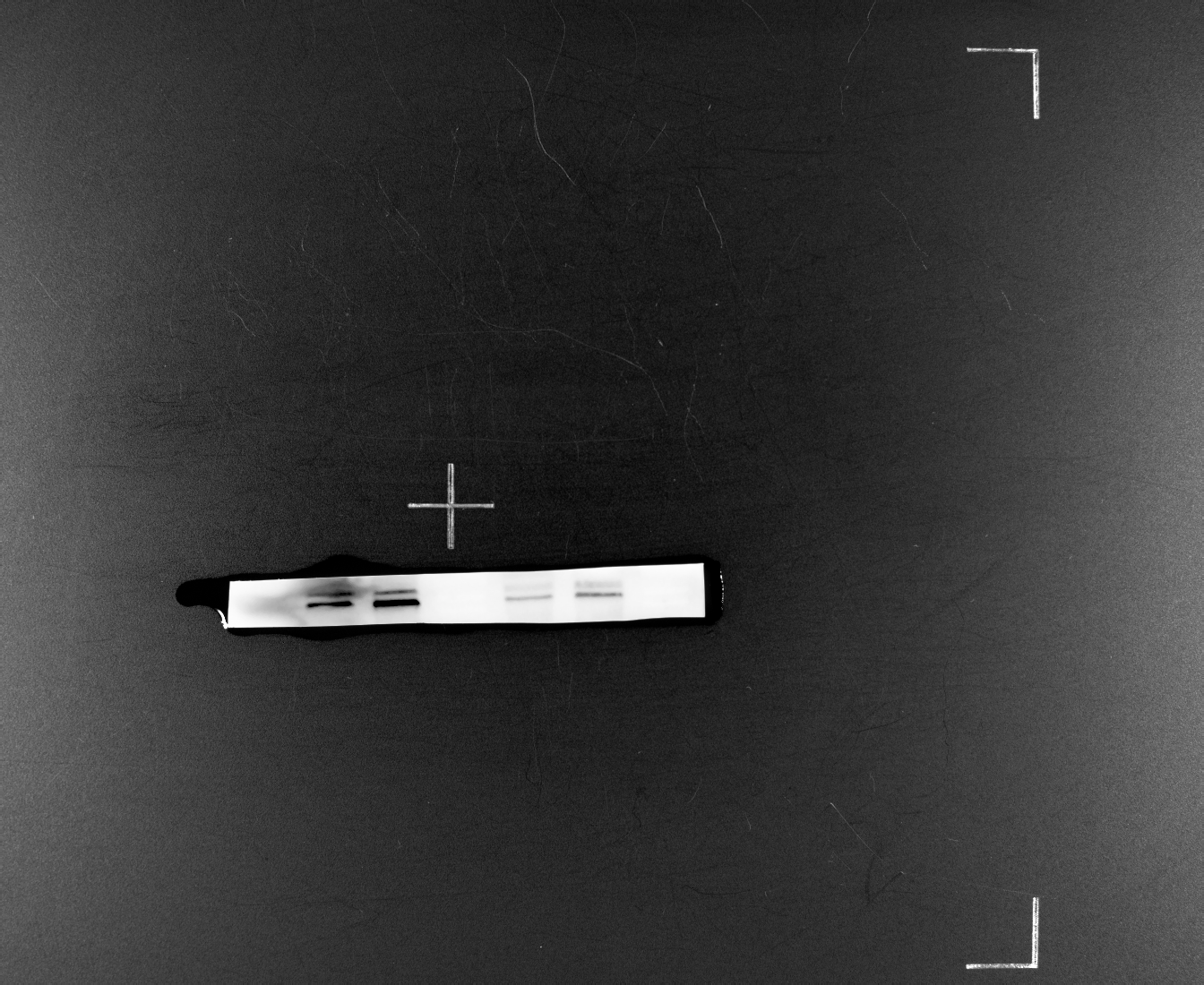

Supplement: Supplemental Information 5 [file peerj-11-15458-s005.zip › Fig.5/E-cadherin 05.png]

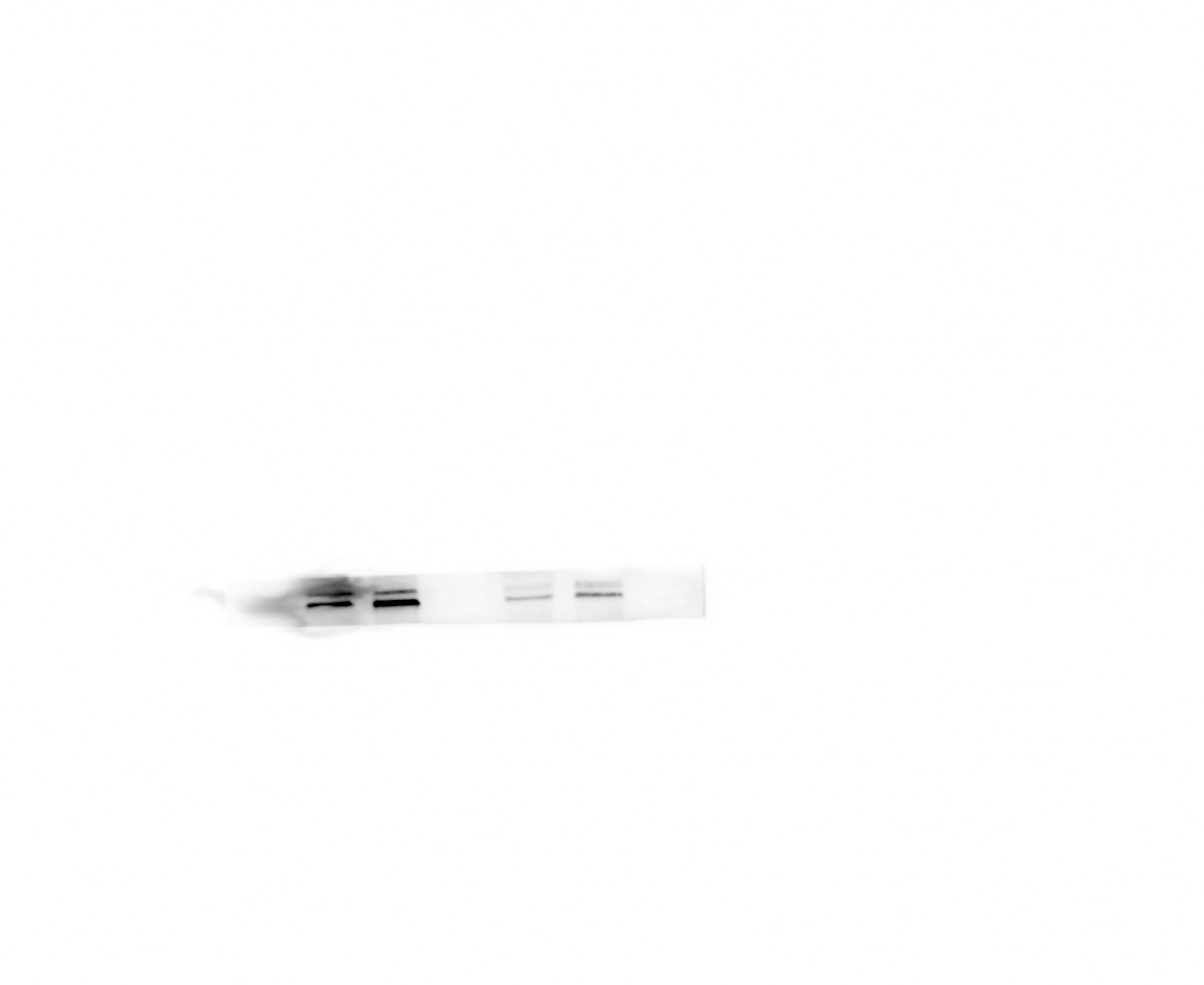

Supplement: Supplemental Information 5 [file peerj-11-15458-s005.zip › Fig.5/E-cadherin 06.png]

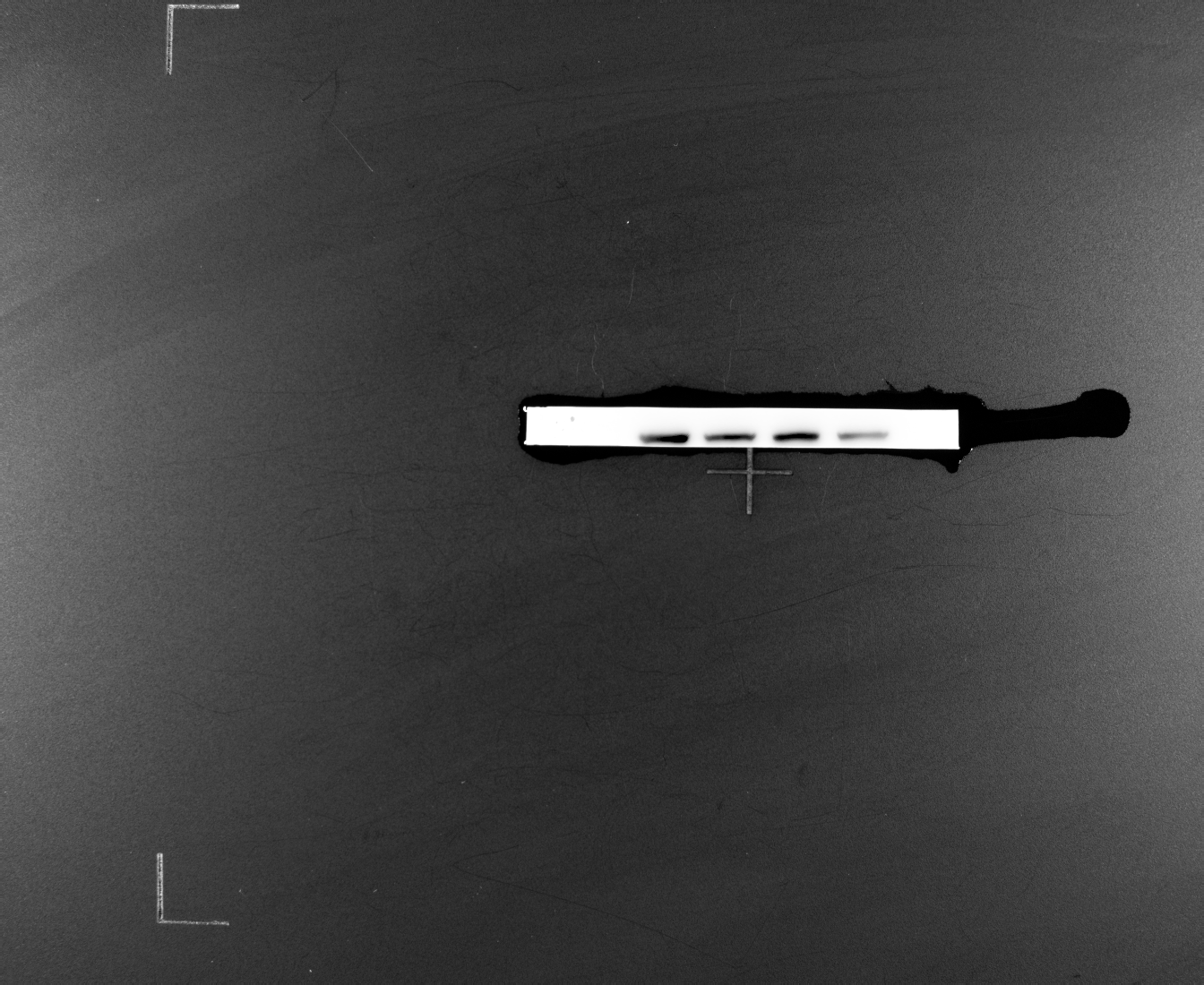

Supplement: Supplemental Information 5 [file peerj-11-15458-s005.zip › Fig.5/Vimentin 01.png]

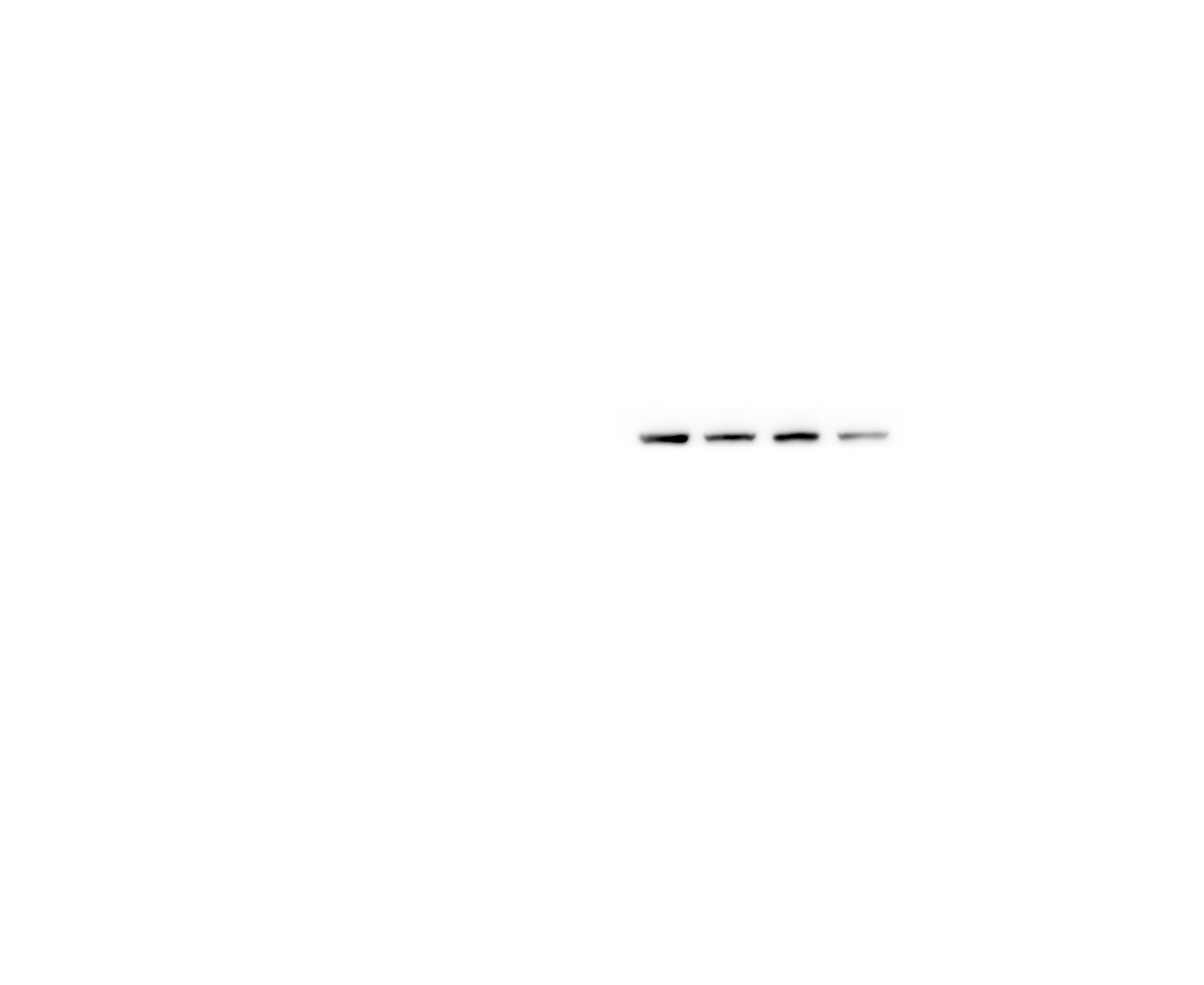

Supplement: Supplemental Information 5 [file peerj-11-15458-s005.zip › Fig.5/Vimentin 02.png]

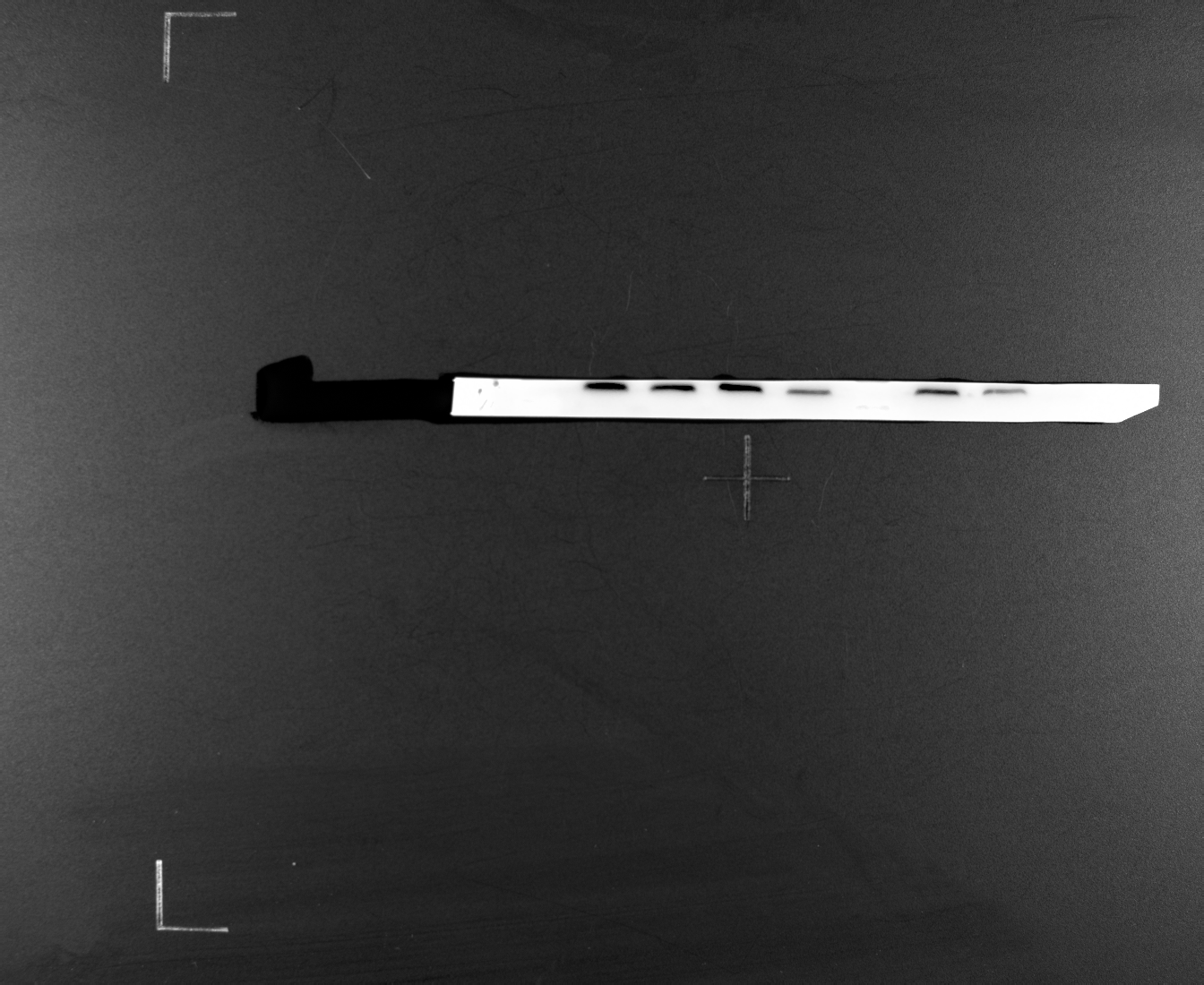

Supplement: Supplemental Information 5 [file peerj-11-15458-s005.zip › Fig.5/Vimentin 03.png]

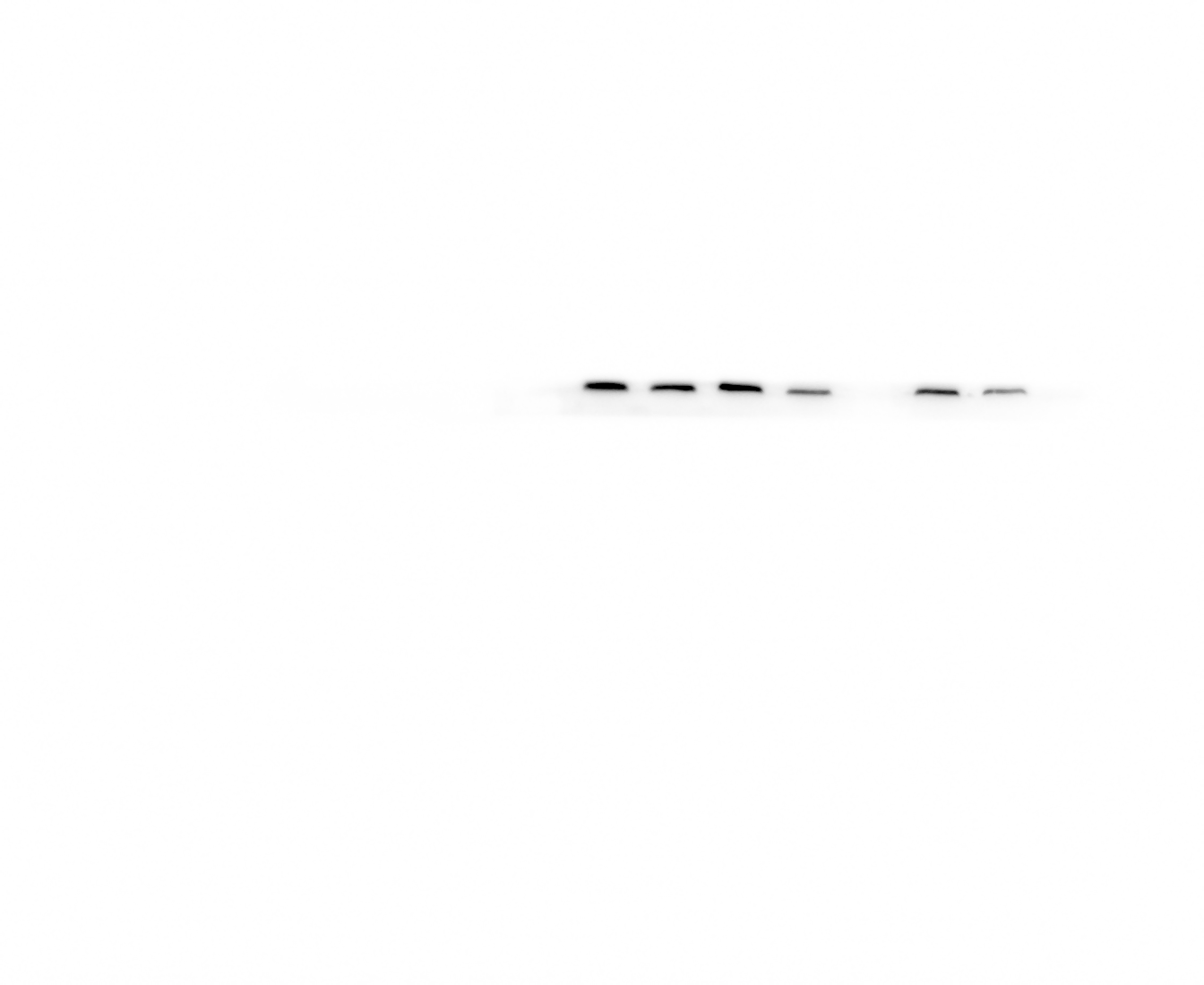

Supplement: Supplemental Information 5 [file peerj-11-15458-s005.zip › Fig.5/Vimentin 04.png]

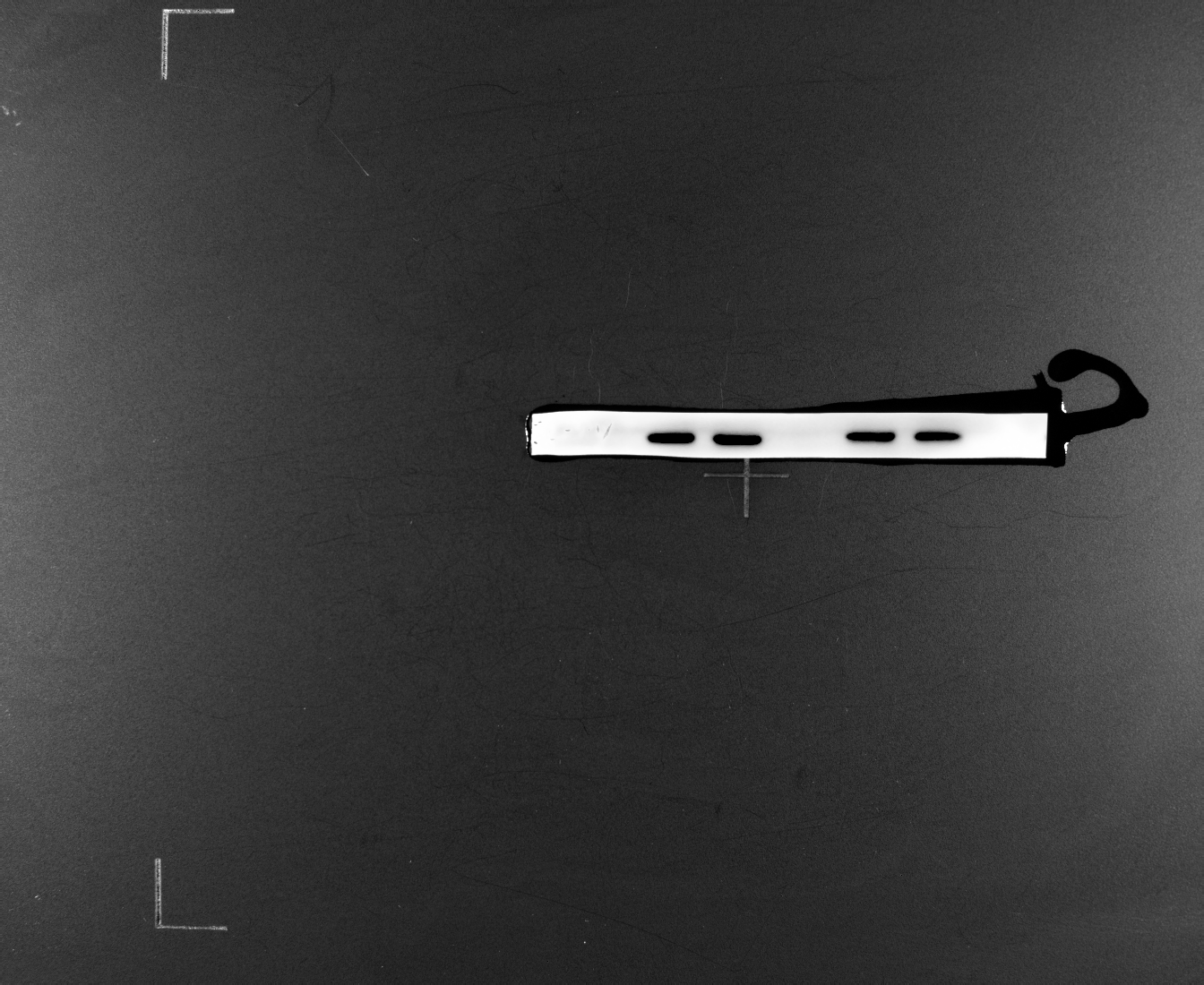

Supplement: Supplemental Information 5 [file peerj-11-15458-s005.zip › Fig.5/beta-actin 03.png]

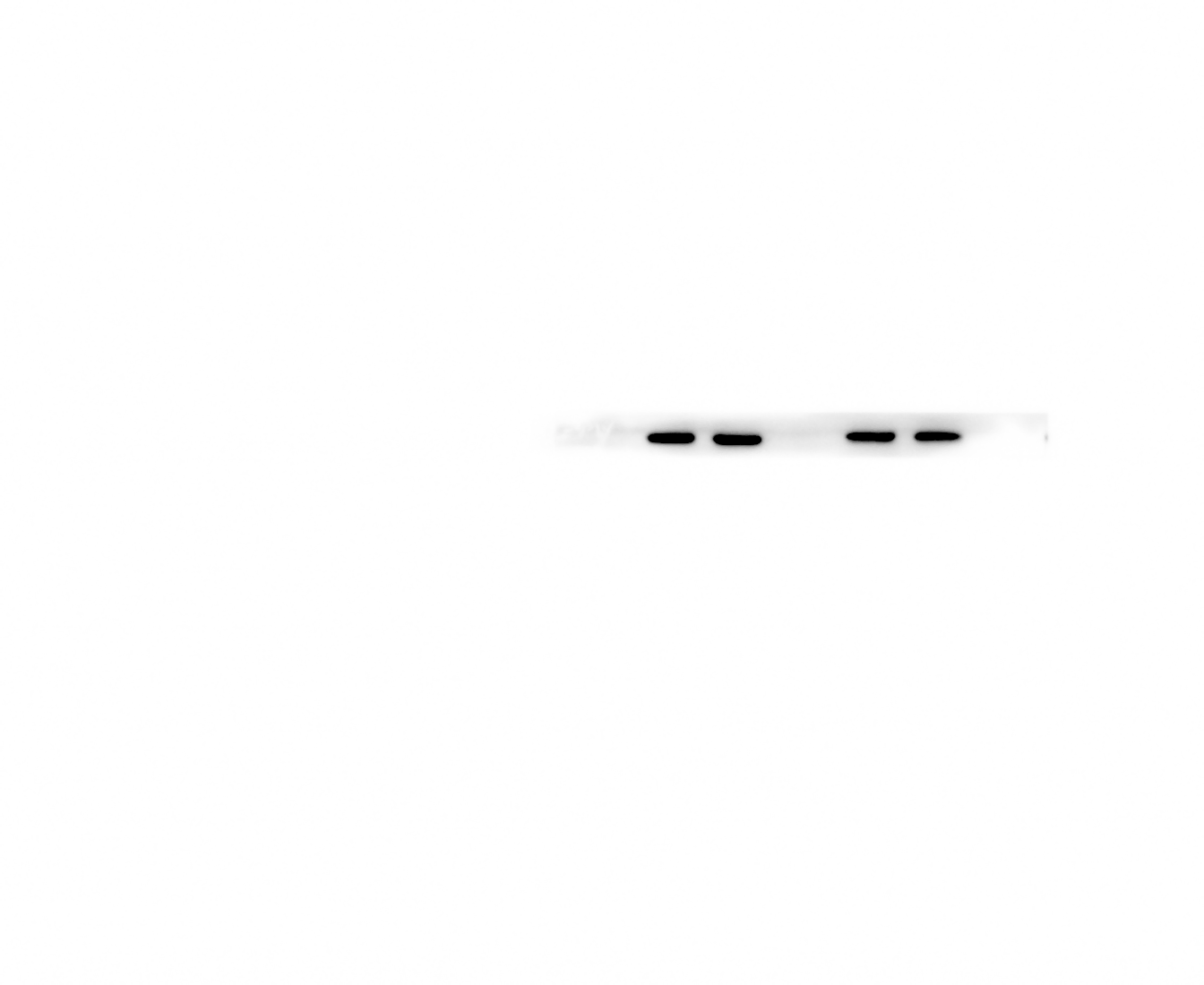

Supplement: Supplemental Information 5 [file peerj-11-15458-s005.zip › Fig.5/beta-actin 04.png]

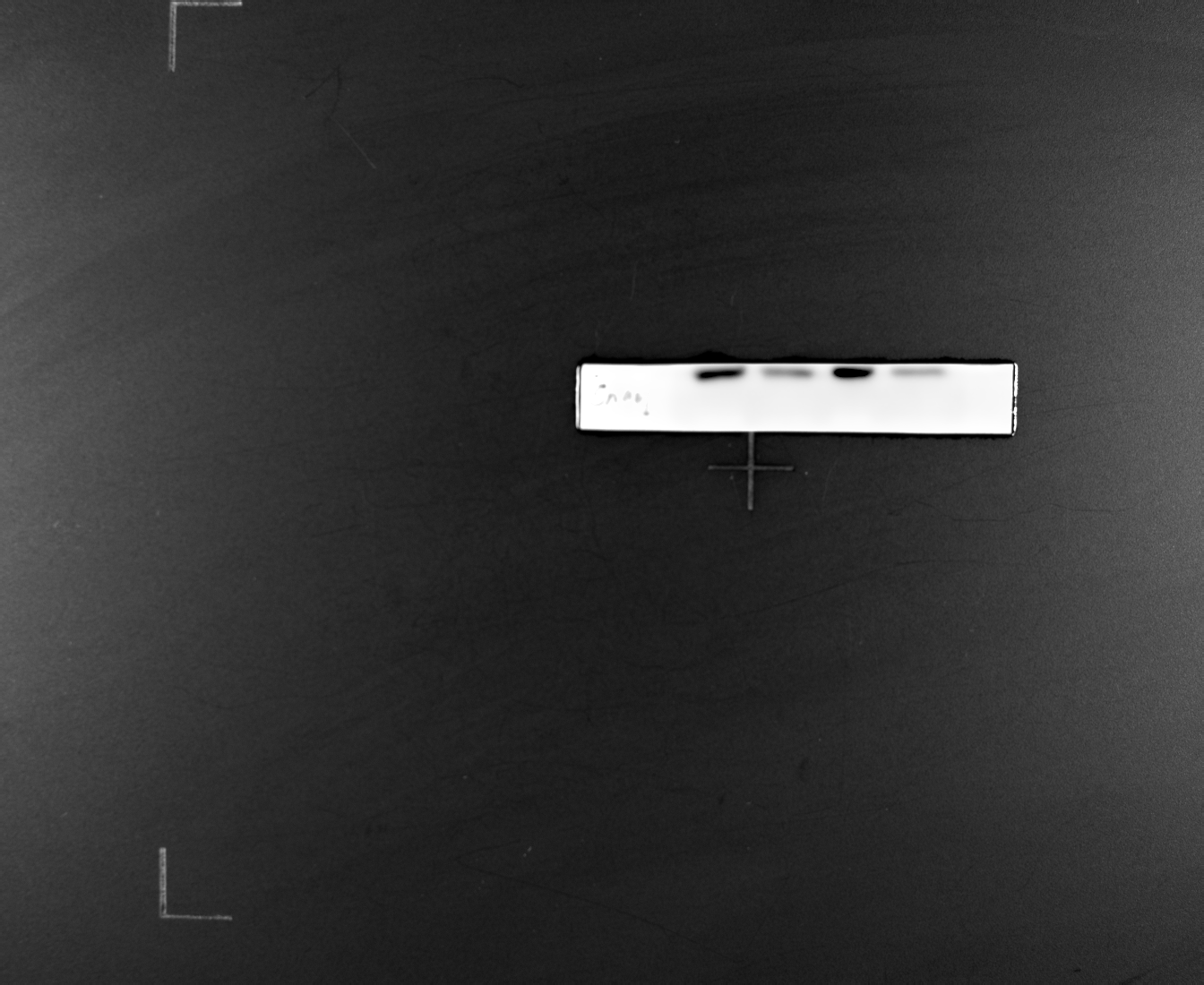

Supplement: Supplemental Information 5 [file peerj-11-15458-s005.zip › Fig.5/snail 01.png]

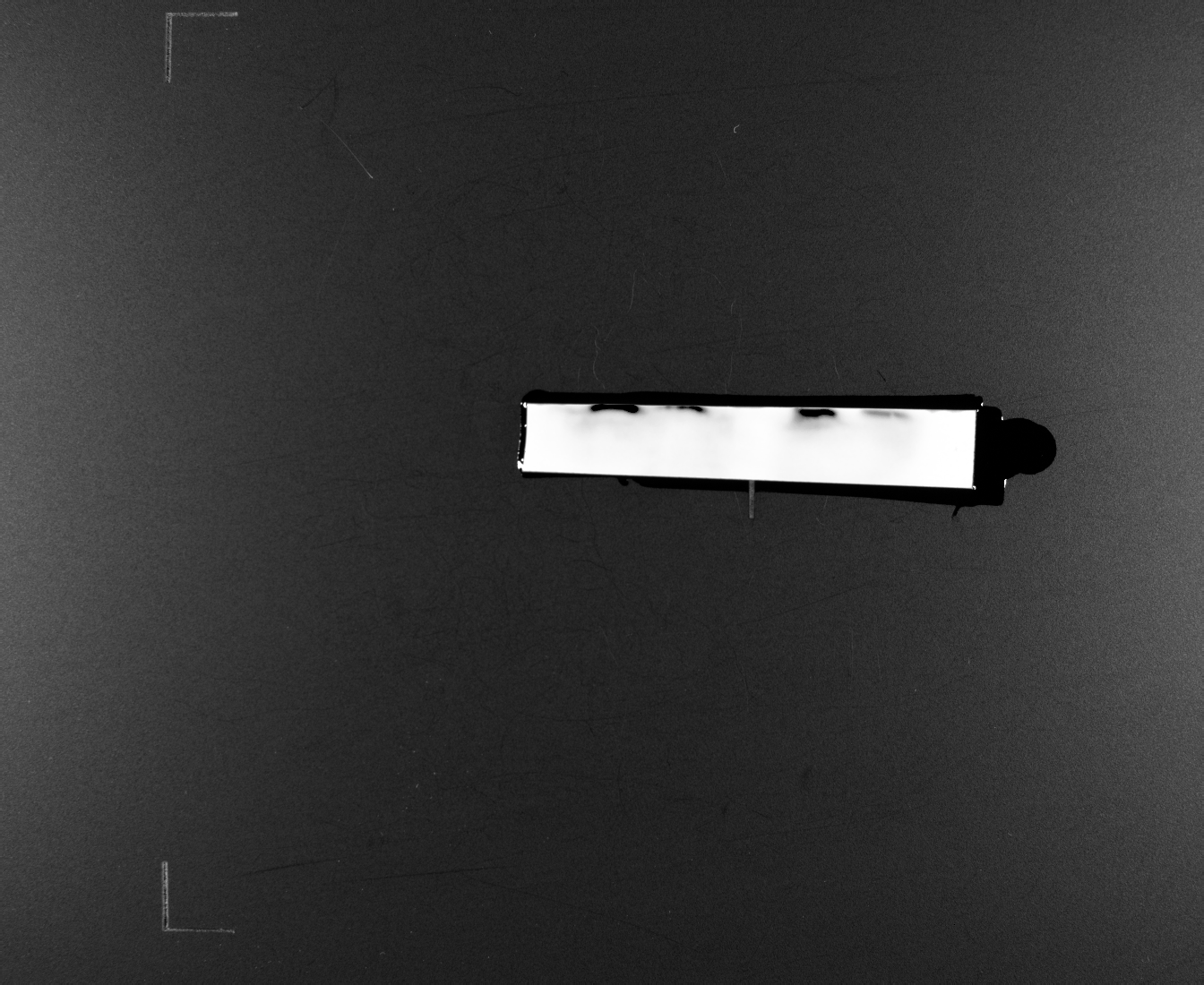

Supplement: Supplemental Information 5 [file peerj-11-15458-s005.zip › Fig.5/snail 03.png]

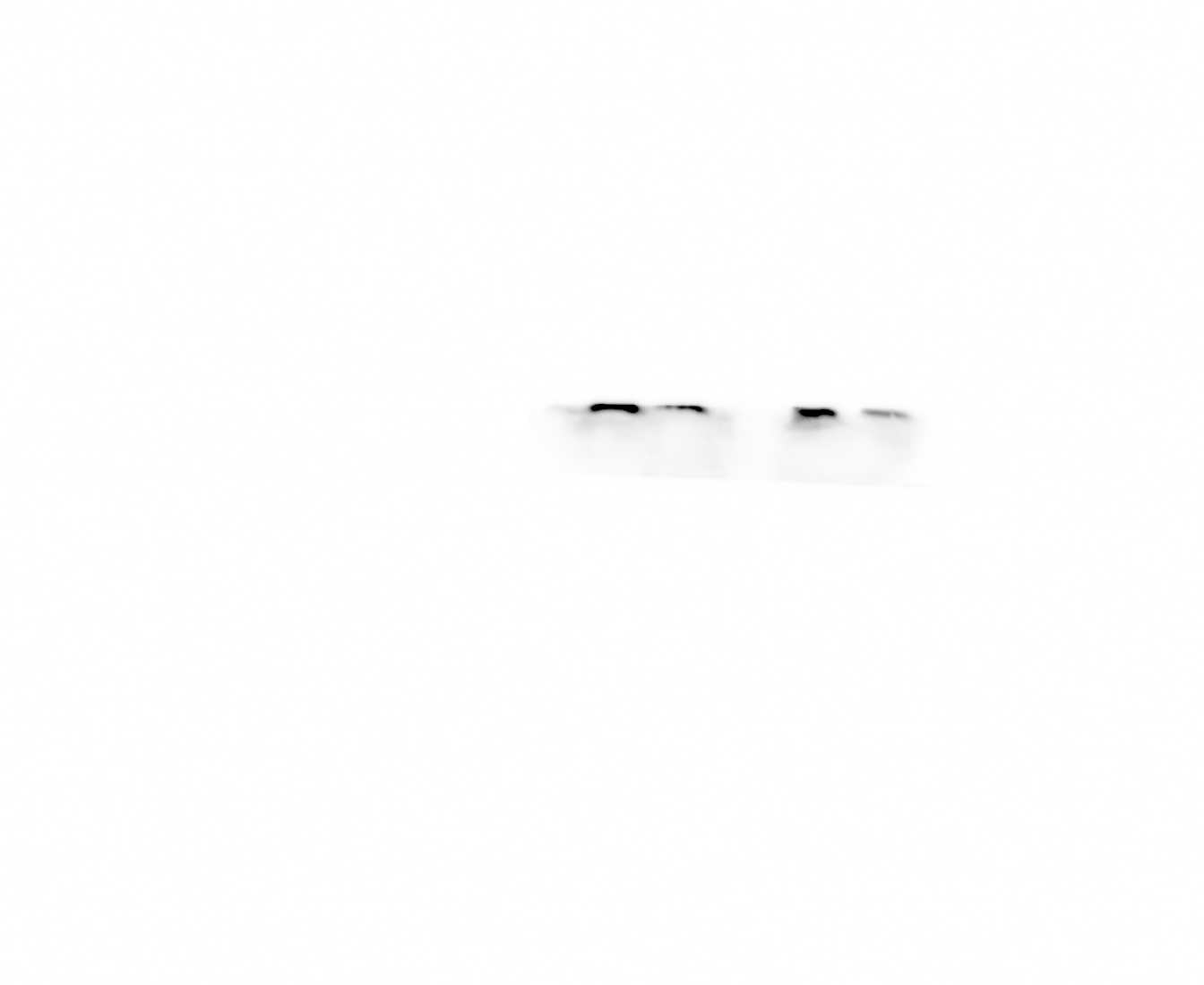

Supplement: Supplemental Information 5 [file peerj-11-15458-s005.zip › Fig.5/snail 04.png]

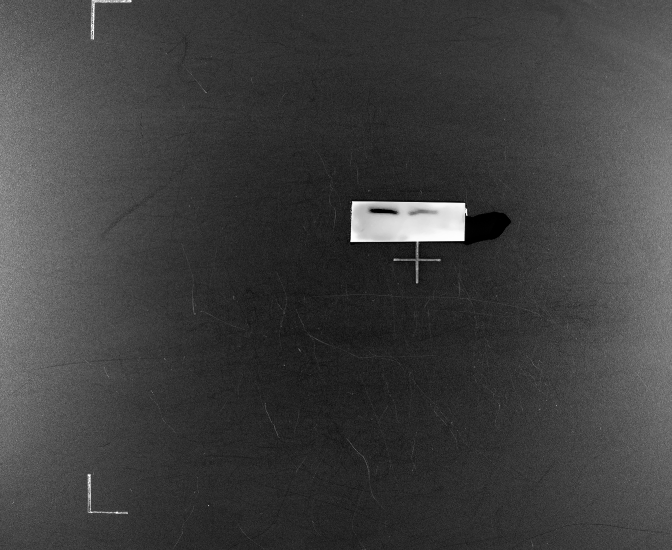

Supplement: Supplemental Information 5 [file peerj-11-15458-s005.zip › Fig.5/snail 05.png]

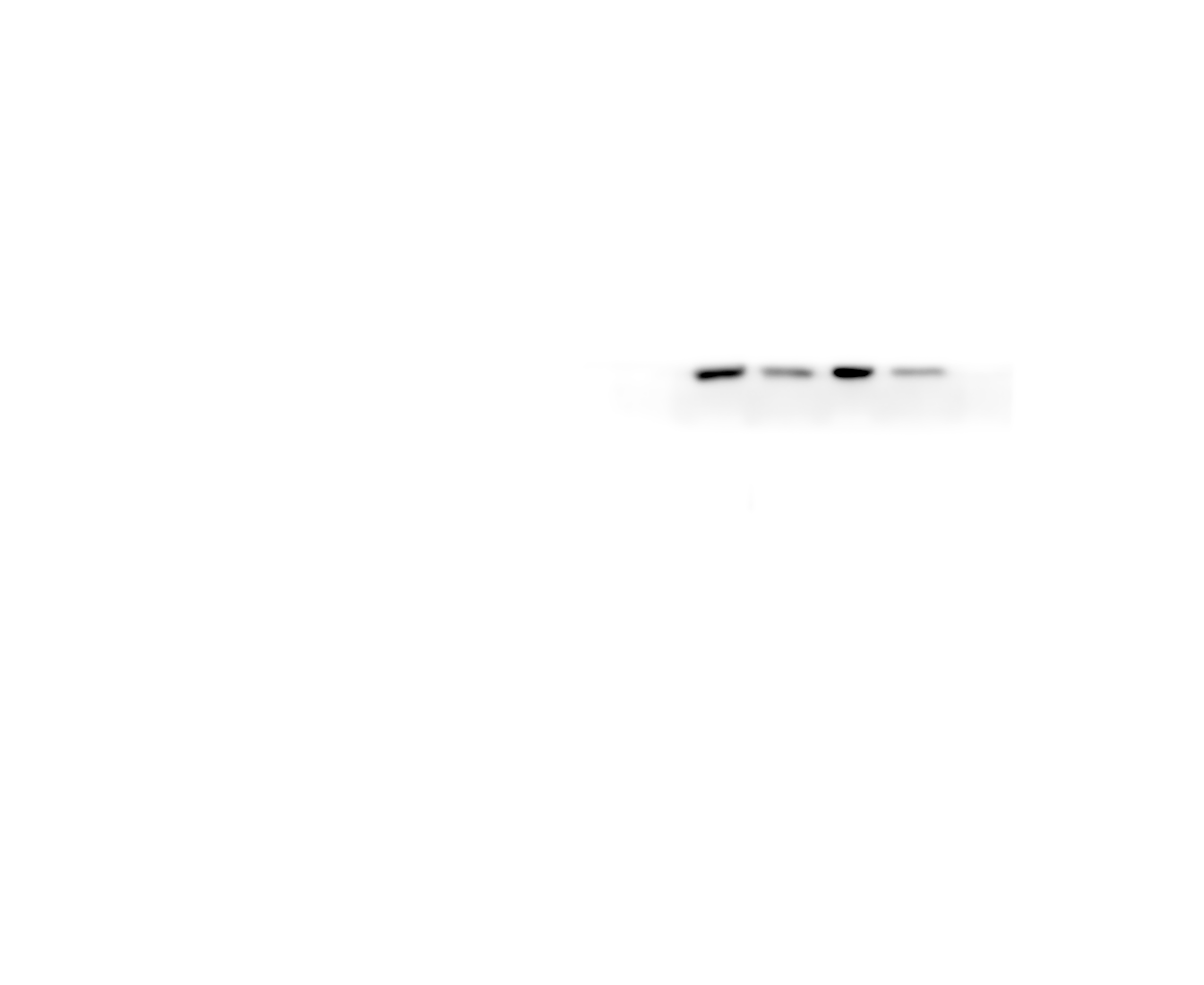

Supplement: Supplemental Information 5 [file peerj-11-15458-s005.zip › Fig.5/snail 02.png]

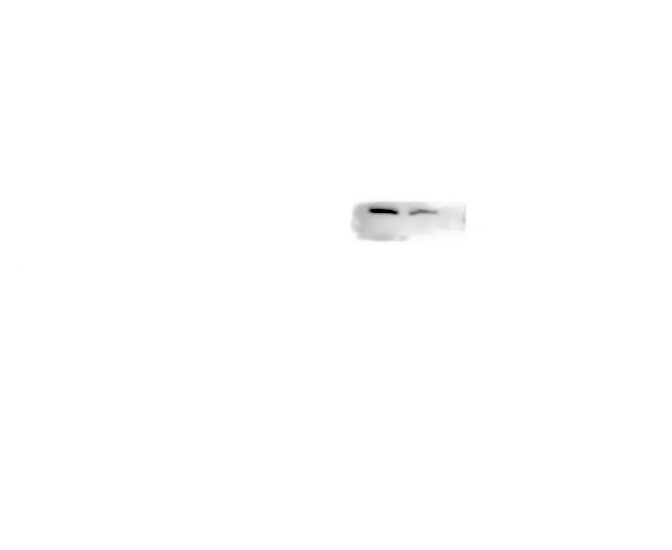

Supplement: Supplemental Information 5 [file peerj-11-15458-s005.zip › Fig.5/snail 06.png]

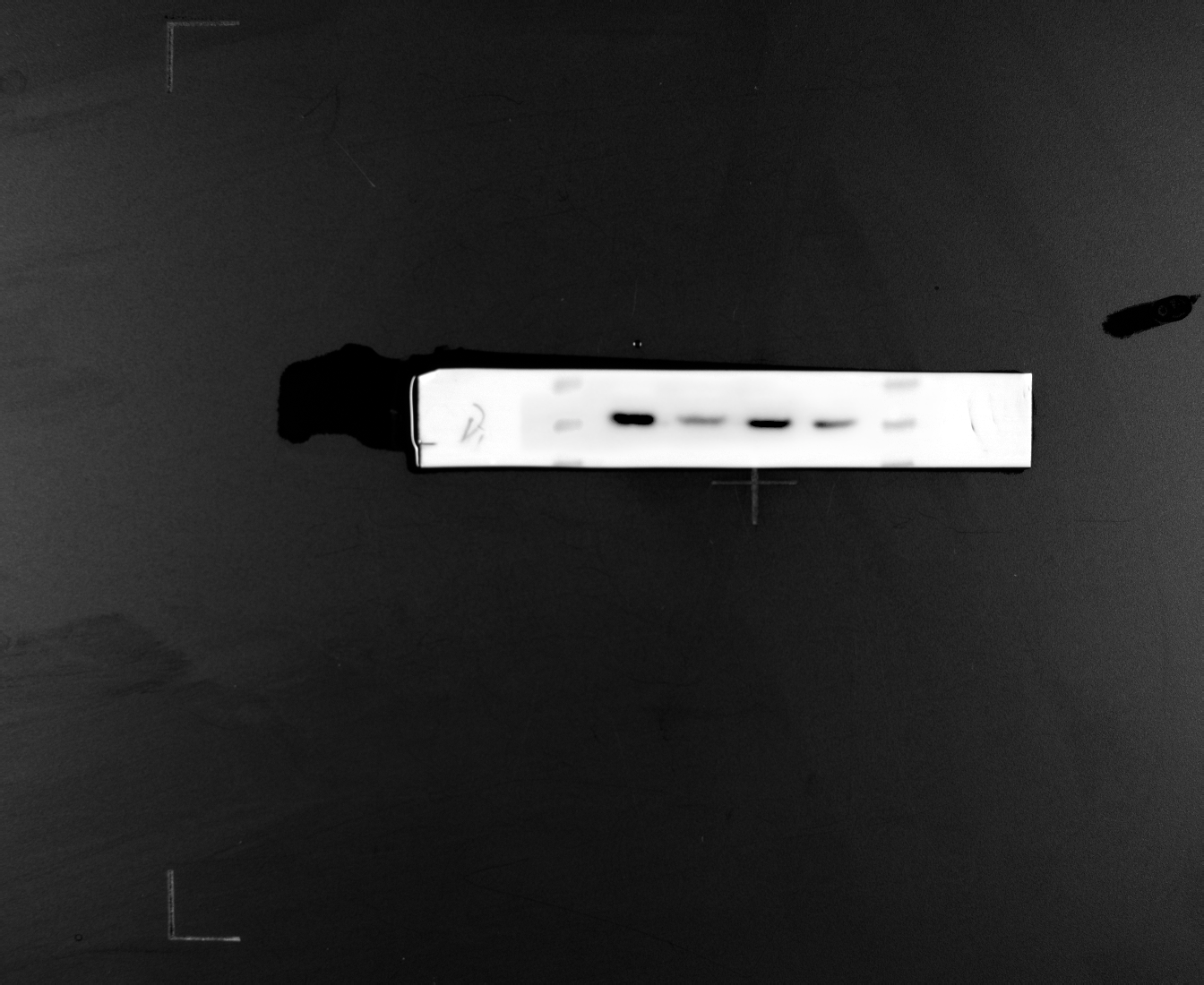

Supplement: Supplemental Information 6 [file peerj-11-15458-s006.zip › Fig.6/Cyclin D1 01.png]

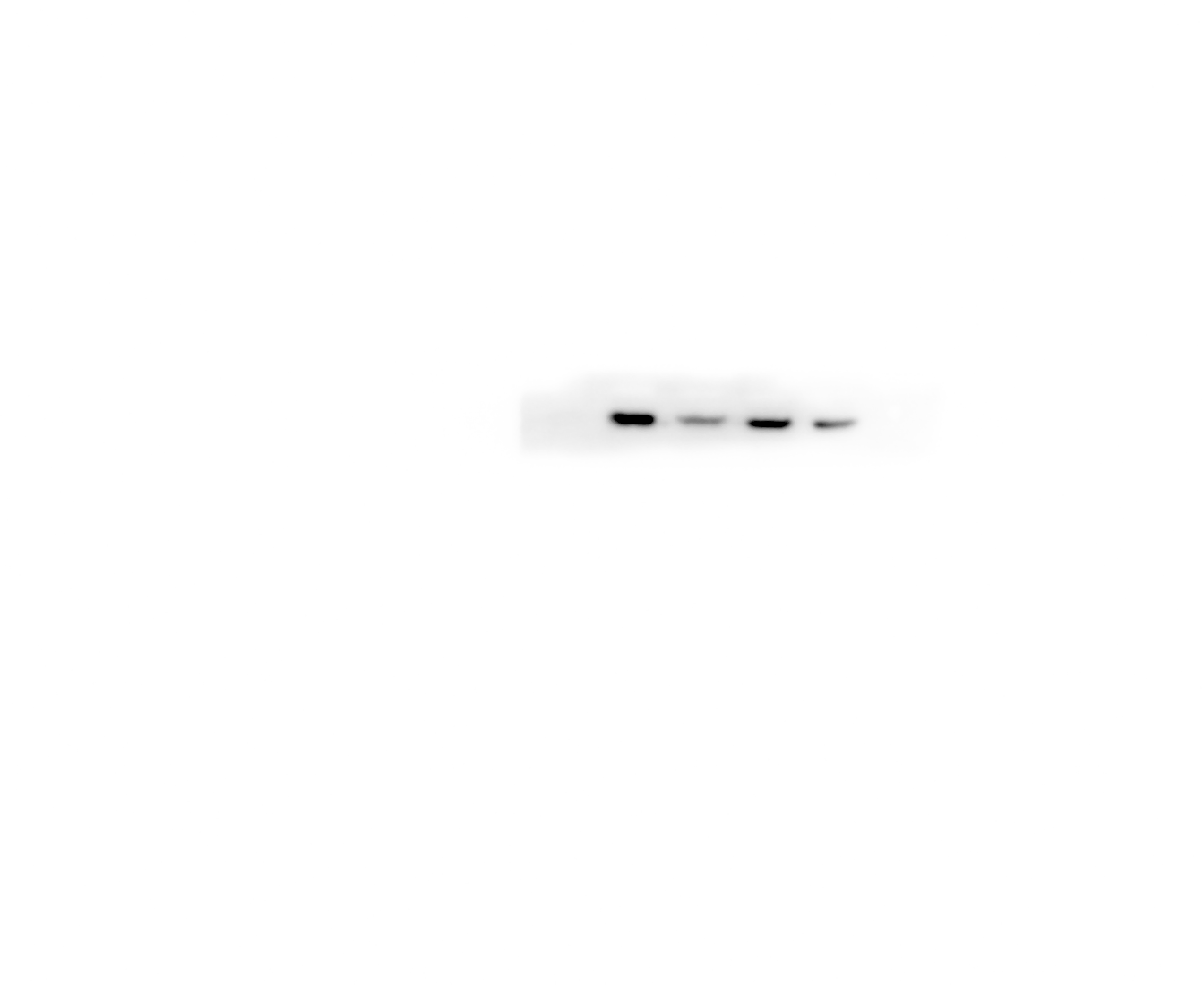

Supplement: Supplemental Information 6 [file peerj-11-15458-s006.zip › Fig.6/Cyclin D1 02.png]

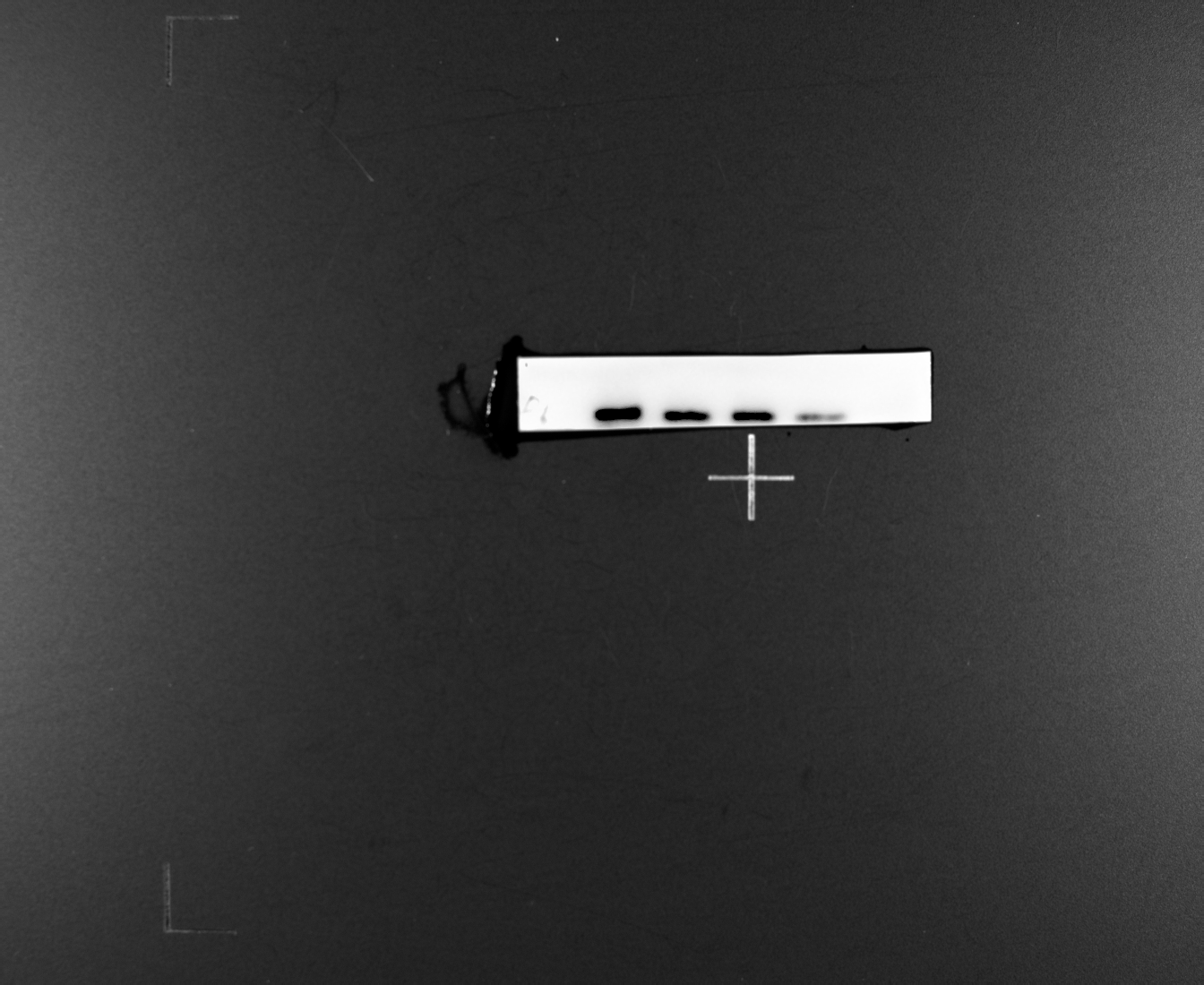

Supplement: Supplemental Information 6 [file peerj-11-15458-s006.zip › Fig.6/Cyclin D1 03.png]

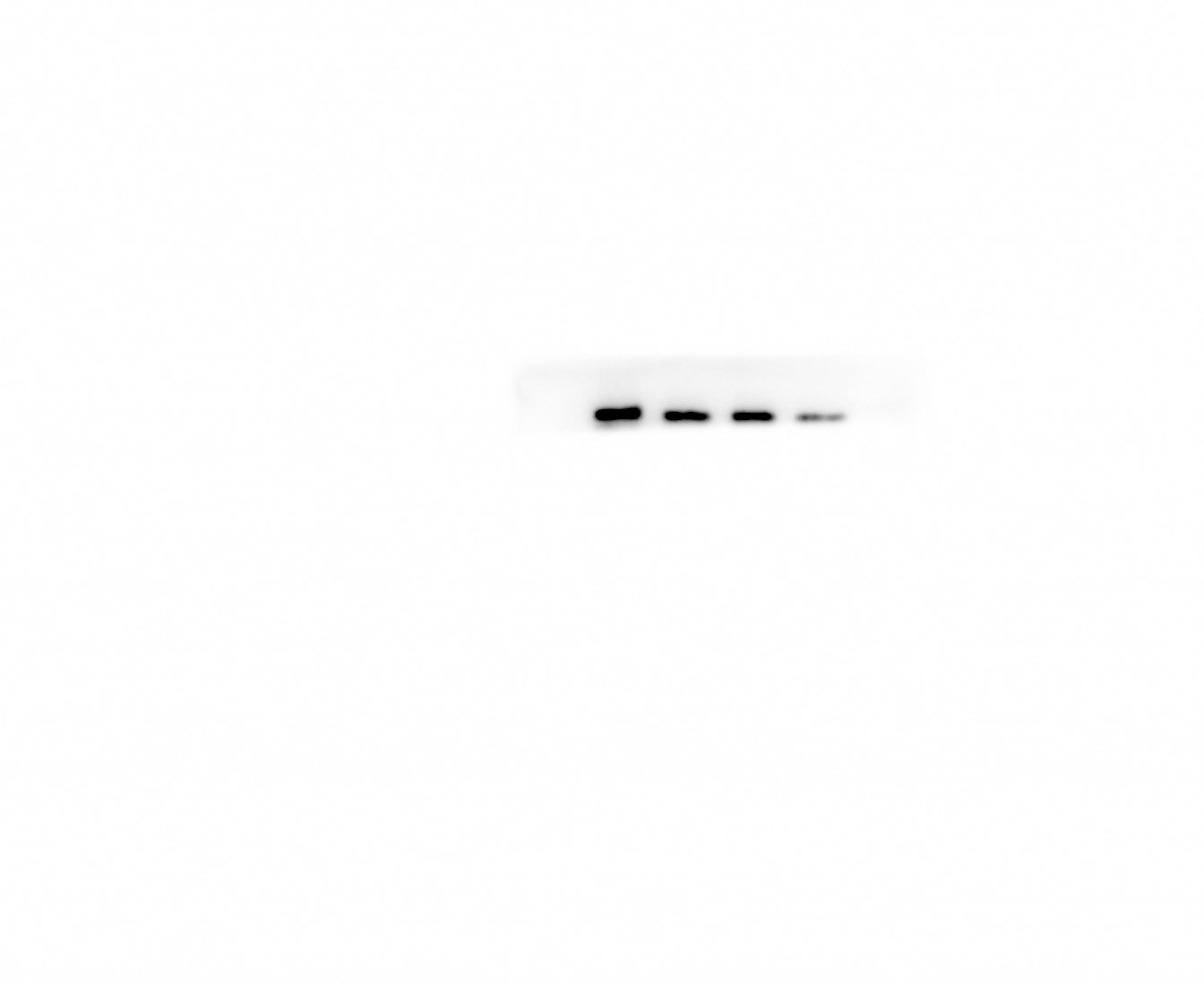

Supplement: Supplemental Information 6 [file peerj-11-15458-s006.zip › Fig.6/Cyclin D1 04.png]

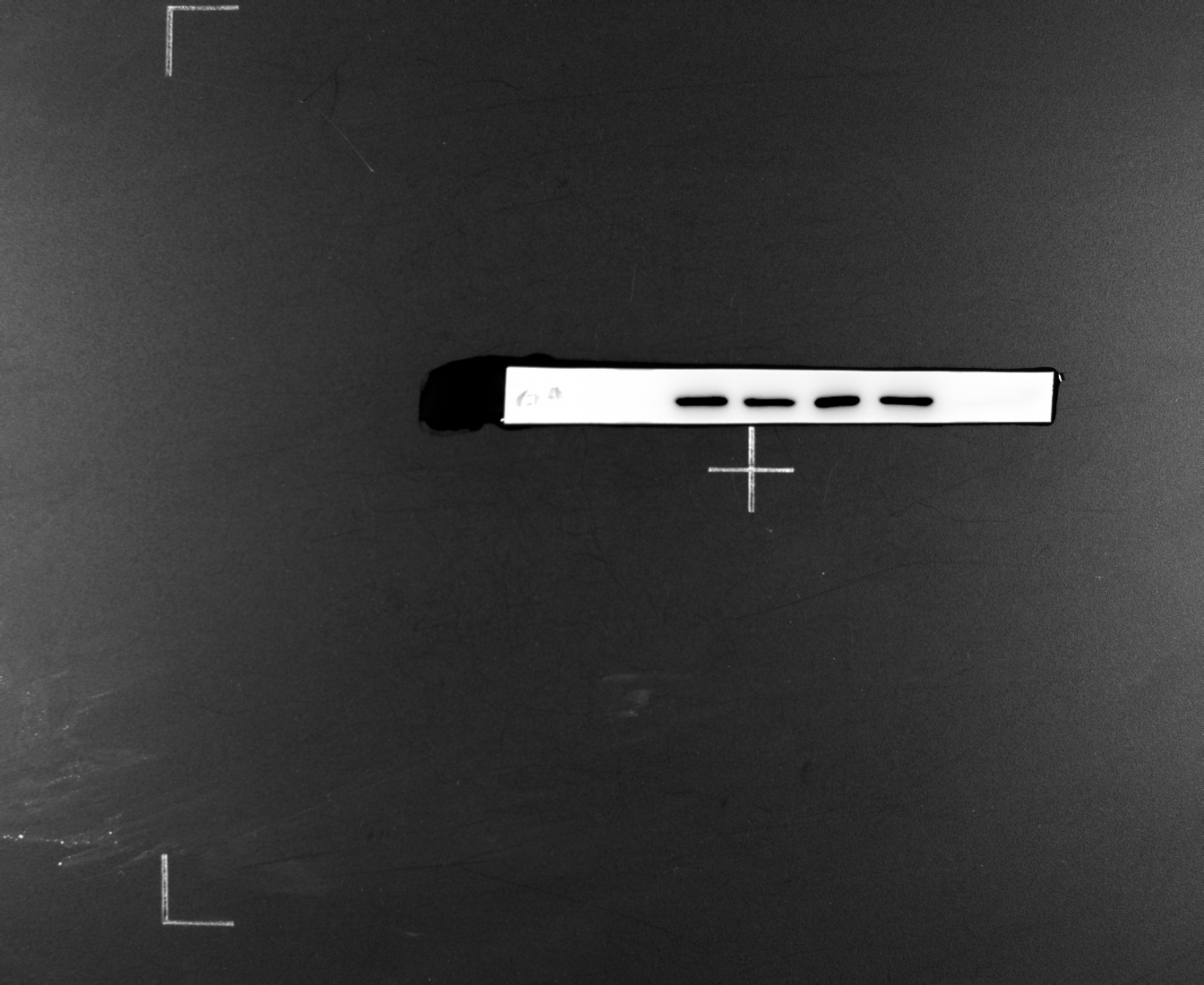

Supplement: Supplemental Information 6 [file peerj-11-15458-s006.zip › Fig.6/GAPDH 01.png]

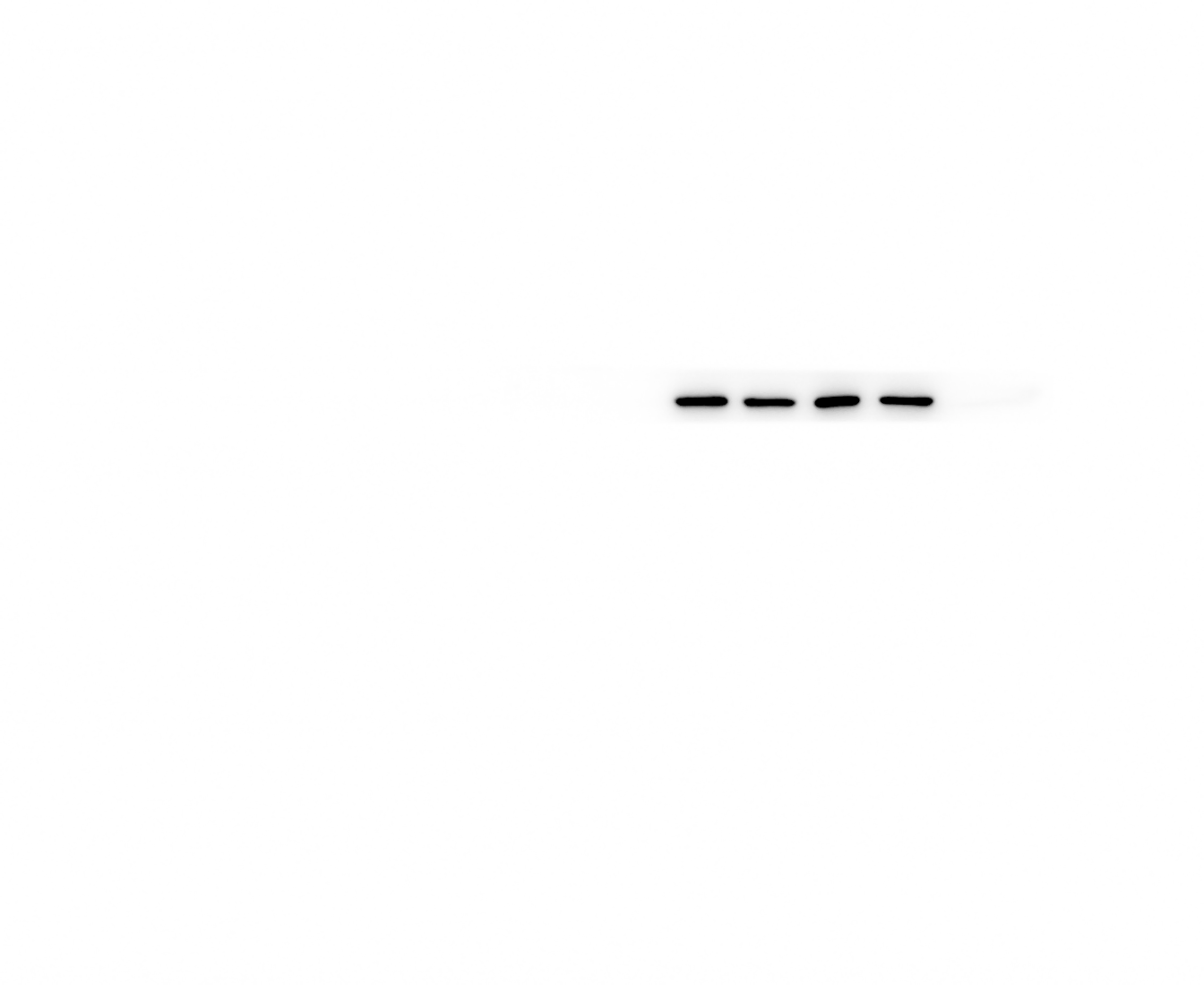

Supplement: Supplemental Information 6 [file peerj-11-15458-s006.zip › Fig.6/GAPDH 02.png]

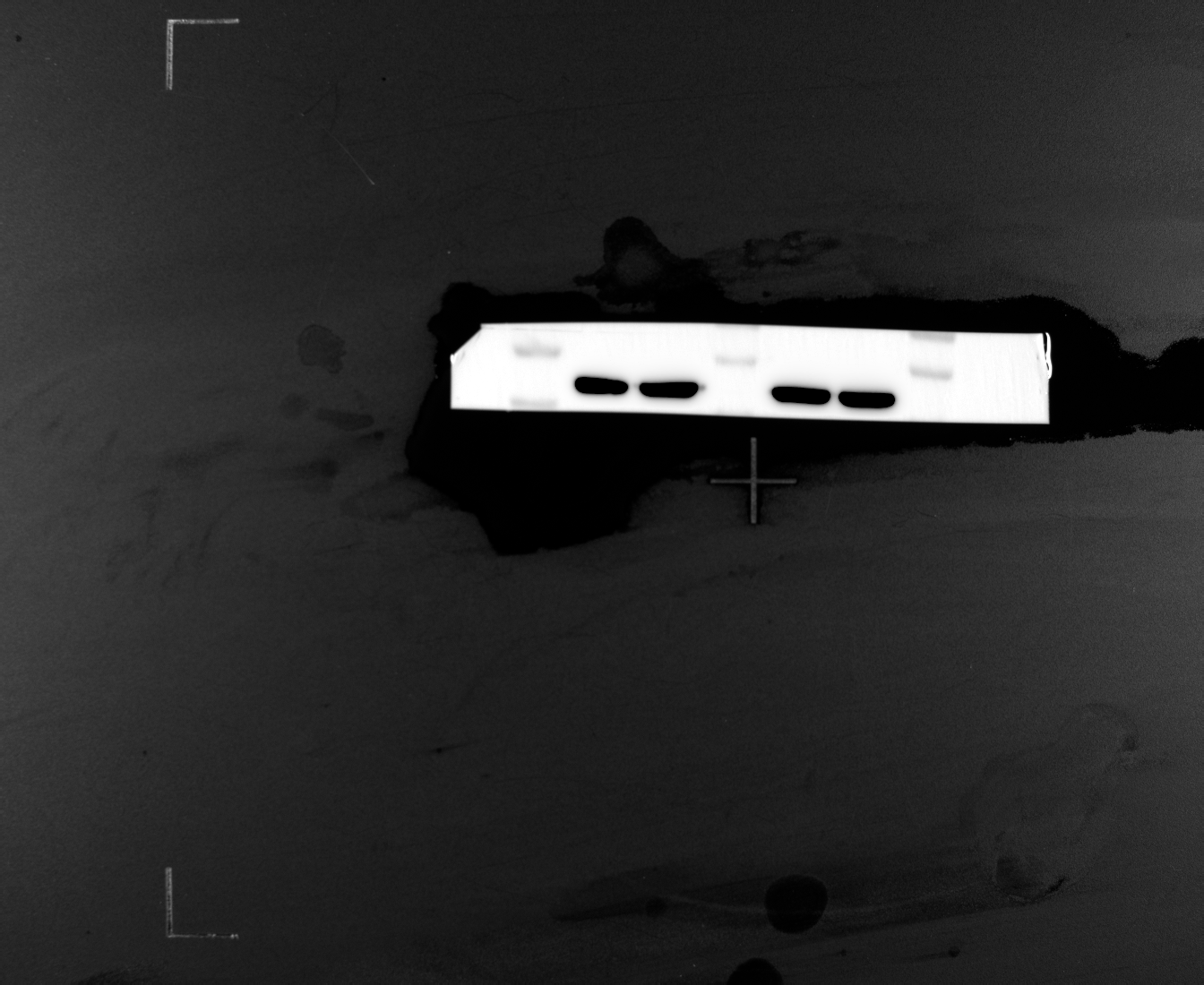

Supplement: Supplemental Information 6 [file peerj-11-15458-s006.zip › Fig.6/GAPDH 03.png]

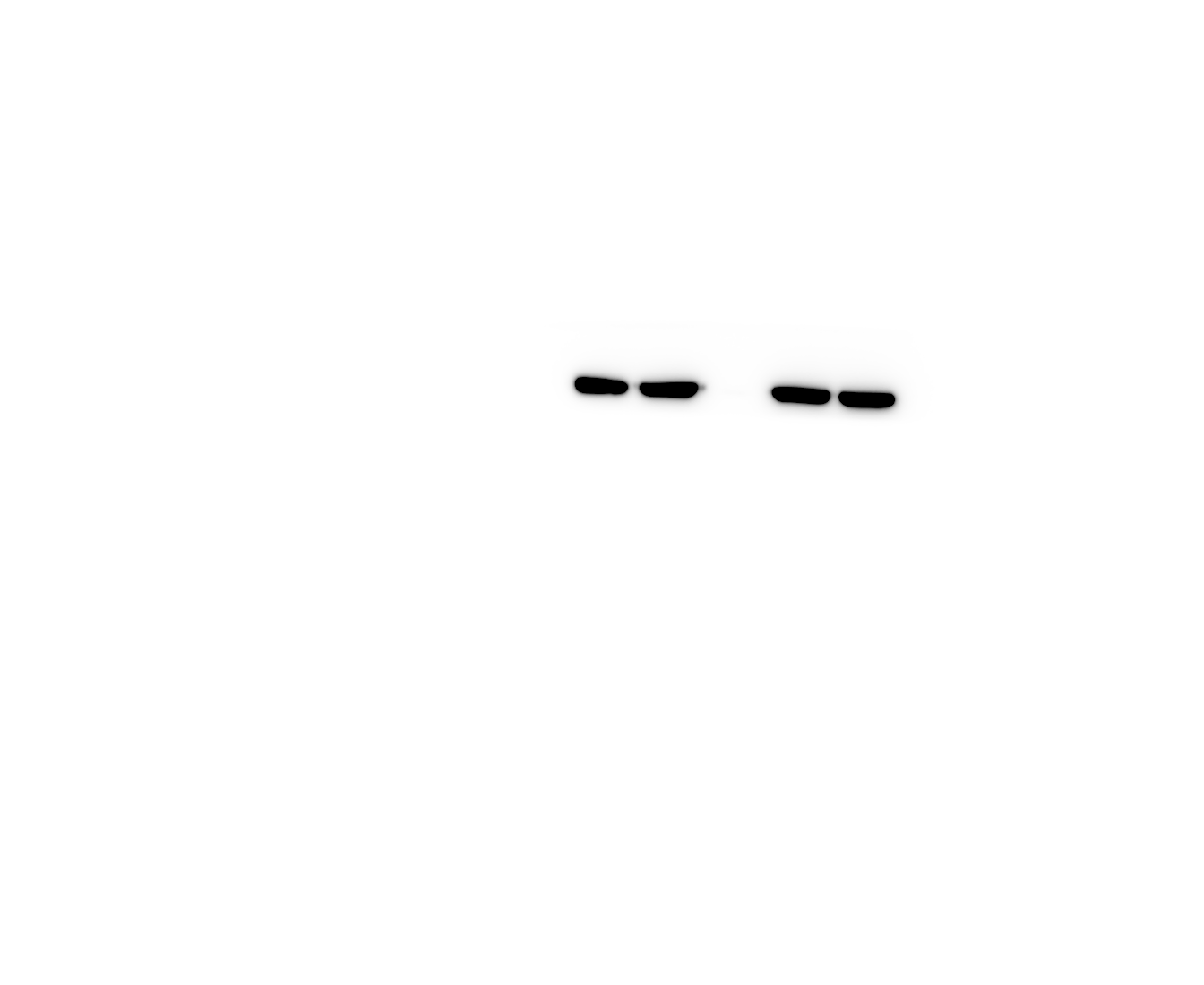

Supplement: Supplemental Information 6 [file peerj-11-15458-s006.zip › Fig.6/GAPDH 04.png]

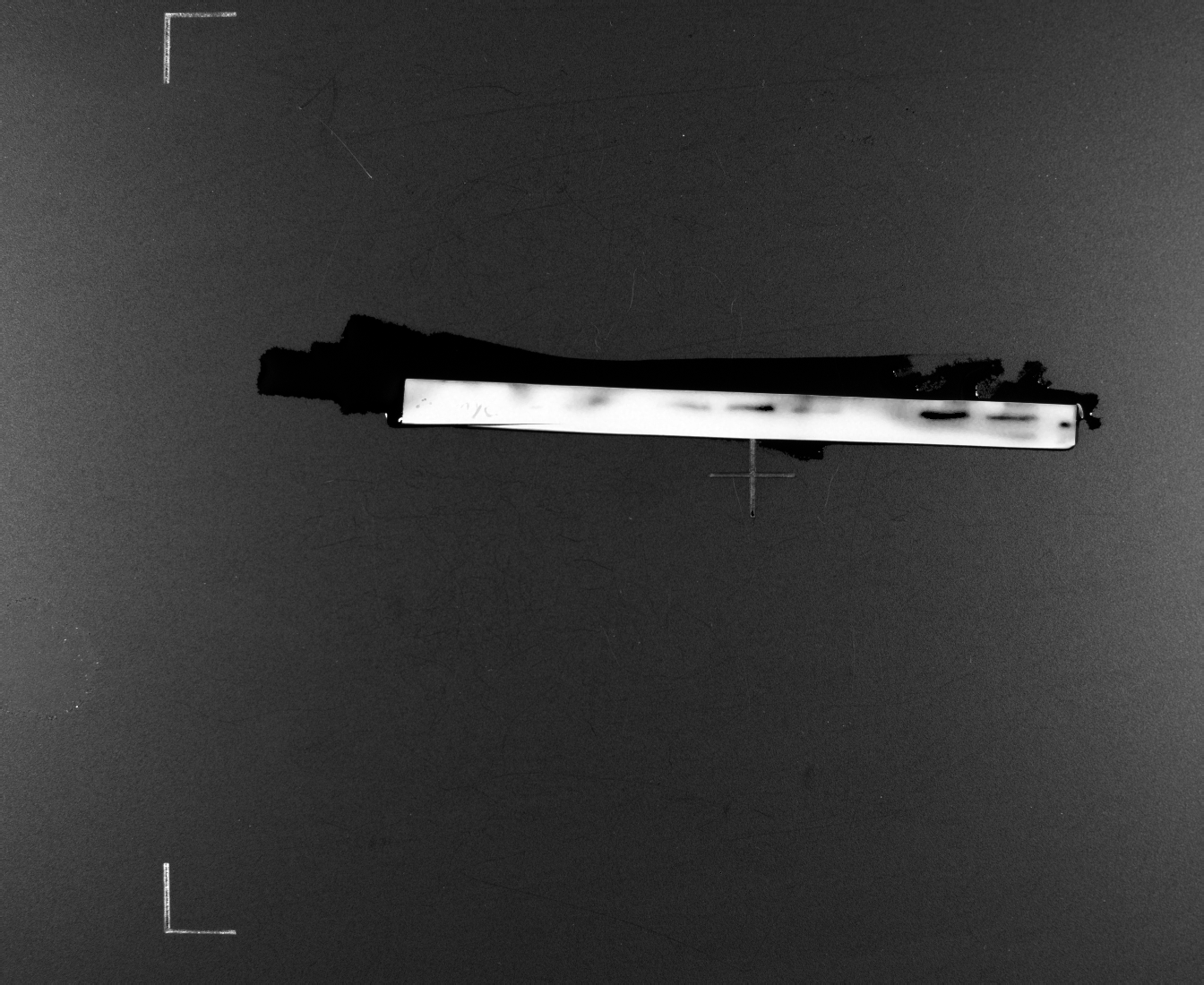

Supplement: Supplemental Information 6 [file peerj-11-15458-s006.zip › Fig.6/c-myc 01.png]

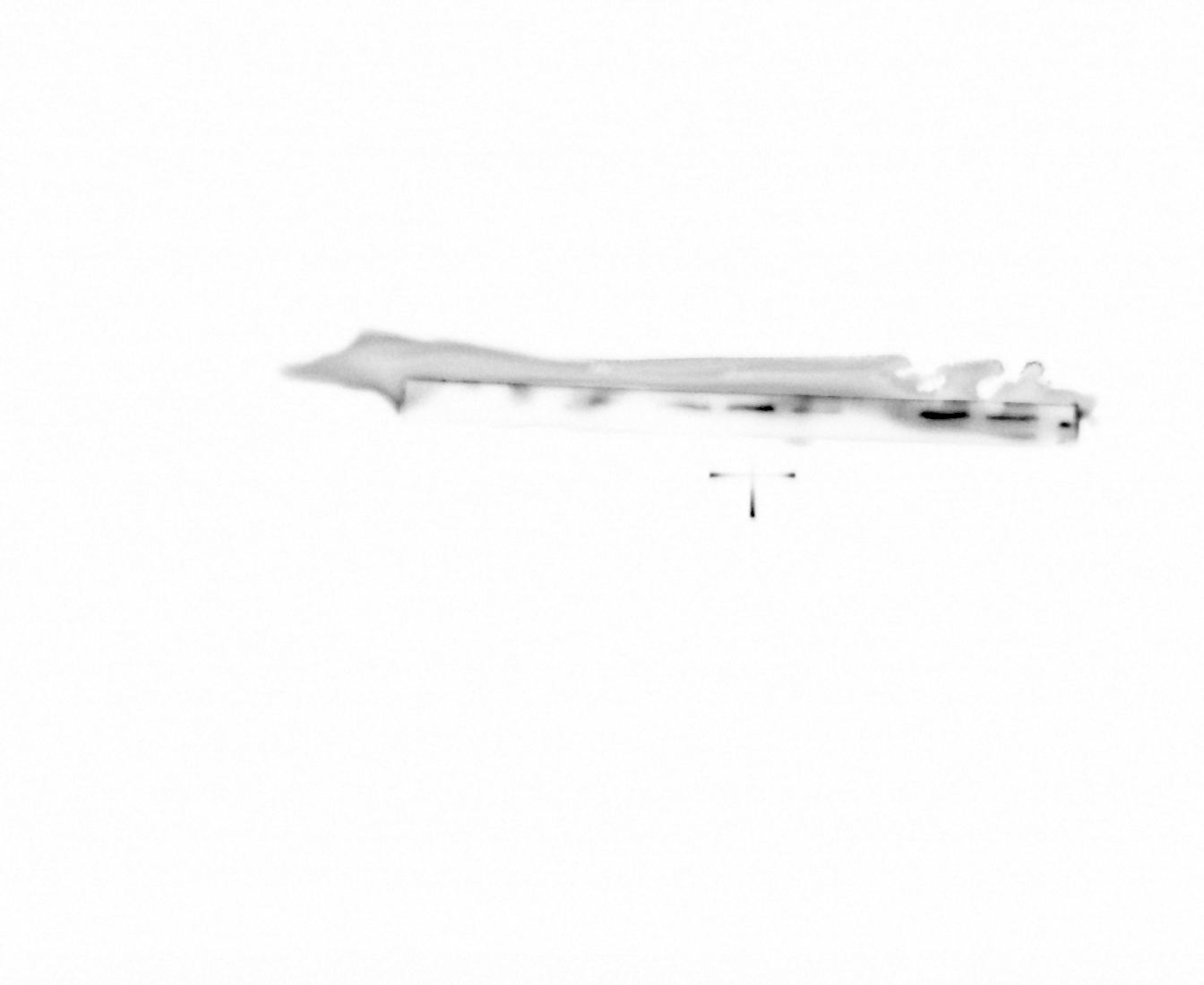

Supplement: Supplemental Information 6 [file peerj-11-15458-s006.zip › Fig.6/c-myc 02.png]

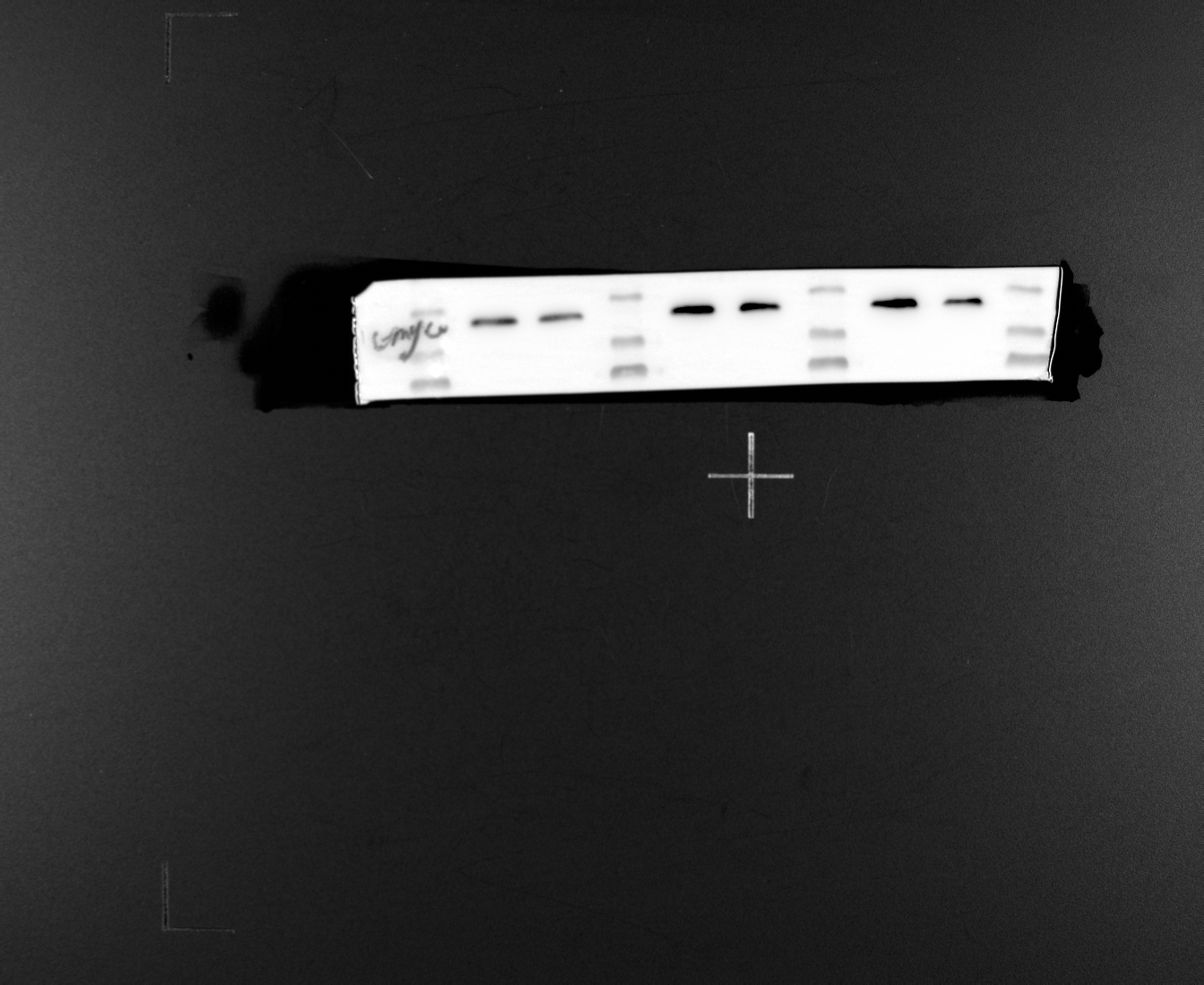

Supplement: Supplemental Information 6 [file peerj-11-15458-s006.zip › Fig.6/c-myc 03.png]

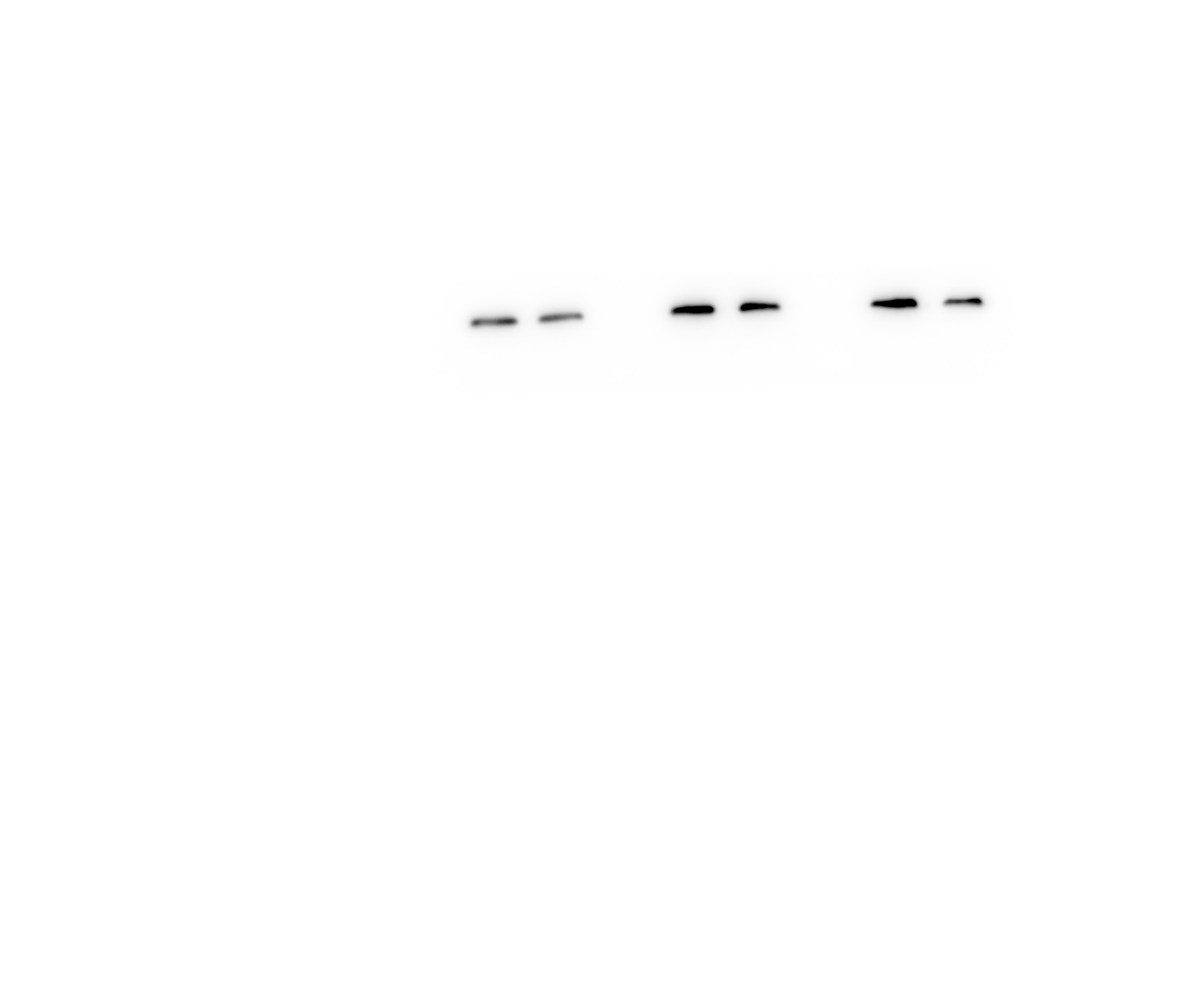

Supplement: Supplemental Information 6 [file peerj-11-15458-s006.zip › Fig.6/c-myc 04.png]
