# Supplementary figures and images for: CTHRC1 promotes anaplastic thyroid cancer progression by upregulating the proliferation, migration, and invasion of tumor cells (part 2 of 2)
Source: PeerJ. 2023 May 29;11:e15458. doi: 10.7717/peerj.15458 (PMC10234271; doi:10.7717/peerj.15458)

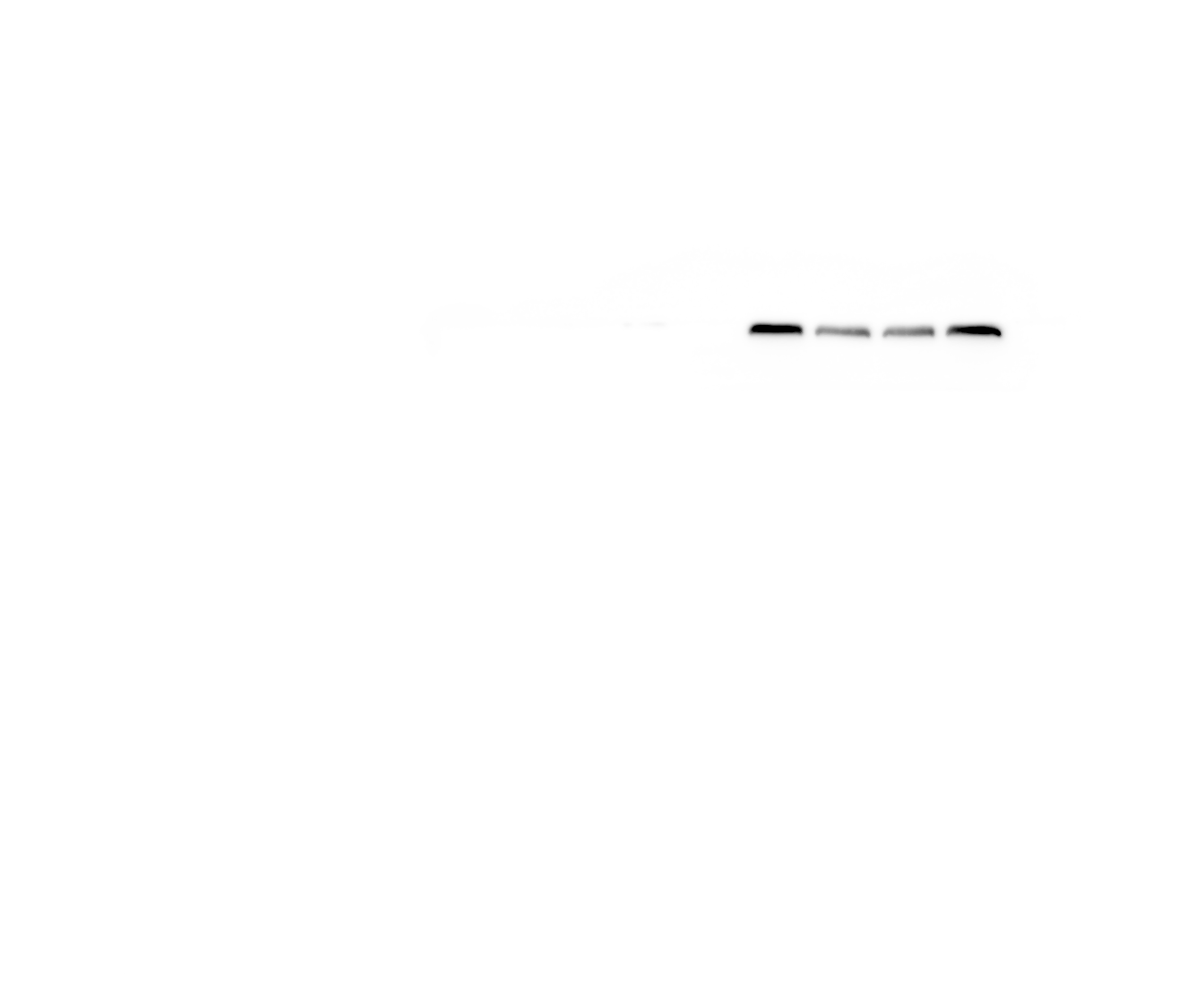

Supplement: Supplemental Information 6 [file peerj-11-15458-s006.zip › Fig.6/c-myc 05.png]

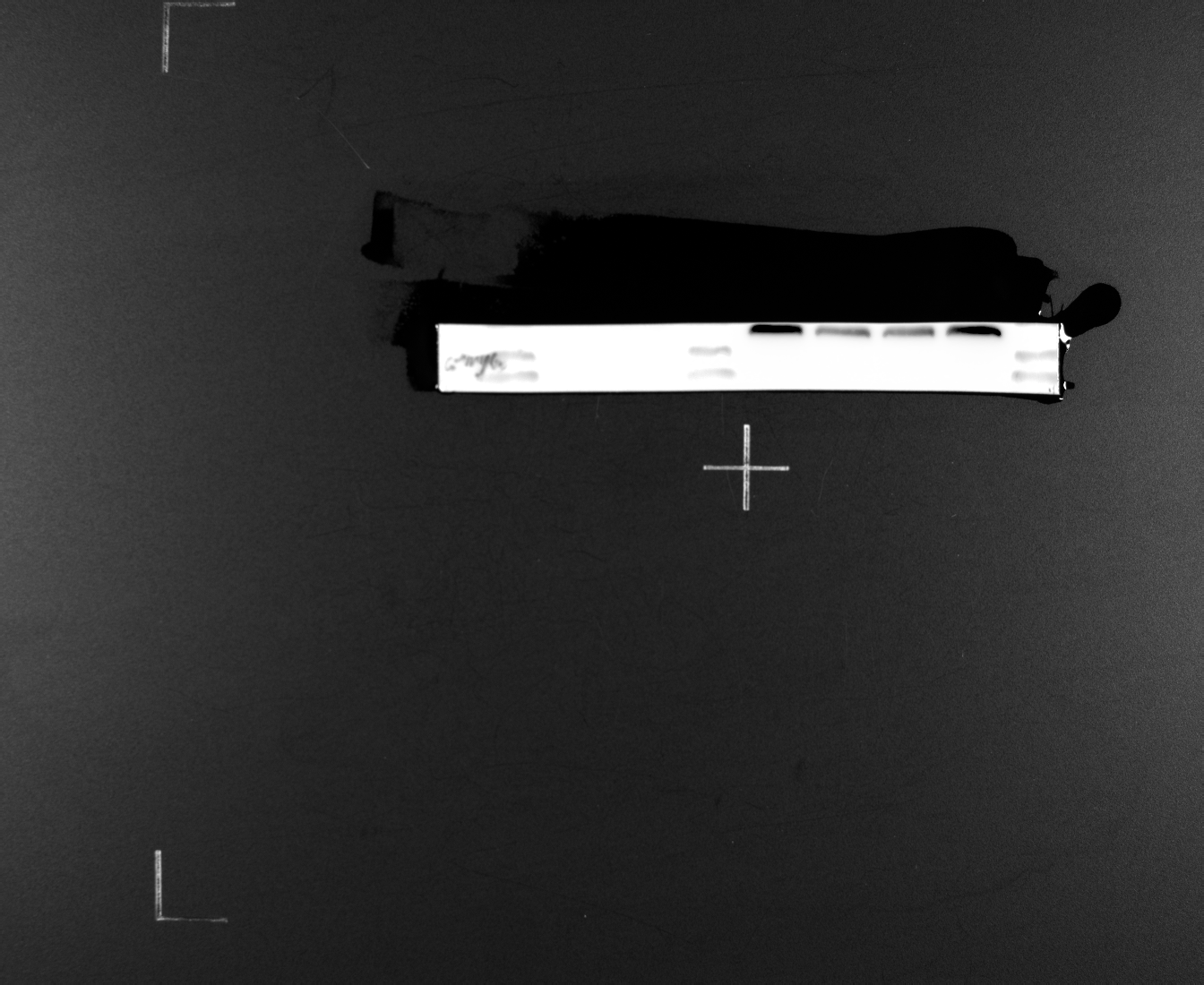

Supplement: Supplemental Information 6 [file peerj-11-15458-s006.zip › Fig.6/c-myc 06.png]

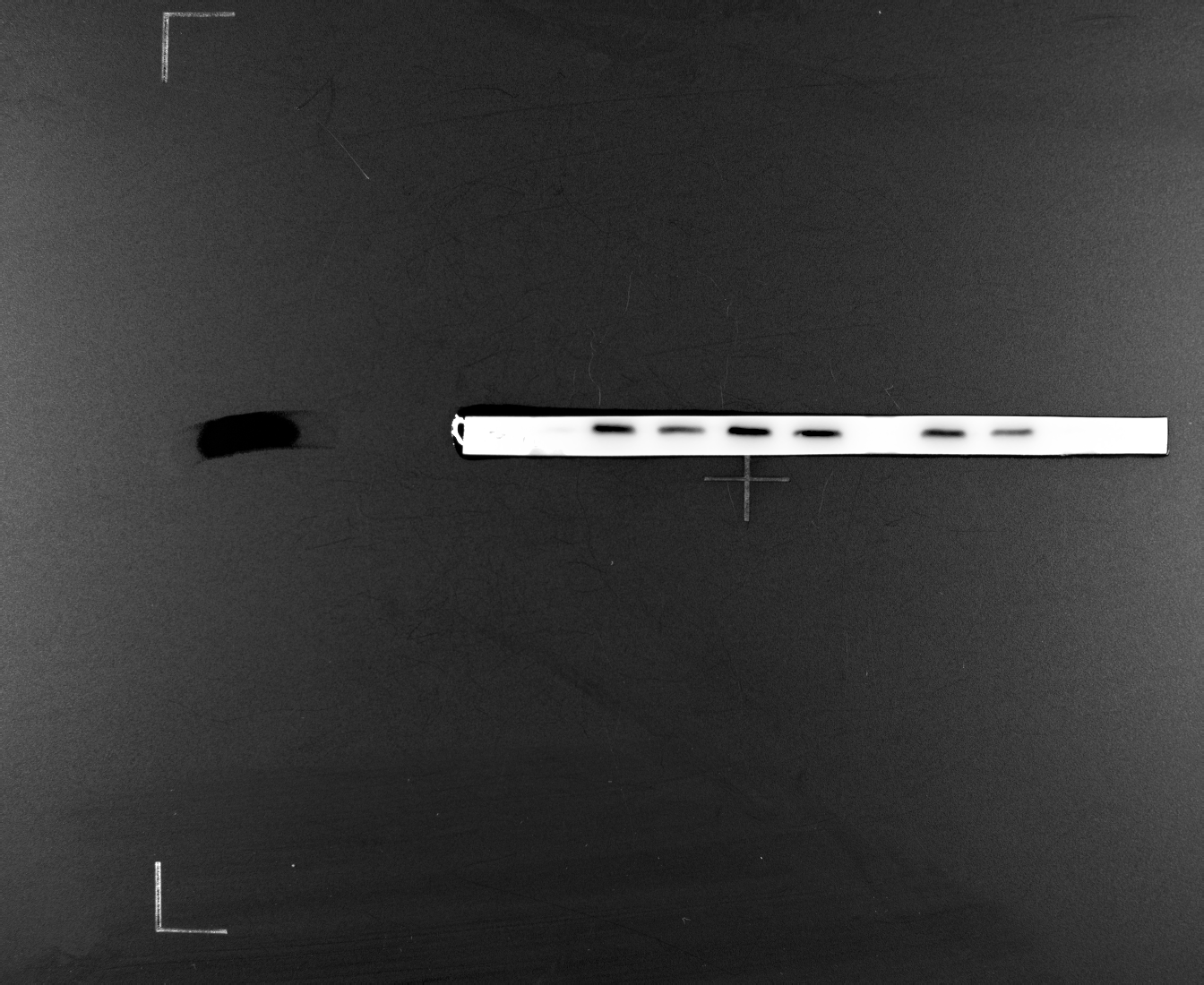

Supplement: Supplemental Information 6 [file peerj-11-15458-s006.zip › Fig.6/c-myc 07.png]

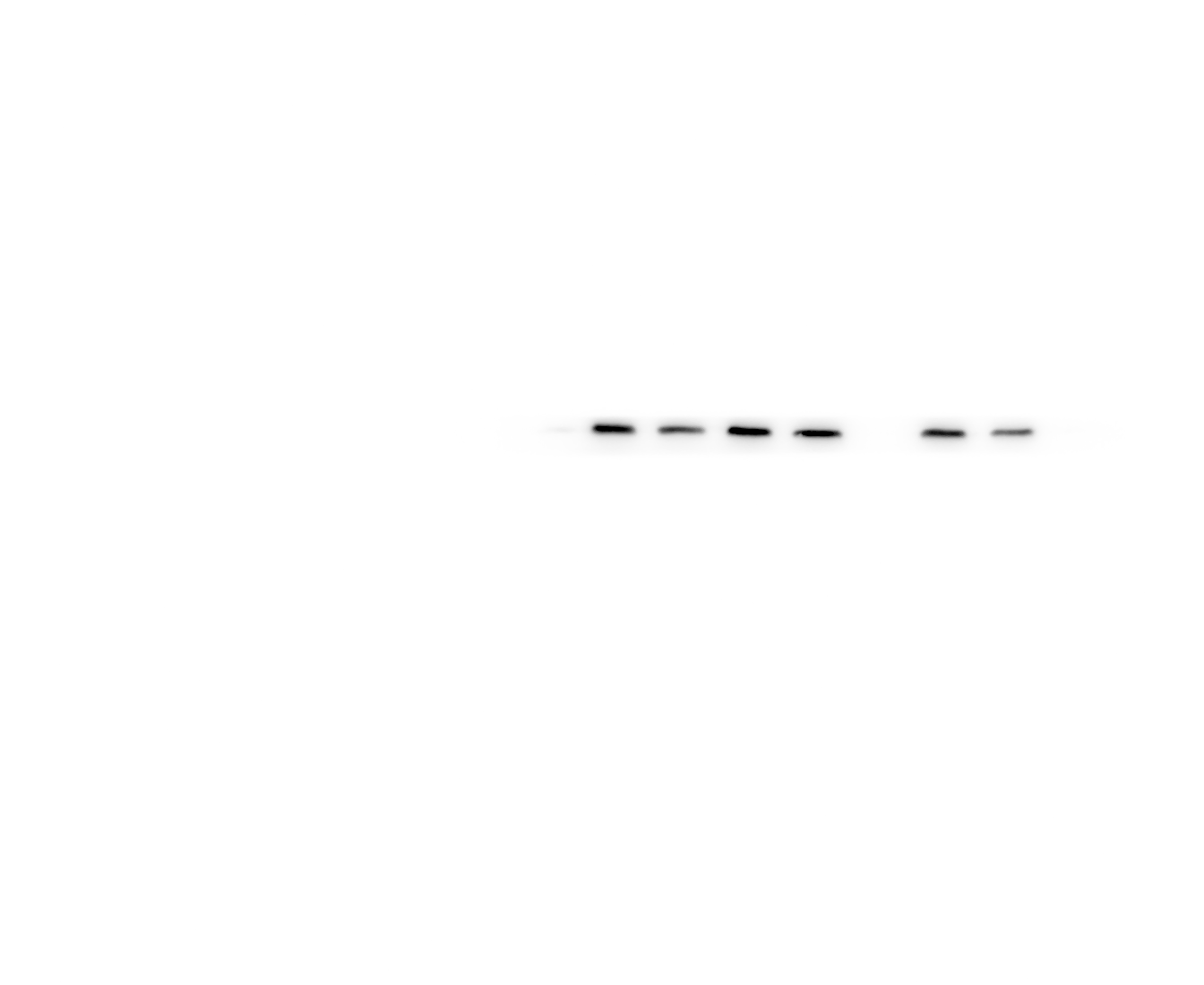

Supplement: Supplemental Information 6 [file peerj-11-15458-s006.zip › Fig.6/c-myc 08.png]

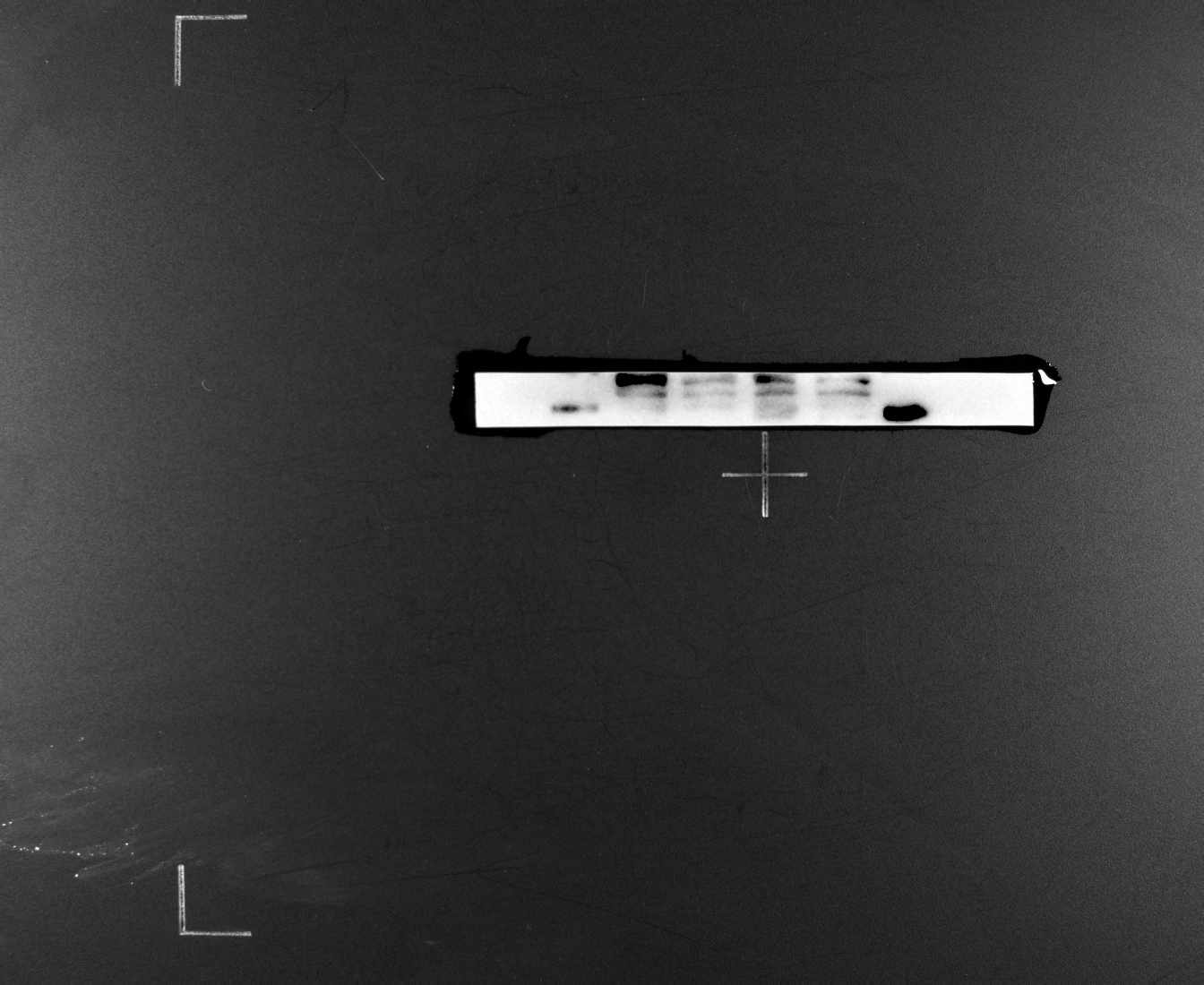

Supplement: Supplemental Information 6 [file peerj-11-15458-s006.zip › Fig.6/a┬-catenin 01.png]

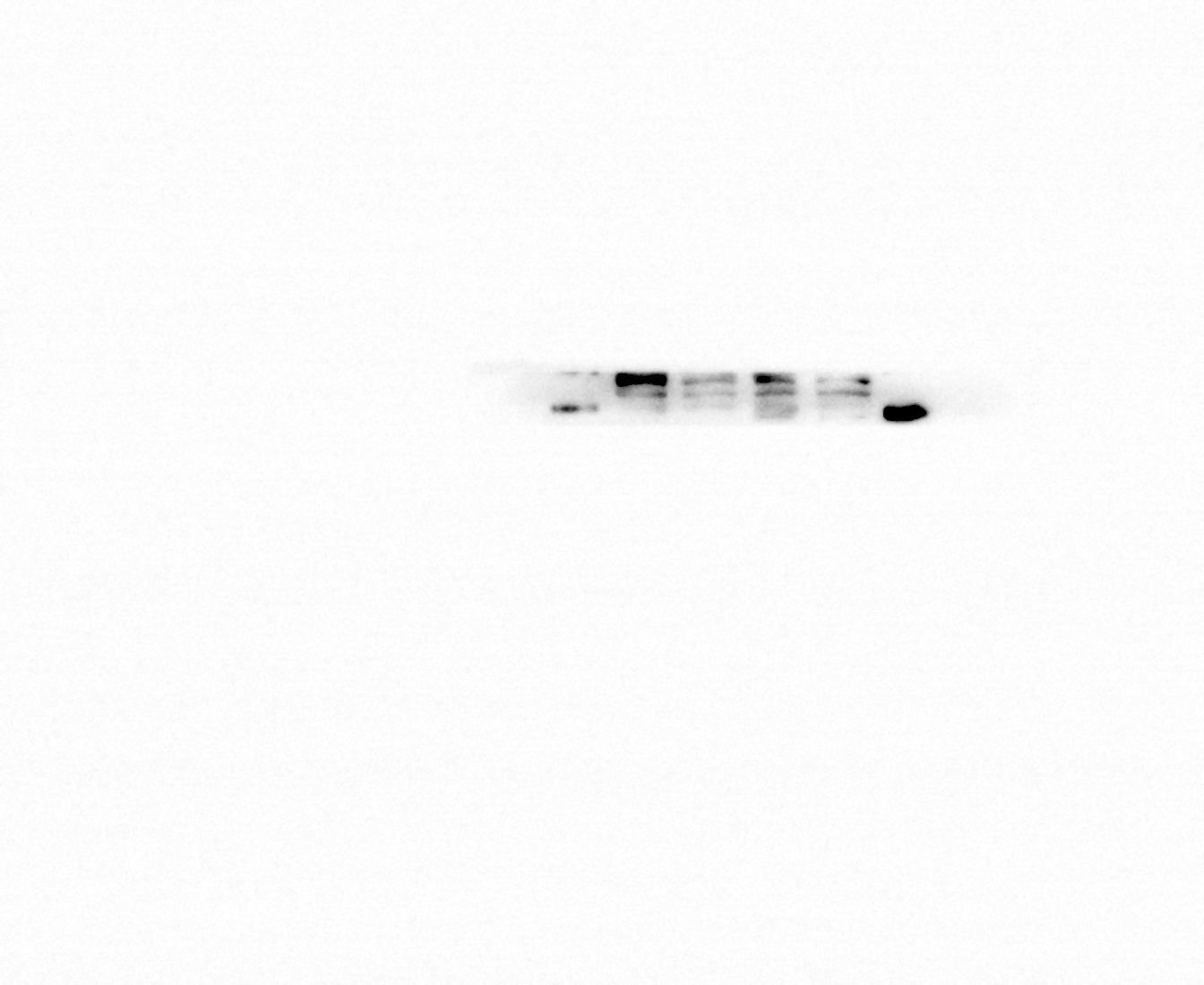

Supplement: Supplemental Information 6 [file peerj-11-15458-s006.zip › Fig.6/a┬-catenin 02.png]

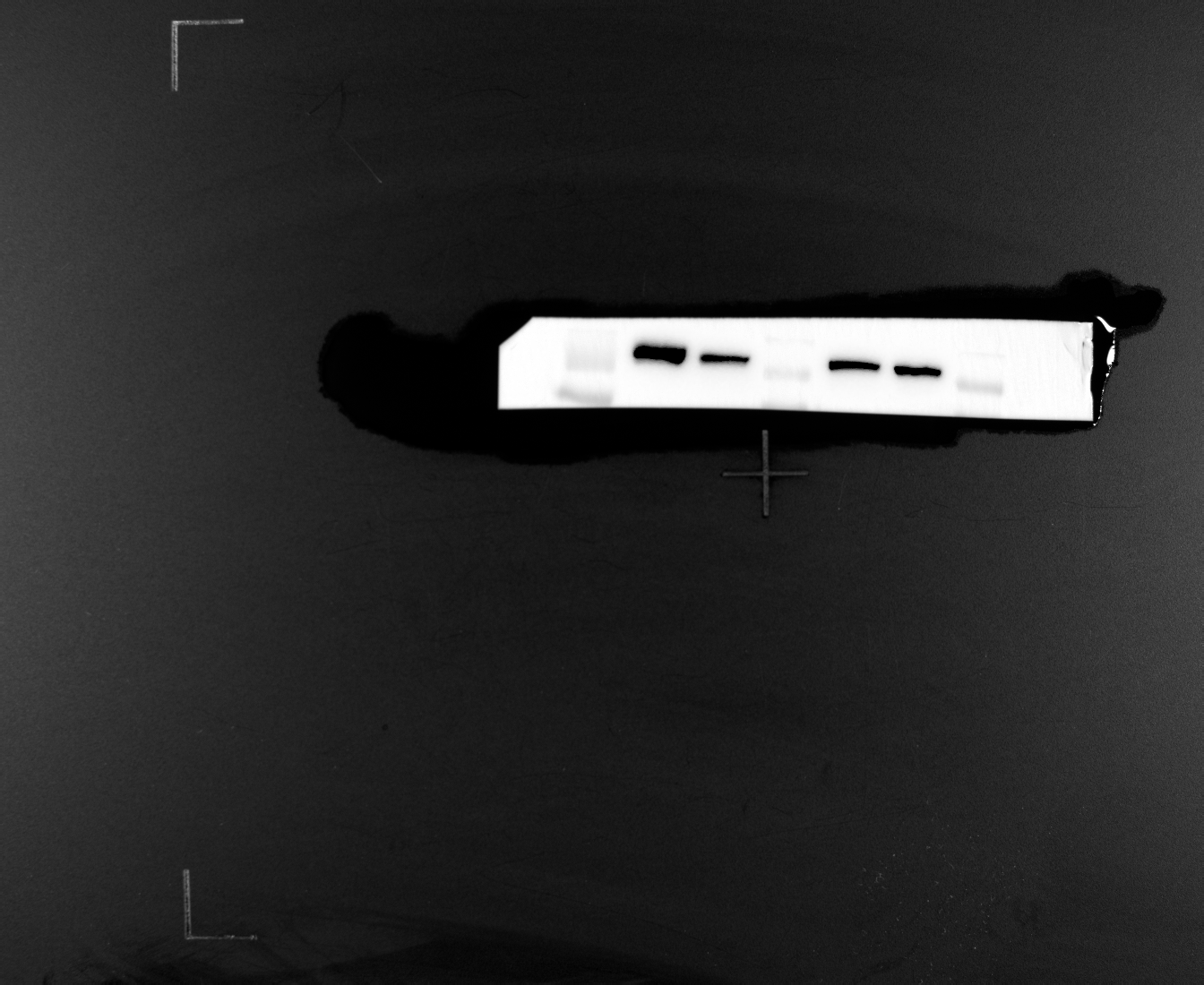

Supplement: Supplemental Information 6 [file peerj-11-15458-s006.zip › Fig.6/a┬-catenin 03.png]

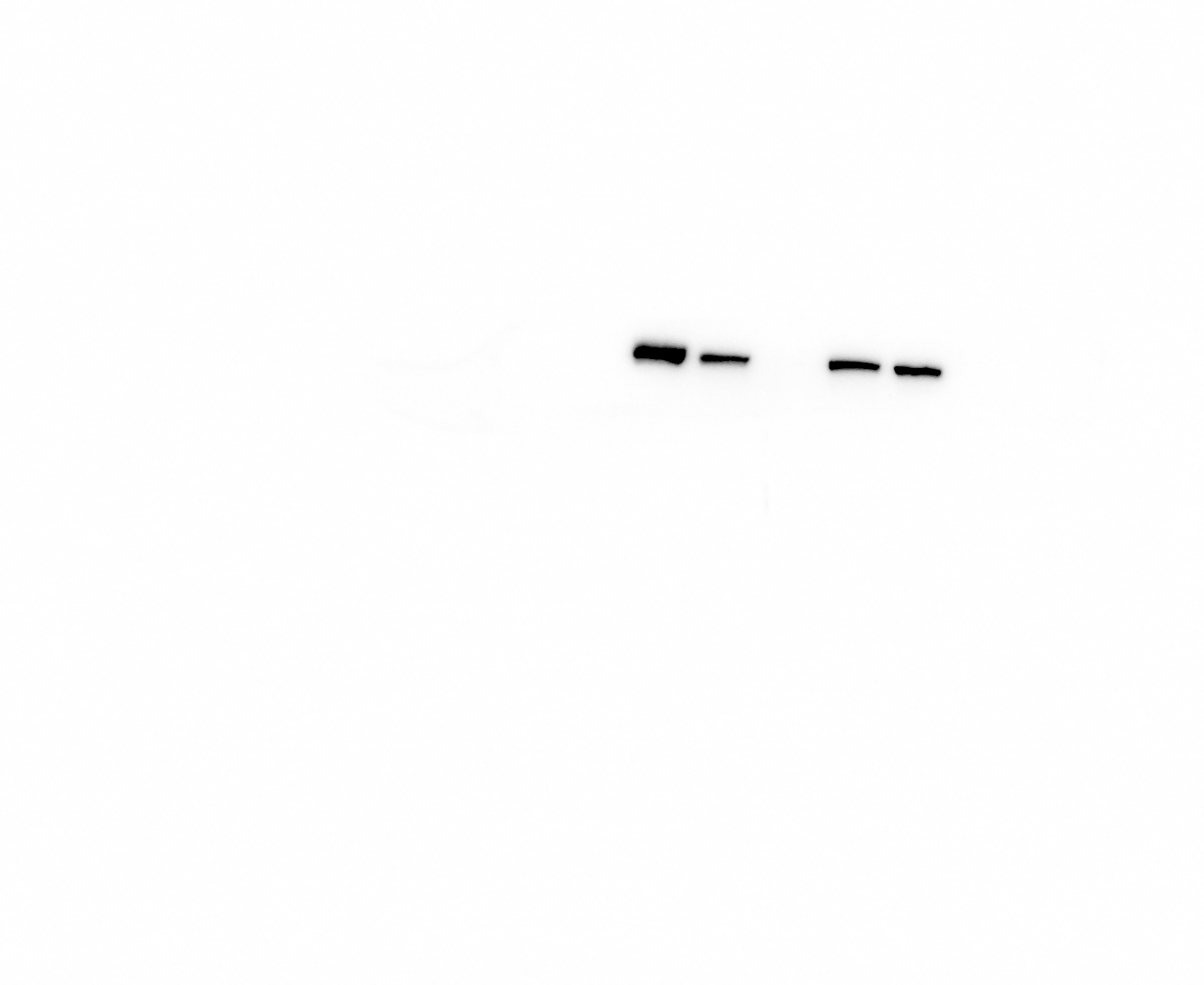

Supplement: Supplemental Information 6 [file peerj-11-15458-s006.zip › Fig.6/a┬-catenin 04.png]

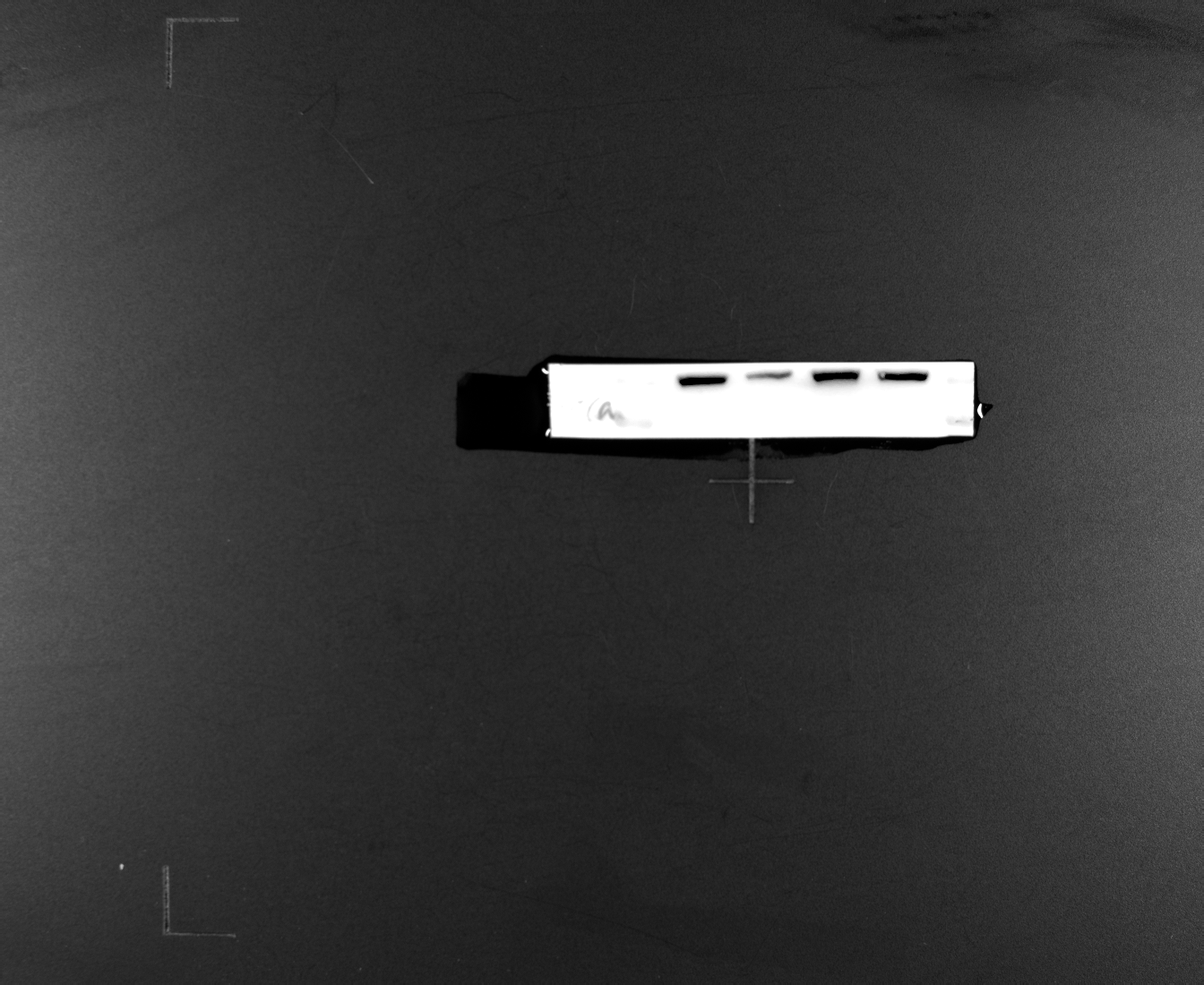

Supplement: Supplemental Information 6 [file peerj-11-15458-s006.zip › Fig.6/a┬-catenin 05.png]

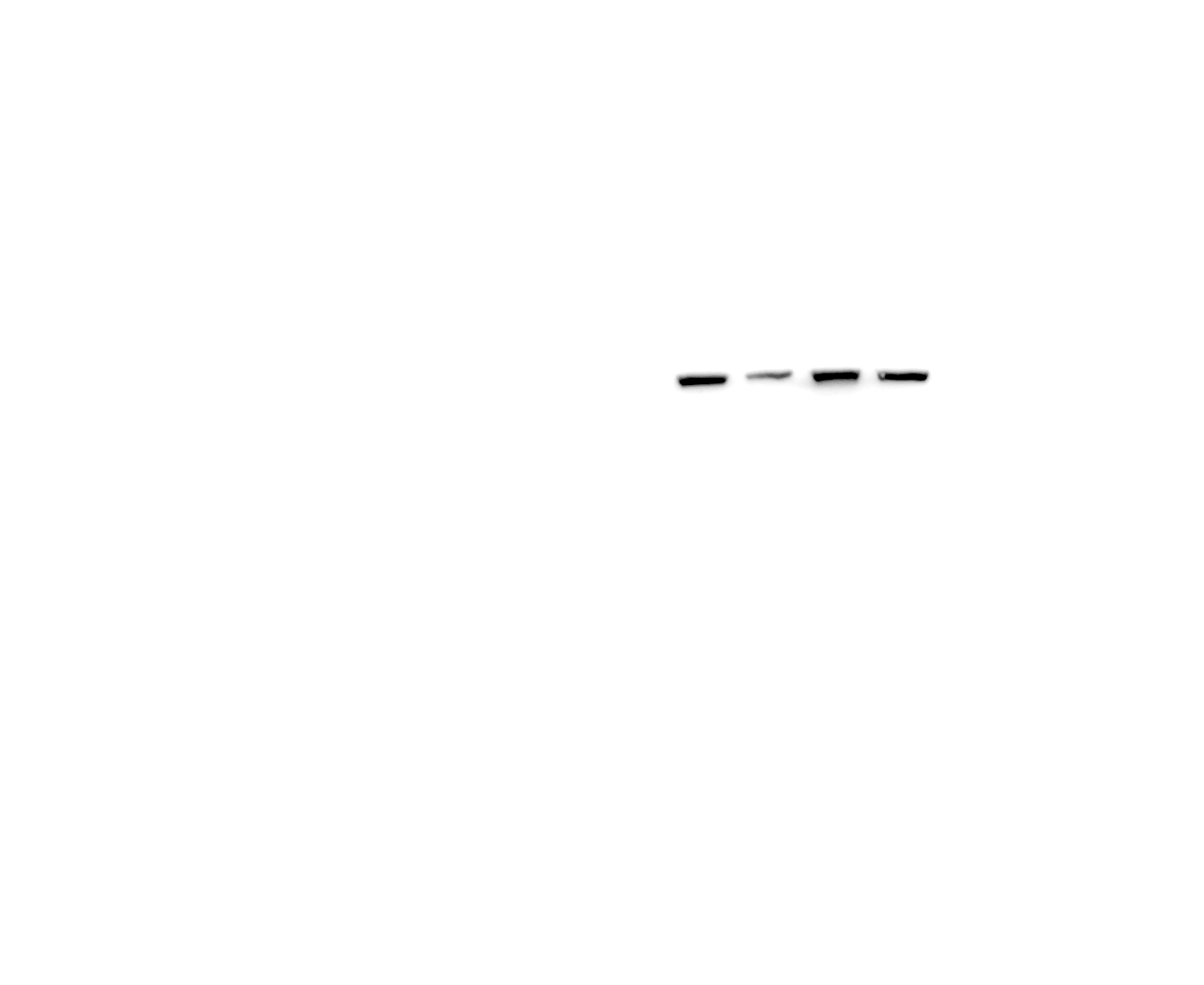

Supplement: Supplemental Information 6 [file peerj-11-15458-s006.zip › Fig.6/a┬-catenin 06.png]

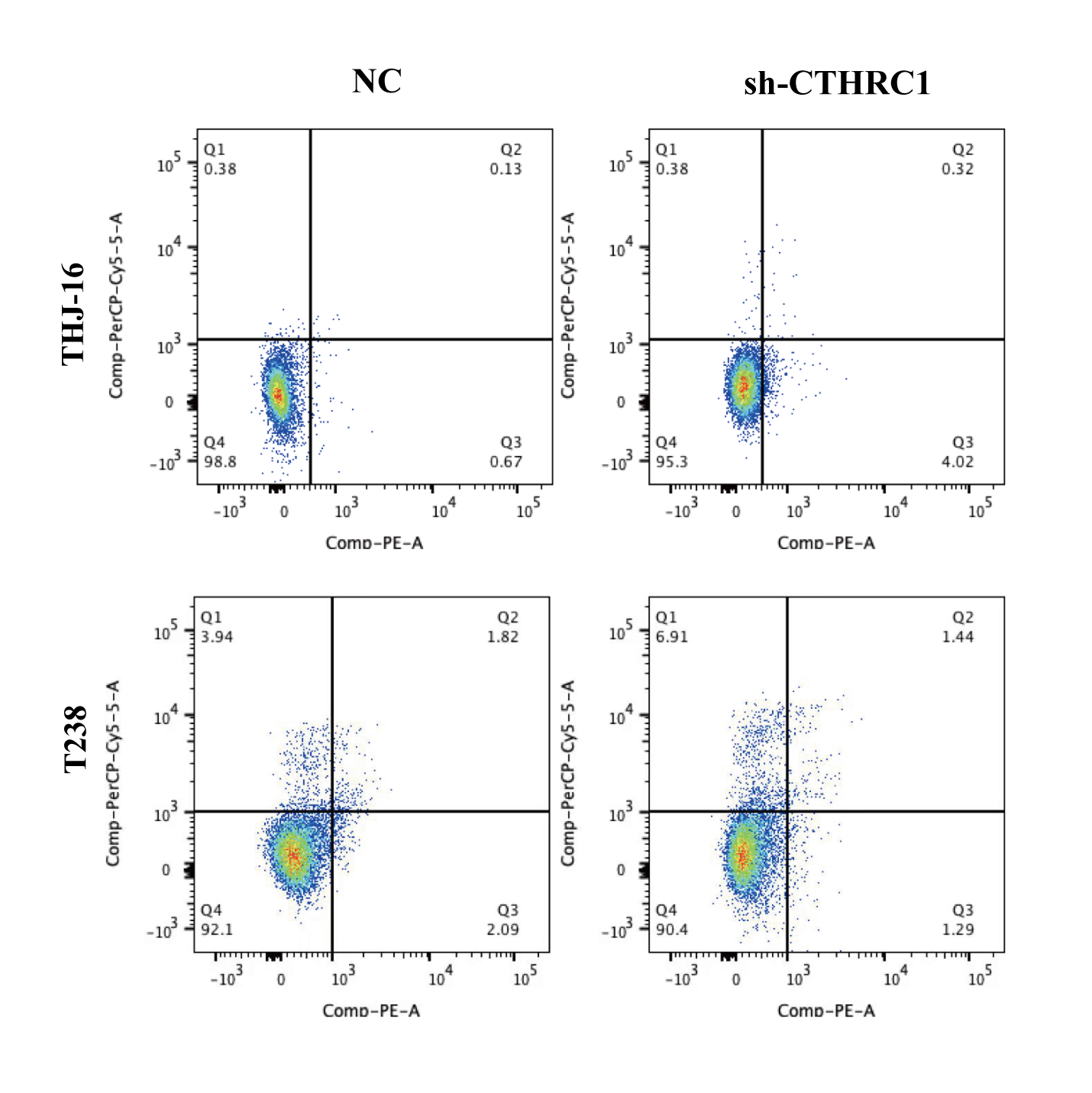

Supplement: Supplemental Information 7 [file peerj-11-15458-s007.png]
