# Supplementary material for: CTHRC1 promotes anaplastic thyroid cancer progression by upregulating the proliferation, migration, and invasion of tumor cells
Source: PeerJ. 2023 May 29;11:e15458. doi: 10.7717/peerj.15458 (PMC10234271; doi:10.7717/peerj.15458)
Supplement: Supplemental Information 8 [file peerj-11-15458-s008.docx]

|  | **Sequence variations** |
| --- | --- |
| THJ-16T | - Gene fusion; HGNC; [1097](https://www.genenames.org/data/gene-symbol-report/#!/hgnc_id/HGNC:1097); BRAF + HGNC; [7112](https://www.genenames.org/data/gene-symbol-report/#!/hgnc_id/HGNC:7112); MKRN1; Name(s)=MKRN1-BRAF; Note=In frame (PubMed=[26680454](https://www.ncbi.nlm.nih.gov/pubmed/26680454); PubMed=[30737244](https://www.ncbi.nlm.nih.gov/pubmed/30737244)). - Mutation; HGNC; [8975](https://www.genenames.org/data/gene-symbol-report/#!/hgnc_id/HGNC:8975); PIK3CA; Simple; p.Glu545Lys (c.1633G>A); ClinVar=[VCV000013655](https://www.ncbi.nlm.nih.gov/clinvar/variation/VCV000013655); Zygosity=Heterozygous (PubMed=[23833040](https://www.ncbi.nlm.nih.gov/pubmed/23833040); PubMed=[26680454](https://www.ncbi.nlm.nih.gov/pubmed/26680454); PubMed=[30737244](https://www.ncbi.nlm.nih.gov/pubmed/30737244)). - Mutation; HGNC; [3373](https://www.genenames.org/data/gene-symbol-report/#!/hgnc_id/HGNC:3373); EP300; Simple; p.Ser799Phefs*5 (c.2396delC); Zygosity=Homozygous (PubMed=[26680454](https://www.ncbi.nlm.nih.gov/pubmed/26680454); PubMed=[30737244](https://www.ncbi.nlm.nih.gov/pubmed/30737244)). - Mutation; HGNC; [9967](https://www.genenames.org/data/gene-symbol-report/#!/hgnc_id/HGNC:9967); RET; Simple; p.Glu90Lys (c.268G>A); Zygosity=Heterozygous (PubMed=[26680454](https://www.ncbi.nlm.nih.gov/pubmed/26680454); PubMed=[30737244](https://www.ncbi.nlm.nih.gov/pubmed/30737244)). - Mutation; HGNC; [11730](https://www.genenames.org/data/gene-symbol-report/#!/hgnc_id/HGNC:11730); TERT; Simple; c.1-124C>T (c.228C>T) (C228T); Zygosity=Heterozygous; Note=In promoter (PubMed=[23833040](https://www.ncbi.nlm.nih.gov/pubmed/23833040); PubMed=[30737244](https://www.ncbi.nlm.nih.gov/pubmed/30737244)). - Mutation; HGNC; [11998](https://www.genenames.org/data/gene-symbol-report/#!/hgnc_id/HGNC:11998); TP53; Simple; p.Arg273His (c.818G>A); ClinVar=[VCV000012366](https://www.ncbi.nlm.nih.gov/clinvar/variation/VCV000012366); Zygosity=Homozygous (PubMed=[30737244](https://www.ncbi.nlm.nih.gov/pubmed/30737244)). |
| T238 | - Mutation; HGNC; [1097](https://www.genenames.org/data/gene-symbol-report/#!/hgnc_id/HGNC:1097); BRAF; Simple; p.Val600Glu (c.1799T>A); ClinVar=[VCV000013961](https://www.ncbi.nlm.nih.gov/clinvar/variation/VCV000013961); Zygosity=Unspecified (PubMed=[23833040](https://www.ncbi.nlm.nih.gov/pubmed/23833040); PubMed=[30737244](https://www.ncbi.nlm.nih.gov/pubmed/30737244)). - Mutation; HGNC; [1787](https://www.genenames.org/data/gene-symbol-report/#!/hgnc_id/HGNC:1787); CDKN2A; Simple; p.Leu63Arg (c.188T>G); Zygosity=Homozygous (PubMed=[30737244](https://www.ncbi.nlm.nih.gov/pubmed/30737244)). - Mutation; HGNC; [8975](https://www.genenames.org/data/gene-symbol-report/#!/hgnc_id/HGNC:8975); PIK3CA; Simple; p.Glu542Lys (c.1624G>A); ClinVar=[VCV000031944](https://www.ncbi.nlm.nih.gov/clinvar/variation/VCV000031944); Zygosity=Heterozygous (PubMed=[23833040](https://www.ncbi.nlm.nih.gov/pubmed/23833040); PubMed=[30737244](https://www.ncbi.nlm.nih.gov/pubmed/30737244)). - Mutation; HGNC; [11730](https://www.genenames.org/data/gene-symbol-report/#!/hgnc_id/HGNC:11730); TERT; Simple; c.1-124C>T (c.228C>T) (C228T); Zygosity=Heterozygous; Note=In promoter (PubMed=[23833040](https://www.ncbi.nlm.nih.gov/pubmed/23833040); PubMed=[30737244](https://www.ncbi.nlm.nih.gov/pubmed/30737244)). - Mutation; HGNC; [11998](https://www.genenames.org/data/gene-symbol-report/#!/hgnc_id/HGNC:11998); TP53; Simple; p.Ser183Ter (c.548C>G); ClinVar=[VCV000634701](https://www.ncbi.nlm.nih.gov/clinvar/variation/VCV000634701); Zygosity=Homozygous (PubMed=[30737244](https://www.ncbi.nlm.nih.gov/pubmed/30737244)). |
| SW1736 | - Mutation; HGNC; [1097](https://www.genenames.org/data/gene-symbol-report/#!/hgnc_id/HGNC:1097); BRAF; Simple; p.Val600Glu (c.1799T>A); ClinVar=[VCV000013961](https://www.ncbi.nlm.nih.gov/clinvar/variation/VCV000013961); Zygosity=Heterozygous (PubMed=[23833040](https://www.ncbi.nlm.nih.gov/pubmed/23833040); PubMed=[30737244](https://www.ncbi.nlm.nih.gov/pubmed/30737244)). - Mutation; HGNC; [11730](https://www.genenames.org/data/gene-symbol-report/#!/hgnc_id/HGNC:11730); TERT; Simple; c.1-124C>T (c.228C>T) (C228T); Zygosity=Heterozygous; Note=In promoter (PubMed=[23833040](https://www.ncbi.nlm.nih.gov/pubmed/23833040); PubMed=[30737244](https://www.ncbi.nlm.nih.gov/pubmed/30737244)). - Mutation; HGNC; [11998](https://www.genenames.org/data/gene-symbol-report/#!/hgnc_id/HGNC:11998); TP53; Simple; p.Gln192Ter (c.574C>T); ClinVar=[VCV000406579](https://www.ncbi.nlm.nih.gov/clinvar/variation/VCV000406579); Zygosity=Homozygous (PubMed=[30737244](https://www.ncbi.nlm.nih.gov/pubmed/30737244)). - Mutation; HGNC; [12373](https://www.genenames.org/data/gene-symbol-report/#!/hgnc_id/HGNC:12373); TSHR; Simple; p.Ile486Phe (c.1456A>T); Zygosity=Heterozygous (PubMed=[30737244](https://www.ncbi.nlm.nih.gov/pubmed/30737244)). |
