# Supplementary material for: CTHRC1 promotes anaplastic thyroid cancer progression by upregulating the proliferation, migration, and invasion of tumor cells
Source: PeerJ. 2023 May 29;11:e15458. doi: 10.7717/peerj.15458 (PMC10234271; doi:10.7717/peerj.15458)
Supplement: Supplemental Information 9 [file peerj-11-15458-s009.zip › STR/STR Nthy-ori 3-1.pdf]

细胞遗传质量鉴定检验报告

检品名称：细胞系

检验类型：STR 基因型检验

样品编号：

表 1 样本编号

客户样本编号	公司编号
297	20170721-04

样品数量：1

样品性状：细胞系

检测项目：STR

送检单位：上海富衡生物科技有限公司

检测方法：用 Axygen 的基因组抽提试剂盒提取 DNA，采用 20- STR 扩增方案扩增，在 ABI 3730XL 型遗传分析仪上对 STR 位点和性别基因 Amelogenin 进行检测。

检验结果：

(一) 检验基本情况

表 2：样本基因型检验结果

	多等位基因	匹配细胞系	细胞库	EV 值	匹配说明
20170721-04	无	Nthy-ori 3-1	EXPASY	1.00	完全匹配

- 多等位基因指三等位及以上基因现象。
- 本次检测各细胞分型结果良好。

(二) 各样本描述

- 20170721-04：该株细胞 DNA 分型在细胞系检索中**找到完全匹配**的细胞系，expasy 数据库显示细胞名为 **Nthy-ori 3-1**，细胞号对应 **ECACC; 90011609**。，本次检测在该细胞系中**没有发现多等位基因**。

(三) 样本分型结果

细胞 20170721-04 的 STR 位点和 Amelogenin 位点的基因分型结果						
Loci	送检细胞 STR 信息			细胞库细胞 STR 信息		
	送检细胞名：297			细胞库细胞名：Nthy-ori 3-1		
	Allele1	Allele2	Allele3	Allele1	Allele2	Allele3
D5S818	11	11		11	11	
D13S317	11	11		11	11	
D7S820	7	12		7	12	
D16S539	12	13		12	13	
VWA	16	18		16	18	
TH01	7	7		7	7	
AMEL	X	X		X	X	
TPOX	9	9		9	9	
CSF1PO	12	12		12	12	
D12S391	18	20				

FGA	21	22				
D2S1338	20	26				
D21S11	29	30				
D18S51	14	14				
D8S1179	12	12				
D3S1358	14	16				
D6S1043	11	11				
PENTAE	7	13				
D19S433	16.2	16.2				
PENTAD	8	13				

其他说明：

(一) 分型方案及位点分布：

附表：实验方案及位点

	方案 1	方案 2	方案 3	方案 4
1	TH01	TPOX	D3S1358	AMEL
2	D12S391	VWA	D13S317	D5S818

3	D7S820	D8S1179	D6S104<br>3	D2S1338
4	CSF1PO	PENTAD	D16S53<br>9	D21S11
5	FGA		D19S43<br>3	D18S51
6	PENTAE			

Certificate of STR Analysis

Cell Line Authentication 17

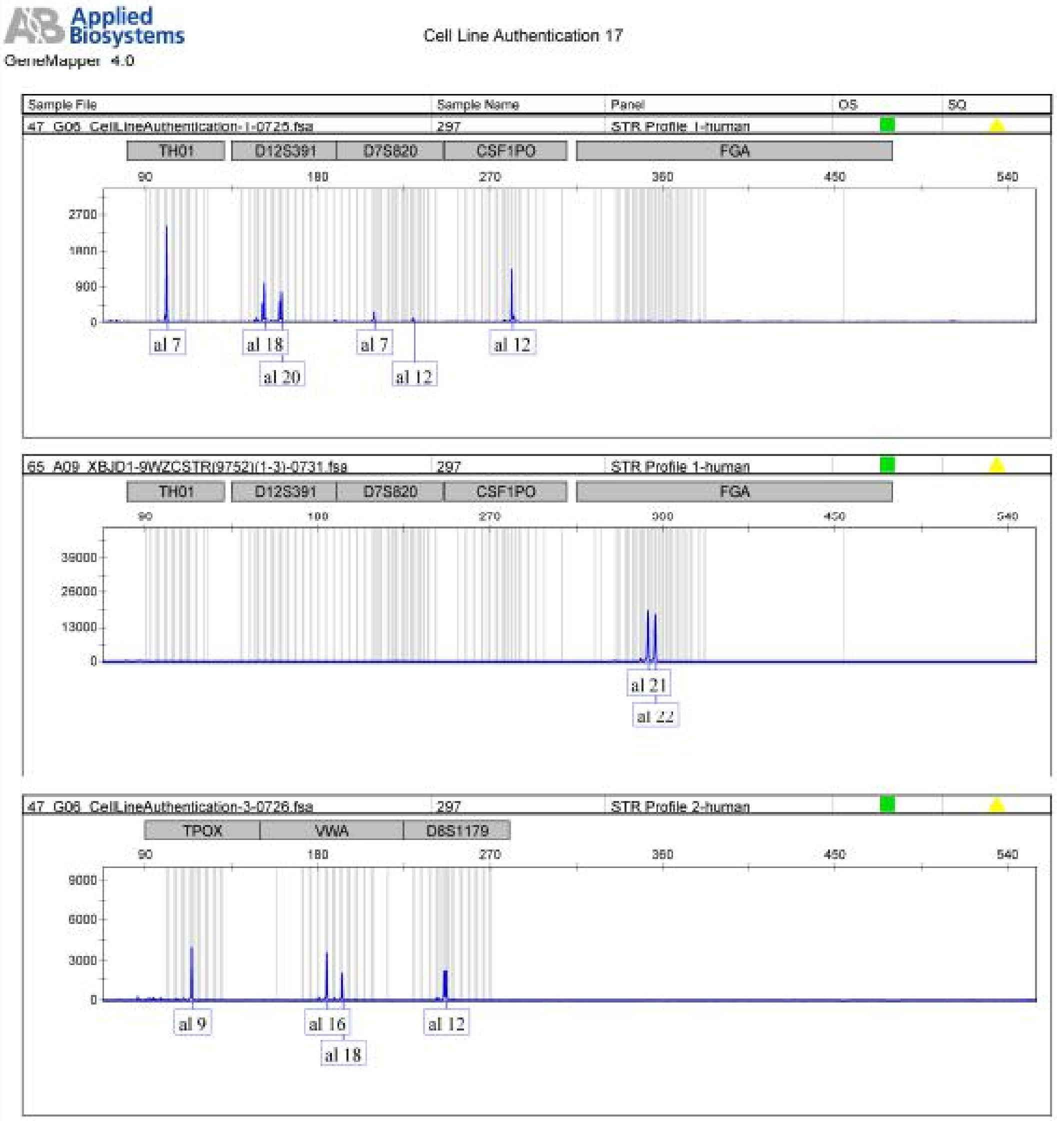

Certificate of STR Analysis

Applied Biosystems
GeneMapper 4.0

Cell Line Authentication 17

Sample File	Sample Name	Panel	OS	SQ
47_G06_CellLineAuthentication-4-0725.fsa	297	STR Profile 3-human		

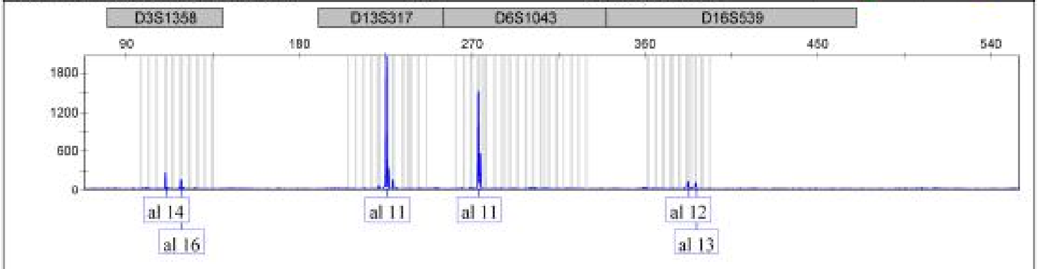

Sample File	Sample Name	Panel	OS	SQ
23_G03_XBJD0721Pi-2(4-11)XiaoShi-0725.fsa	297	STR Profile 4-human		

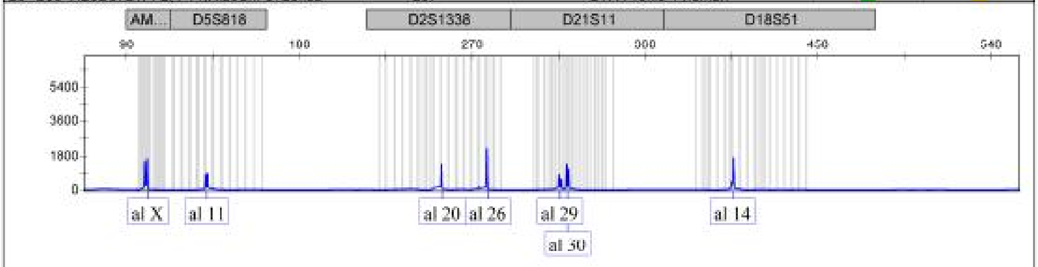

Certificate of STR Analysis

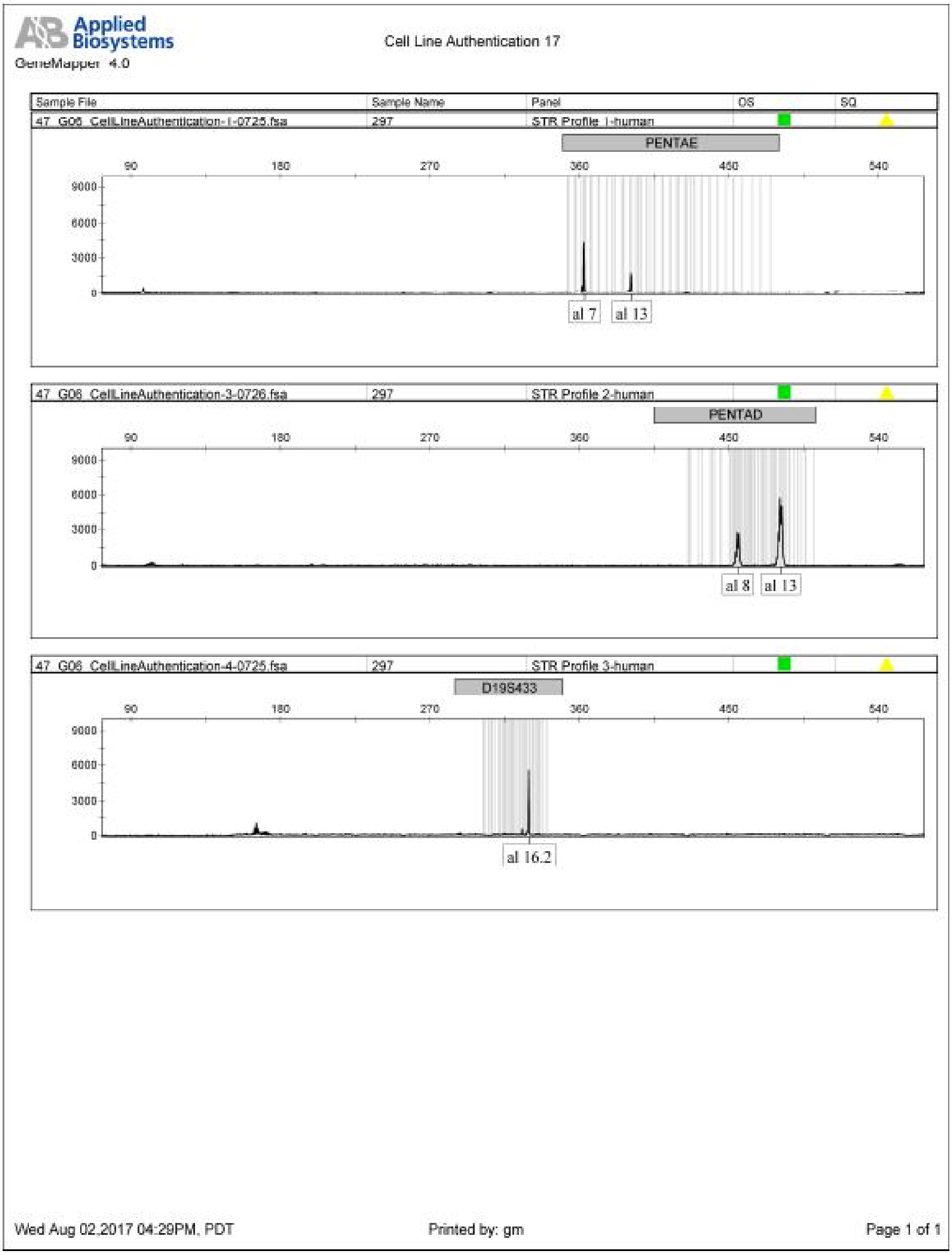

签发日期：

2017 年 08 月
