# Supplementary material for: CTHRC1 promotes anaplastic thyroid cancer progression by upregulating the proliferation, migration, and invasion of tumor cells
Source: PeerJ. 2023 May 29;11:e15458. doi: 10.7717/peerj.15458 (PMC10234271; doi:10.7717/peerj.15458)
Supplement: Supplemental Information 9 [file peerj-11-15458-s009.zip › STR/STR-THJ-16T.pdf]

细胞 STR 分型检验报告

Report of Cell Line Identification

单位/Applicant:	天津医科大学总医院
样本编号/ Sample No.:	THJ-16T
待检测细胞系名称/Name of cell line:	THJ-16T
样本数量及规格/ Sample Spec.:	细胞沉淀 1 个/ Cell precipitation
样本接收日期/ Sample Receive Date:	20230417
报告编号/ Report No:	VCPO20230413002STR01

1. **测试要求/**Service Description

鉴定该细胞样品是否存在交叉污染现象，并与数据库比对 DNA 分型数据确认来源。

Detection of human origin intra-species cross-contamination. Database search and analysis to identify cell origin of sample using recognized repository .

2. **检材处理和检验方法/**Method and Procedure

取适量检材用莱枫痕量试剂盒提取 DNA，采用人类 STR 扩增荧光检测试剂盒进行复合 PCR 扩增，在 ABI 3730xl 型遗传分析仪上对 STR 位点和性别基因 Amelogenin 进行检测。

Cellular DNA is purified with lifefeng DNA kit. PCR is amplified with Human STR Identification Kit. PCR products are assayed with 3730xl DNA Analyzer (Applied Biosystems).

3. **检验结果/**STR Profiles

该细胞株的 STR 位点和 Amelogenin 位点的基因分型结果见附表 1，分型图谱见附图 1。

The STR profiles of the cell line sample are summarized in Table 1 and Figure 1.

4. **检验结论/**Result & Analysis

样本编号/Sample No.THJ-16T

1) 用 GeneMapperID-X 1.4 software(ABI)对各 STR 位点进行基因型分析。该细胞 DNA 扩增后图谱清晰，分型结果良好。
   STR Typing profile is analyzed with GeneMapper ID-X 1.4 software (Applied Biosystems) (Table 1 and Figure 1).
2) 性别基因 Amelogenin：X。
3) 该株细胞 DNA 进行细胞 STR 分型结果显示（如图 1），未出现多等位基因。
   Normal peaks distribution was observed (Figure 1).
4) 该株细胞 DNA 分型在 Expasy 数据库中找到与其细胞分型 100%匹配的细胞(THJ-16T, P/N: CVCL_W920, 图 2,3）。
   100% matched cell line (THJ-16T, P/N: CVCL_W920) is found in Expasy data bank (Figure 2,3).

审核人/Reviewed by: 杨小丽/Xiaoli Yang

Shanghai VivaCell Biosciences Ltd. 上海此龙田生物科技有限公司 检验专用章

表 1：样本 THJ-16T 的 STR 位点和 Amelogenin 位点的基因分型结果

Table 1: STR profiles of Sample

	**Sample**<br>**THJ-16T**	**Source: Expasy**<br>THJ-16T, 100% Match
**Marker**	**Allele**	**Allele**
D5S818	11,12	11,12
D13S317	10,11	10,11
D7S820	10,12	10,12
D16S539	9,12	9,12
VWA	16,17	16,17
TH01	8,9	8,9
AMEL	X	X
TPOX	8,11	8,11
CSF1PO	11,14	11,14
D12S391	22	
FGA	23,27	
D2S1338	18,19	
D21S11	29,31	
D18S51	17,19	
D8S1179	12,13	
D3S1358	15,16	
D6S1043	12,18	
PENTAE	10	
D19S433	14,15	
PENTAD	12	
D1S1656	16.3,18.3	

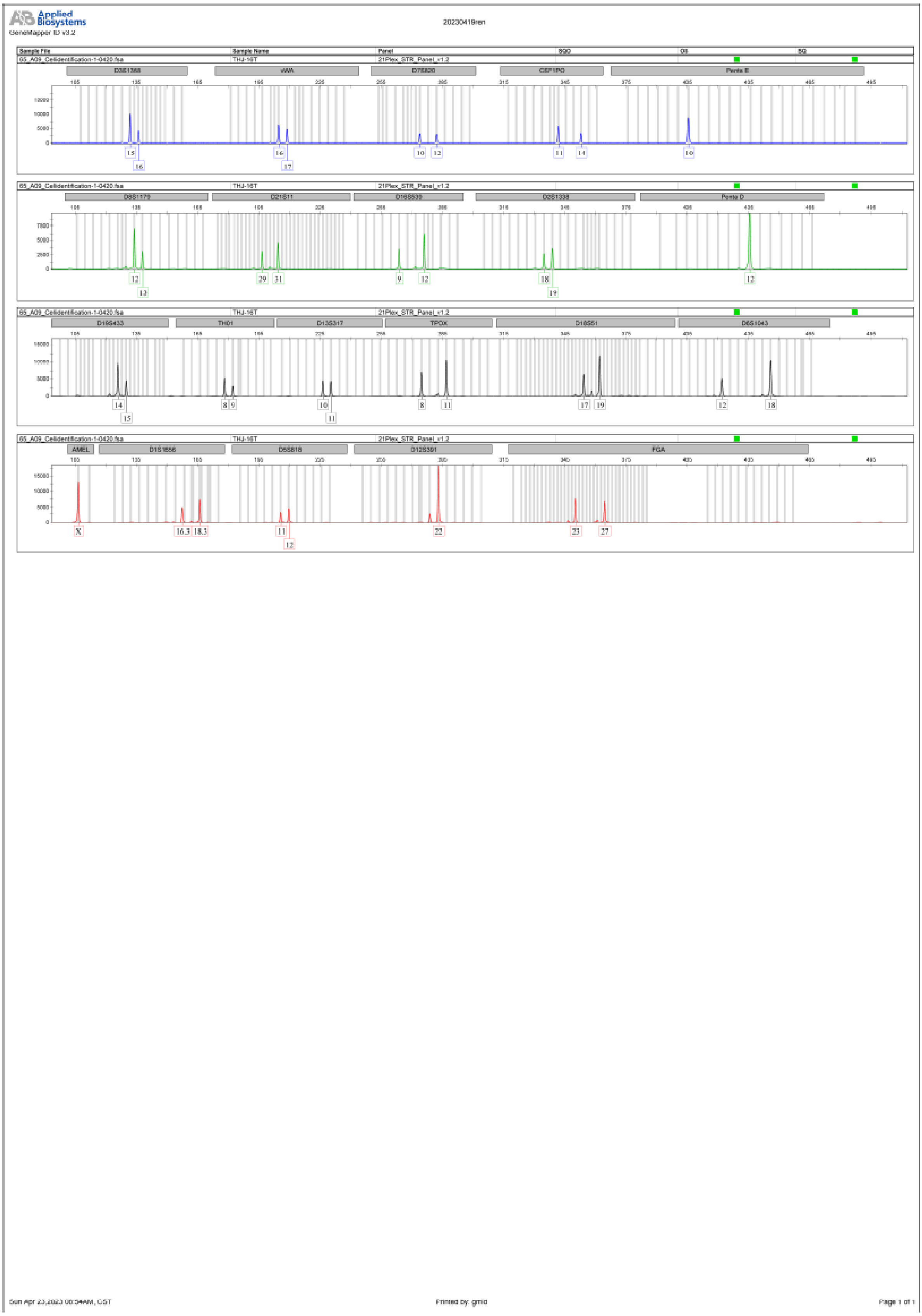

图 1/Figure 1：样本 THJ-16T 的 STR 位点和 Amelogenin 位点的基因分型图（STR profiles of Sample）

Similarity	Cell line	Source	Shared	D5S818	D7S820	D13S317	D16S539	vWA	TH01	TPOX	CSF1PO	Amelogenin
?		Your query		11, 12	10, 11	10, 12	9, 12	16, 17	8, 9	8, 11	11, 14	X, X
77.8 %	Hs 517.Sk	CRL-7311	9	11, 12	8, 11	11, 12	9, 12	16, 17	8, 9	8, 8	10, 11	X, X
77.8 %	THEECs	CRL-4026	9	11, 12, 13	9, 10, 11, 12	8, 10, 12, 13	9, 12	14, 15, 18, 19	7, 8, 9	8, 10	10, 11	X, X
77.8 %	IST-1	CRL-2734	9	11, 12	9, 10, 13, 14	8, 11, 12	10, 12, 13	16, 17, 18	7, 8, 9, 9.3	6, 8, 9, 11	10, 11, 12	X, X
72.2 %	TE03	USNIH006	9	11, 12	10, 11	12, 14	11, 12	16, 17	7, 9	8, 8	11, 12	X, X
72.2 %	SLVL	RCB1702	9	11, 12	10, 11	8, 10	12, 12	17, 18	8, 9	8, 8	11, 12	X, X
72.2 %	WS2TKB	RCB0407	9	11, 12	10, 12	12, 14	9, 12	17, 18	7, 9	8, 11	11, 12	X, X
72.2 %	UACC-62	NCI-60 panel	9	10, 12	8, 11	12, 12	9, 12	16, 17	6, 9	8, 11	10, 11	X, X
72.2 %	SNU-902	KCLB Korea 902	9	11, 11	10, 11	10, 12	12, 12	16, 17	9, 9	8, 9	11, 11	X, X
72.2 %	Hs 507.Sk	CRL-7307	9	11, 12	10, 11	11, 12	12, 12	16, 17	8, 9	8, 9	10, 12	X, X
72.2 %	Kasumi-4	CRL-2726	9	11, 12	8, 10	10, 12	9, 13	16, 18	8, 9	8, 11	10, 12	X, X
72.2 %	KASUMI-4	DSMZ: ACC-715	9	11, 12	8, 10	10, 12	9, 13	16, 18	8, 9	8, 11	10, 12	X, X
66.7 %	ACJ Cells No. 35	STRJ0035	9	11, 11	10, 12	12, 12	11, 12	17, 17	8, 9	8, 11	11, 12	X, X
66.7 %	ACHN	RCB1962	9	12, 12	9, 11	12, 12	12, 13	16, 17	8, 8	8, 11	11, 11	X, X
66.7 %	JHCOLOYI	RCB1706	9	12, 13	10, 12	11, 12	9, 9	16, 17	7, 9	8, 11	9, 11	X, X
66.7 %	T3M-3	RCB1018	9	11, 11	10, 10	8, 12	9, 12	14, 16	6, 8	8, 11	10, 11	X, X
66.7 %	SF8536	RCB0614	9	11, 12	10, 11	8, 9	9, 12	16, 17	7, 10	8, 8	11, 12	X, X
66.7 %	HUC-F2	RCB0436	9	11, 11	10, 10	10, 12	12, 12	17, 20	9, 9	8, 11	10, 14	X, X
66.7 %	U-87MG Uppsala	PubMed=27582061	9	11, 12	10, 13	11, 12	9, 13	15, 19	8, 9	8, 11	10, 11	X, X
66.7 %	a552	PSCCA0552	9	11, 11	10, 11	10, 12	9, 10	16, 18	8, 9	8, 8	9, 12	X, X
66.7 %	HOP-62	NCI-60 panel	9	11, 11	10, 12	12, 12	11, 12	15, 17	8, 9	8, 11	11, 12	X, X

图 2/ Figure 2：样本 THJ-16T 与 DSMZ 数据对比分析图（Sample Comparison to the DSMZ STR Profile Database）

Markers:

Amelogenin	X
CSF1PO	11,14
D3S1358	15,16
D5S818	11,12
D7S820	10,12
D8S1179	12,13
D13S317	10,11
D16S539	9,12
D18S51	17,19
D21S11	29,31
FGA	23,27
TH01	8,9
TPOX	8,11
vWA	16,17

图 3/ Figure 3： Expasy 数据库 THJ-16T 数据（Expasy STR Profile Database）
